# Synthetic mixed-signal computation in living cells

**DOI:** 10.1038/ncomms11658

**Published:** 2016-06-03

**Authors:** Jacob R. Rubens, Gianluca Selvaggio, Timothy K. Lu

**Affiliations:** 1 ^1^Synthetic Biology Group, MIT Synthetic Biology Center, Research Laboratory of Electronics, Department of Electrical Engineering and Computer Science, Massachusetts Institute of Technology, Cambridge, Massachusetts 02139, USA; 2 Department of Biological Engineering, Massachusetts Institute of Technology, Cambridge, Massachusetts 02139, USA; 3 Microbiology Program, Massachusetts Institute of Technology, Cambridge, Massachusetts 02139, USA; 4 Computational and System Biology Group, Centre for Neuroscience and Cell Biology, University of Coimbra, 3004-517 Coimbra, Portugal; 5 The Center for Microbiome Informatics and Therapeutics, Cambridge, Massachusetts 02139, USA

## Abstract

Living cells implement complex computations on the continuous environmental signals that they encounter. These computations involve both analogue- and digital-like processing of signals to give rise to complex developmental programs, context-dependent behaviours and homeostatic activities. In contrast to natural biological systems, synthetic biological systems have largely focused on either digital or analogue computation separately. Here we integrate analogue and digital computation to implement complex hybrid synthetic genetic programs in living cells. We present a framework for building comparator gene circuits to digitize analogue inputs based on different thresholds. We then demonstrate that comparators can be predictably composed together to build band-pass filters, ternary logic systems and multi-level analogue-to-digital converters. In addition, we interface these analogue-to-digital circuits with other digital gene circuits to enable concentration-dependent logic. We expect that this hybrid computational paradigm will enable new industrial, diagnostic and therapeutic applications with engineered cells.

## Abstract (web summary)

Digital and analogue gene circuits each have distinct advantages in natural and engineered cells. Here, Rubens *et al*. engineer synthetic gene circuits that implement mixed-signal digital and analogue computations in living cells.

## Graphical abstract

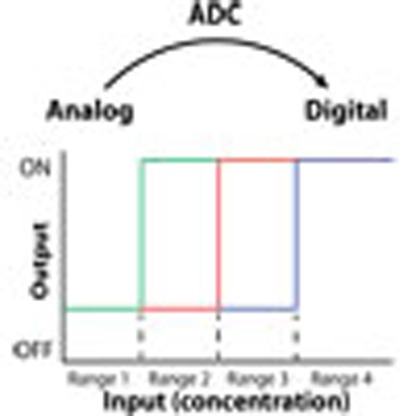

Analogue and digital computation each have distinct advantages for cellular computing^1^. Digital computation in synthetic^2^,^3^,^4^,^5^,^6^,^7^,^8^ and natural biological systems is useful for signal integration given its relative robustness to noise^9^ and is exemplified by decision-making circuits, such as those in developmental programs that lead cells into differentiated states^10^. Analogue computation is useful for signal processing in synthetic^11^,^12^,^13^,^14^ or natural biological systems when the output needs to be dependent on graded information or continuous functions of the inputs, such as the sum or ratio of energy sources or signalling molecules^15^,^16^. However, analogue signal integration is susceptible to noise, making it challenging to design robust synthetic genetic programs^17^. Here we combine the benefits of analogue signal processing with digital signal integration to create artificial mixed-signal gene networks that carry out new hybrid functions in living cells^18^.

Our approach is to process signals from front-end analogue sensors with composable input-discretization devices that are analogous to electronic comparators. The outputs of these devices can then be processed in a digital manner with downstream circuits. This strategy of explicitly digitizing analogue signals followed by digital computing stages is conceptually different than other mixed-signal computing approaches, such as fuzzy logic, neural networks and hybrid automata, in which analogue and digital processing are intricately coupled. However, the components developed here may be useful for future gene circuits implementing these other forms of hybrid computing. Electronic comparators compare analogue voltages between two terminals (V_+_ and V_−_) and output a digital OFF or ON signal (or ‘LO' or ‘HI') if V_+_<V_−_ or V_+_>V_−_, respectively^19^. Rather than voltage, our genetic comparators take the concentration of an activated transcription factor as their input. The transcription factor acts a front-end sensor for continuous information (for example, the concentration of a small molecule), and should ideally operate over a wide input dynamic range to enable multiple genetic comparators with different thresholds to discretize the same input into multiple distinct outputs. In contrast to previously developed thresholding circuits that modulate continuous levels of gene expression in response to molecular concentration and could be used as comparators^20^,^21^,^22^,^23^,^24^, our comparators convert molecular concentration into digital gene expression. This enabled us to create higher-order mixed-signal circuits that also take on digital gene expression states, such as 2-bit analogue-to-digital converters (ADCs) and ternary logic circuits, in contrast to previous mixed-signal circuits, such as filters that are essentially 1-bit ADCs^25^,^26^,^27^,^28^.

## Results

### Genetic comparators digitize analogue gene expression

We first created an analogue sensor for the reactive oxygen species hydrogen peroxide (H_2_O_2_). H_2_O_2_ plays intricate biological roles across all kingdoms of life, and its regulation is linked to human health and disease^29^. H_2_O_2_ oxidizes and activates the *Escherichia coli* transcription factor OxyR^30^,^31^,^32^,^33^. We constitutively expressed OxyR to set a minimum concentration of OxyR in the cell, since genomically expressed *oxyR* is auto-negatively regulated, and we placed *gfp* under the control of the OxyR-regulated oxyS promoter (oxySp) on the same low-copy plasmid (Supplementary Fig. 1). We found that green fluorescent protein (GFP) expression was continuously increased by H_2_O_2_ over more than two orders of magnitude of concentration, indicating that OxyR is a wide-dynamic-range analogue sensor for H_2_O_2_ in this context.

We then created genetic comparators (Supplementary Fig. 2), which can be conceptualized as composed of three elements. The first component is the threshold module. It includes a promoter, which is regulated by the transcription factor, and a ribosome-binding site (RBS) that together set the expression level of the downstream recombinase gene and determine the threshold for comparator activation. This is in contrast to electronic comparators, where a second input can dynamically set the threshold (for example, V_−_). The second module is the digitization module, which is composed of a recombinase whose expression is controlled by the threshold module. The recombinase digitizes the input value by inverting the orientation of a targeted DNA segment maintained at a very-low-copy number. The third module is the DNA that is inverted by the recombinase, which can contain a gene or gene-regulatory elements, such as transcriptional promoters or terminators, to alter expression of the desired output(s).

The digitization aspect of the comparator relies on recombinases, and thus we explored how the number of sites targeted by recombinases affects signal digitization into two distinct gene expression states within individual cells. The serine integrases (recombinases) we used flip, excise or integrate DNA depending on the orientation of *attB* and *attP* recombinase-recognition sites, and their activity is unidirectional unless co-factors are present^34^. Recombinases have been used to build digital counters^35^, integrate logic and memory^36^, and amplify input–output transfer functions^37^. To discretize H_2_O_2_ input levels, we placed the Bxb1 recombinase under the control of the oxySp promoter on a low-copy plasmid. To keep the basal level of *bxb1* minimal such that there is little recombinase activity in the cell in the uninduced state, we added a ClpXP-mediated degradation tag to the 3′ end of the *bxb1*-coding sequence^38^ (Supplementary Fig. 3a). We tested two options as reporters for recombinase activity: a medium-copy plasmid (MCP, maintained at 20–30 copies per cell^39^) and a bacterial artificial chromosome (BAC, maintained at 1–2 copies per cell^40^), each of which contained a constitutive promoter upstream of an inverted *gfp* gene flanked by oppositely oriented *attB* and *attP* sites.

We induced *bxb1* expression at different concentrations of H_2_O_2_ and measured GFP expression via flow cytometry (Supplementary Fig. 3b–d). We set a threshold for calling cells GFP ‘ON' or ‘OFF', and used this threshold to calculate the per cent of cells that was ON (%ON) at each concentration of H_2_O_2_ (Supplementary Note 1). The %ON versus H_2_O_2_ concentration data were fit to a sigmoidal function to generate input–output transfer functions (Supplementary Note 1; Supplementary Table 3). The MCP and BAC reporters had similar transfer functions, although cells using the MCP reporter had a higher per cent of cells ON at the basal H_2_O_2_ concentration (Supplementary Fig. 3b). However, GFP expression in cells with the MCP reporter exhibited a multi-modal distribution especially at intermediate concentrations of H_2_O_2_, which suggests partial plasmid flipping and thus mixed GFP expression levels in different cells (Supplementary Fig. 3d). This effect was further demonstrated by increases in the geometric mean of GFP levels with increasing H_2_O_2_ in the ON population (Supplementary Fig. 3f). In contrast, cells with the BAC reporter only exhibited a bi-modal distribution (Supplementary Fig. 3c), and the geometric mean of the ON population only marginally increased with H_2_O_2_ concentration (Supplementary Fig. 3e). Thus, we concluded that the BAC reporter converts the input concentration of H_2_O_2_ into digital OFF and ON gene expression states within individual cells better than the MCP reporter.

We further sought to demonstrate that our analogue-to-digital comparator circuits could be used to drive downstream circuits in a *trans*-acting manner. To construct a cascade, we replaced *gfp* in the BAC expression operon with *tetR* and placed *gfp* under the control of the TetR-regulated promoter pLtetO on a MCP (Supplementary Fig. 4). In the absence of H_2_O_2_, the majority of cells expressed *gfp* and were in the ON state. In the presence of H_2_O_2_, *gfp* expression from pLtetO was efficiently repressed and the majority of cells were switched into an OFF state. These results demonstrate that recombinase circuits can be used together with *trans*-acting regulation to assemble functional cascades. We also developed a method to simplify the quantification of OFF versus ON, since fluorescent gene expression levels from the BAC are low and can result in overlapping OFF and ON gene expression distributions in flow cytometry. This method amplifies the copy number of the reporter from low to high, but preserves the bi-modal nature of the OFF and ON populations, thus confirming the digital flipping of the BAC (Supplementary Fig. 5).

**Figure 1:**
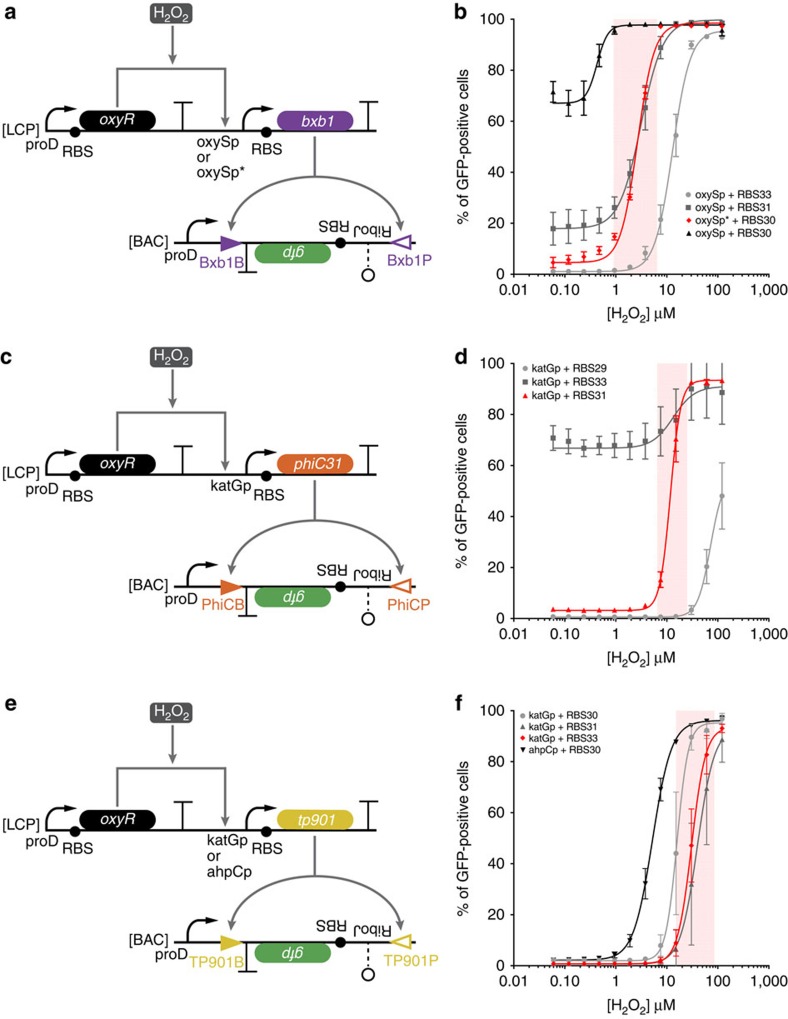
Genetic comparators with different activation thresholds. (**a**) The low-threshold H_2_O_2_ comparator circuit. OxyR is constitutively expressed from a low-copy plasmid (LCP) and activates transcription of *bxb1* recombinase from either the oxySp or oxySp* promoter on the same LCP in response to H_2_O_2_. *Bxb1* translation is altered by the strength of the ribosome-binding site (RBS). Bxb1 inverts the *gfp* expression cassette located between inversely oriented *attB* and *attP* sites (triangles) on a bacterial artificial chromosome (BAC), thus turning on GFP expression. The *gfp* cassette has a ribozyme sequence for cleaving the 5′-untranslated region of an mRNA transcript (RiboJ)^61^, a computationally designed RBS^62^, the *gfp*-coding sequence and a transcriptional terminator. (**b**) The per cent of GFP-positive cells at different H_2_O_2_ concentrations as measured by flow cytometry. Different combinations of the oxySp and oxySp* promoters, and RBSs exhibit different H_2_O_2_ thresholds and basal levels for GFP activation. The oxySp* and RBS30 combination (red diamonds) had the lowest threshold and a narrow transition band (shaded region). (**c**) The medium-threshold H_2_O_2_ comparator circuit. The same as **a**, except with the katGp promoter instead of the oxySp or oxySp* promoters, and *phiC31* recombinase and its *att* inversion sites instead of *bxb1* recombinase and its *att* inversion sites. (**d**) Different combinations of the katGp promoter and RBSs had different H_2_O_2_ thresholds and basal levels for GFP activation. The katGp and RBS31 combination (red triangles) had a medium H_2_O_2_ threshold and narrow transition band (shaded region). (**e**) The high-threshold H_2_O_2_ comparator circuit. The same as **a**, except with either the katGp promoter or ahpCp promoter instead of the oxySp or oxySp* promoters, and *tp901* recombinase and its *att* inversion sites instead of *bxb1* recombinase and its *att* inversion sites. (**f**) Different combinations of katGp and ahpCp promoters and RBSs exhibited different H_2_O_2_ thresholds for GFP activation. The katGp and RBS33 combination (red diamonds) had the highest threshold and a narrow transition band (shaded region). Lines are sigmoidal fits to the data (Supplementary Note 1). The errors (s.d.) are derived from flow cytometry experiments of three biological replicates, each of which involved *n*>30,000 gated events.

The threshold module of the comparator can be used to shift the discretization threshold. We created comparators with different thresholds and transition bands (for example, the input dynamic range) by assembling combinations of promoters with different transcription factor affinities, RBSs and recombinases (Fig. 1). We defined the transition band as the range of H_2_O_2_ concentrations across which the per cent of cells expressing the output fluorophore is between 10 and 90% as interpolated from the transfer function (though on a single cell level, gene expression is binary), and we calculated the ‘relative input range' of the transition band to define its width (Supplementary Note 1). A narrow relative input range is indicative of low variability across the cell population around the input threshold for state switching, which is important for robustness to noise^41^.

The low-threshold comparator used the Bxb1 recombinase and oxySp promoter, which is activated at low H_2_O_2_ concentrations. We screened different RBSs in this construct and found that none of these circuits turned ON below 1 μM H_2_O_2_ without also exhibiting a high basal level of recombinase activity (Fig. 1a). To address this issue and reduce basal *bxb1* expression, we used a strong RBS (RBS30) and randomly mutated the −10 region of the oxySp promoter to create a low-threshold comparator that had a transition band between 0.91 and 6.44 μM H_2_O_2_, giving it a relative input range of 7.10 (Fig. 1b; Supplementary Fig. 2a). To create a medium-threshold comparator, we tested different RBSs controlling *phiC31* recombinase translation from the katGp promoter (Fig. 1c). A circuit with RBS31 had a transition band of 6.50–25.13 μM, which is a relative input range of 3.87 (Fig. 1d; Supplementary Fig. 2b). To create a high-threshold comparator, we used *tp901* recombinase and screened different RBS and promoter combinations (Fig. 1e). We first tried the ahpCp promoter, but found that this promoter–recombinase combination had an intermediate activation threshold. We instead turned to the katGp promoter and tested different RBSs. Use of RBS33 yielded a circuit with improved behaviour, with a transition band of 15.19–85.49 μM H_2_O_2_ and relative input range of 5.63 (Fig. 1f; Supplementary Fig. 2c).

### Signal-processing circuits composed of genetic comparators

**Figure 2:**
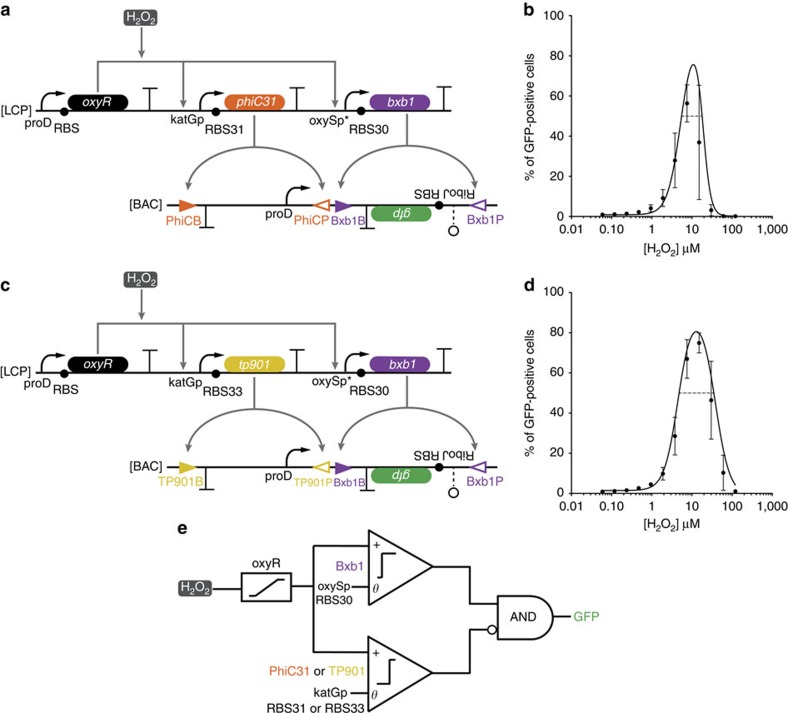
Band-pass filters assembled from low-pass and high-pass filters. (**a**) The low-threshold and medium-threshold band-pass filter circuit. OxyR is constitutively expressed and activates transcription of *bxb1* and *phiC31* in response to H_2_O_2_. Bxb1 inverts the *gfp* cassette to enable expression from the upright proD promoter, while PhiC31 inverts the proD promoter to turn off GFP production. (**b**) The per cent of GFP-positive cells at different H_2_O_2_ concentrations as measured by flow cytometry for the circuit shown in **a** (black circles). The transfer functions of the comparators composing the band-pass were characterized to generate the predicted band-pass transfer function (black line), *R*^2^=0.75 (Supplementary Fig. 7). The dashed black line demarcates the 50% ON relative input range. (**c**) The low-threshold and high-threshold band-pass filter circuit. Same as **a**, except RBS33 and *tp901* replace RBS31 and *phiC31*, respectively. (**d**) Same as **b**, but for the circuit shown in **c**. *R*^2^=0.95. The transfer functions of the comparators are shown in Supplementary Fig. 8. (**e**) Abstraction of band-pass genetic circuits. H_2_O_2_ activates OxyR in an analogue manner. Activated OxyR activates expression of *bxb1* and either *phiC31* or *tp901* depending on the circuit used (**a** or **c**, respectively). The activation threshold is set by the promoters and RBS-controlling recombinase expression. The expression of GFP is dependent on *bxb1* expression AND (NOT) *phiC31* or *tp901* expression. The errors (s.d.) are derived from flow cytometry experiments of three biological replicates, each of which involved *n*>30,000 gated events.

**Figure 3:**
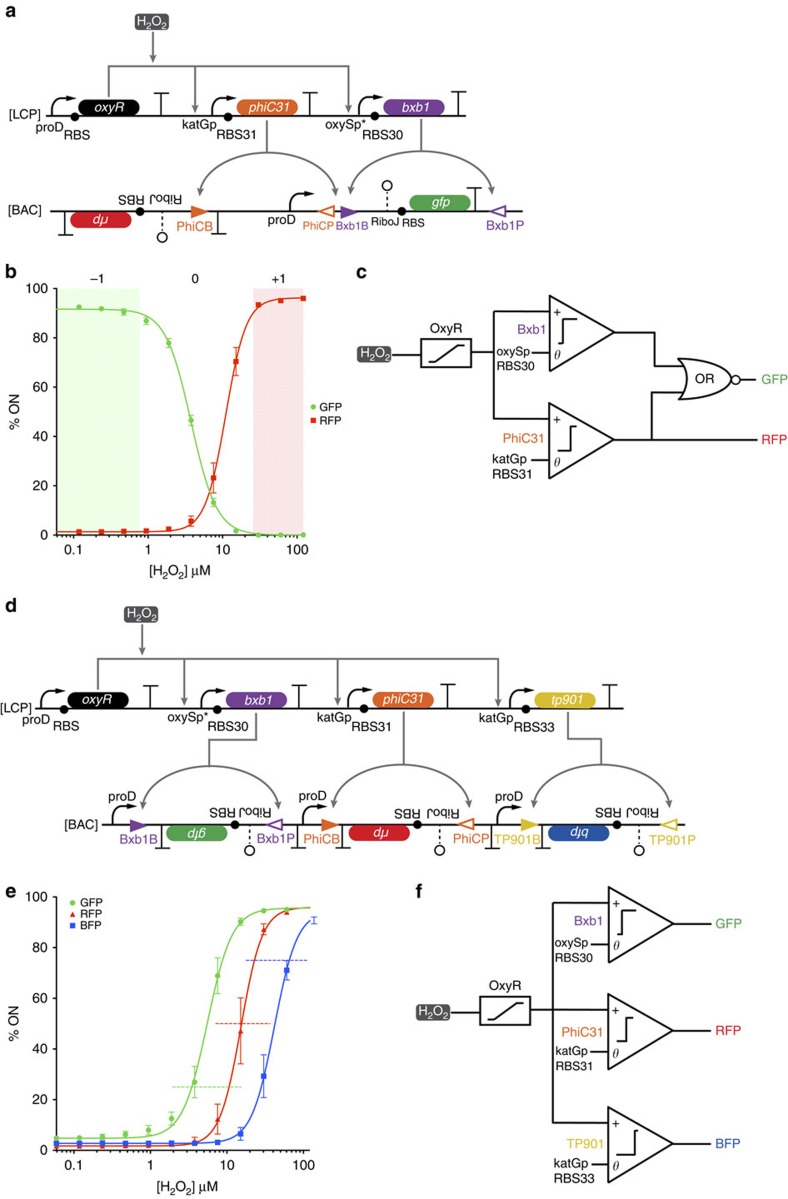
Multi-bit analogue-to-digital converters. (**a**) Ternary (three state) logic gene circuit. OxyR is constitutively expressed and activates transcription of *bxb1* and *phiC31* in response to increasing concentrations of H_2_O_2_. Bxb1 unpairs the *gfp* cassette from the proD promoter, and PhiC31 unpairs the proD promoter from the *gfp* cassette and pairs it with the *rfp* cassette. (**b**) The per cent of cells expressing GFP (green circle) and the per cent of cells expressing RFP (red square) were fit to sigmoidal functions (solid lines). The ‘−1' state (shaded green) is defined as >90% cells being GFP positive. The ‘+1' (shaded red) is defined as >90% of cells being RFP positive. The ‘0' state is when neither −1 nor +1 conditions are met. (**c**) Abstraction of ternary logic genetic circuit. H_2_O_2_ activates OxyR, which then activates expression of *bxb1* and *phiC31* depending on the thresholds set by the promoters and RBS of their respective circuits. GFP expression is repressed by *bxb1* OR *phiC31* activation, whereas RFP activation is dependent on *phiC31* activation. (**d**) 2-bit analogue-to-digital converter. OxyR is constitutively produced and activates transcription of *bxb1*, *phiC31* and *tp901* in response to increasing thresholds of H_2_O_2_. Bxb1, PhiC31 and TP901 invert *gfp*, *rfp* and *bfp*, respectively, to enable expression from three different upstream proD promoters. (**e**) The per cent of cells expressing GFP (green circle), RFP (red triangle) or BFP (blue square) was fit to sigmoidal functions (solid lines). The transition band for each circuit is demarcated by a horizontal dashed line of the same colour. Each transfer function had a similar relative input range. (**f**) Abstraction of 2-bit analog-to-digital converter. H_2_O_2_ activates OxyR, which then activates expression of *bxb1*, *phiC31* and *tp901* depending on the thresholds set by the promoters and RBS of their respective circuits. Bxb1, PhiC31 and TP901 then activate *gfp, rfp* and *bfp* expression, respectively. The errors (s.d.) are derived from flow cytometry experiments of three biological replicates, each of which involved *n*>30,000 gated events.

Comparators with different thresholds can be composed together to build more complex signal-processing circuits in living cells (Figs 2 and 3). For example, circuits that turn gene expression ON with increasing input concentrations (as in Fig. 1) can be considered high-pass circuits (since they allow high-concentration inputs to ‘pass' or be outputted). Next, to create low-pass circuits (which only allow low-concentration inputs to ‘pass'), we built a gene expression cassette that was ON in the basal state and used an inducible recombinase circuit to turn the output gene OFF by inverting the upstream promoter. Then, to create band-pass filters (Fig. 2), we combined a low-threshold high-pass circuit with either a medium- or high-threshold low-pass circuit (Fig. 2a,c), thus implementing the logic in Fig. 2e. The band-pass circuits switched GFP expression ON at low concentrations of H_2_O_2_ and switched GFP OFF at either medium or high concentrations of H_2_O_2_, depending on the threshold of the low-pass circuit (Fig. 2b,d; Supplementary Figs 7 and 8). The transfer function of each band-pass circuit could be predicted from straightforward addition of the transfer function of the high-pass circuit with the transfer function of the low-pass circuit that composed it (Supplementary Note 1). To determine the transfer functions of the high-pass and low-pass circuits, we measured GFP activation by the comparators using the same reporters for each recombinase as in Fig. 1 (Supplementary Figs 7 and 8). We defined the bandwidth of a band-pass filter as the relative input range over which the circuit switched from 50% ON to 50% OFF. The band-pass circuit composed of the low-threshold high pass and medium-threshold low pass had a relative input range of 3.16; the band-pass circuit composed of the low-threshold high pass and high-threshold low pass had a wider relative input range of 7.34. This circuit architecture can be adapted to create band-stop filters by making the low-threshold circuit a low pass and making the high-threshold circuit a high pass.

Higher-order signal-processing circuits can be designed to convert a single analogue input into multiple distinct outputs. For instance, we built ADCs^19^ that convert input H_2_O_2_ into the expression of multiple genes (Fig. 3). For example, we built a circuit that can be used to output a pair of signals that encode the information of a ternary output. The circuit measures input H_2_O_2_ concentration and converts it into three gene expression states that represent a confirmed low concentration (‘−1'), an intermediate concentration (‘0') or a confirmed high concentration (‘+1'). To construct this circuit (Fig. 3a,b), we altered the band-pass circuit in Fig. 2a such that *gfp* was initially expressed by the proD promoter, but would be shut off by Bxb1 production. We then added a copy of *rfp* that could be activated by inversion of the promoter by PhiC1 production. We defined the ‘−1' state as when >90% of cells were GFP positive and the ‘1' state as when >90% of cells were RFP positive. This resulted in three distinct gene expression states within the cells that were toggled at different H_2_O_2_ concentrations (Fig. 3c; Supplementary Fig. 9). In future work, the *rfp* and *gfp* outputs could be replaced by other genetic regulators that feed into downstream computing circuits. These types of circuits could be extended to implement ternary logic, to report inequalities (such as <, = and >), or to encode distinct outputs at low or high input levels to actuate downstream circuits.

We also built a circuit where multiple comparators with different thresholds were each used to drive expression of a different fluorophore, thus implementing an ADC (Fig. 3d,e). This circuit classified H_2_O_2_ concentrations into one of four gene expression states in each cell ([*gfp*, *rfp*, *bfp*]=000, 100, 110, 111) due to successive Bxb1, PhiC31 and TP901 expression with increasing H_2_O_2_, thereby encoding 2 bits of information (Fig. 3f; Supplementary Fig. 10). The relative input ranges of the threshold circuits (horizontal lines in Fig. 3f) were 7.79, 5.08 and 6.42 for *gfp*, *rfp* and *bfp* expression, respectively, demonstrating that the ADC operates similarly in each concentration range. The resolution of an electronic ADC is a measure of the number of output discrete values encoded across a continuous input voltage range^42^. We created an analogous figure of merit for genetic ADCs, where we measure the number of bits encoded across the ADC relative input range (Supplementary Note 1). We calculated this relative resolution for our ADC to be 3.84. Adding XOR (exclusive OR) and buffer gates downstream of the current GFP, RFP and BFP outputs should implement a canonical 2-bit ADC that generates a binary 2-bit output.

### A mixed-signal-processing gene circuit

**Figure 4:**
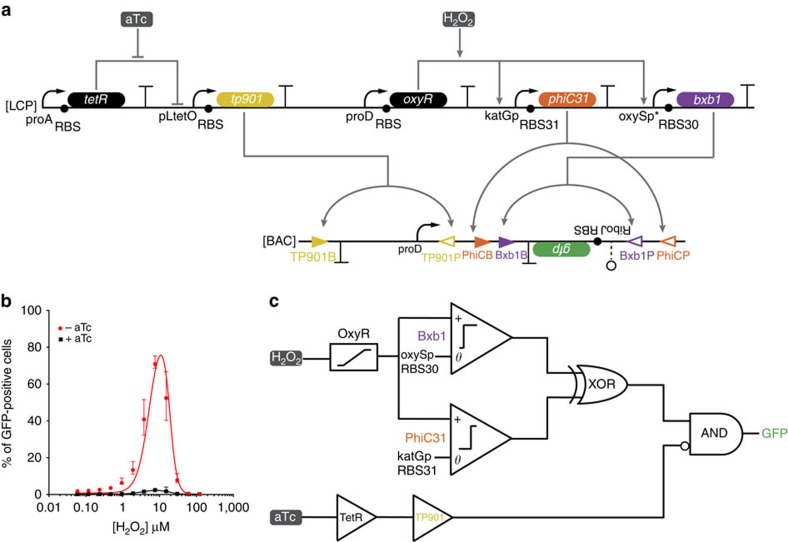
Mixed-signal computation and concentration-dependent logic. (**a**) Mixed-signal gene circuit. OxyR is constitutively produced and activates transcription of *bxb1* and *phiC31* at two different thresholds of H_2_O_2_. Both Bxb1 and PhiC31 can invert a *gfp* expression cassette. Bxb1-based flipping occurs at a lower H_2_O_2_ concentration than PhiC31-based flipping such that *gfp* is only in an upright orientation over an intermediate range of H_2_O_2_. Furthermore, TetR is constitutively produced and represses the pLtetO promoter; this repression is relieved by the presence of aTc. TP901 is expressed from the pLtetO promoter and inverts the proD promoter such that it cannot drive expression from an upright *gfp* cassette. The resulting circuit implements concentration-dependent logic with an output (GFP) that is ON only if an intermediate level of the input H_2_O_2_ is present and aTc is not present. (**b**) The per cent of cells expressing GFP at different concentrations of H_2_O_2_ in the presence (black square) and absence (red circle) of aTc. When aTc is absent, the circuit implements a band-pass response to H_2_O_2_, where the data are well fit by the same transfer function (red line) as the black line in Fig. 2b, *R*^2^=0.94. When aTc is present, the circuit is OFF. The black line is a straight line between each data point. (**c**) Abstraction of the mixed-signal gene circuit. H_2_O_2_ activates OxyR, which then activates expression of *bxb1* and *phiC31* depending upon the thresholds set by the promoters and RBS of their respective circuits. aTc activates expression of *tp901* via inactivation of TetR. GFP is expressed when either Bxb1 or PhiC31 are present AND NOT when TP901 is activated. The errors (s.d.) are derived from flow cytometry experiments of three biological replicates, each of which involved *n*>30,000 gated events.

Analogue-to-digital circuits can be further interfaced with digital circuits to form mixed-signal-processing circuits (Fig. 4). We built a variant of the band-pass circuit where the low-threshold comparator and medium-threshold comparator circuits both flip the directionality of *gfp.* This resulted in an analogue-to-digital circuit where only intermediate H_2_O_2_ levels enable GFP production, which is analogous to an XOR gate on H_2_O_2_ concentrations digitized using two different thresholds (Fig. 4a,b). In addition, we placed *tp901* under control of the TetR-repressed pLtetO promoter and constitutively expressed *tetR*, thereby making *tp901* digitally inducible by anhydrotetracycline (aTc)^39^. We then used *tp901* to control the direction of the promoter driving transcription of *gfp*. We assayed GFP levels at different H_2_O_2_ concentrations in the presence and absence of aTc, and found a majority of GFP-positive cells only at intermediate concentrations of H_2_O_2_ and when aTc was absent (Fig. 4b), thus implementing the concentration-dependent logic shown in Fig. 4c. Concentration-dependent logic could allow cells to carry out distinct activities at intermediate input levels, as opposed to extreme ones, and to encode a greater density of information into biological signals.

## Discussion

We have shown that cells can be engineered to implement synthetic computations that convert continuous information into discrete information. These computations rely on gene circuits that threshold and discretize signals from sensors, analogous to comparators in electronics. Our basic comparator design should be adaptable to other cellular contexts and for sensing inputs besides chemical concentration, such as light^13^ or contact^10^. There are other known ways to implement thresholding circuits^20^,^21^,^22^,^23^ and to dynamically alter thresholds^24^, suggesting that it would be possible to implement a negative input terminal analogous to that in electronic comparators, rather than the fixed threshold that we implemented here.

Our comparators can be composed together to build multi-threshold ADCs. In contrast to previously described genetic band-pass filters^25^,^26^,^27^,^28^, our band-pass filters convert continuous information into distinct gene expression states instead of altering continuous gene expression. Furthermore, the outputs from our ADCs can be integrated with other digital circuits (Fig. 4). Alternatively, multiple analogue signals could be integrated at the front end to calculate complex analogue functions^11^ before feeding the output(s) into downstream ADCs. We have engineered the outputs of our circuits to be Boolean (Figs. 2 and 4), ternary (Fig. 3a–c) or multi-state digital (Fig. 3d–f). It may be possible to further increase ADC resolution by increasing the number of comparators across the same range of H_2_O_2_ or by adding comparators that can respond to lower or higher concentrations of H_2_O_2_.

There are a number of potential challenges involved in scaling mixed-signal gene circuits. First, it is important that comparators do not substantially affect cell growth. We found that the number of plasmids on which comparator circuits are encoded impacted cell growth more than the number of comparator circuits (Supplementary Figs 13 and 14). Thus, to scale mixed-signal computation, it will be important to decrease the number of episomal DNA constructs, for example, by moving comparators to the chromosome. Furthermore, to increase ADC resolution, comparators will need to have sharper thresholds at the population level (that is, more consistency in the behaviour of each cell around the threshold point). We surmise that this may be possible by implementing negative feedback in the analogue sensor circuit, which can reduce population-level heterogeneity^43^. Screening large promoter/RBS/recombinase libraries could enable the identification of circuits that implement various thresholds on a given analogue input. Using novel orthogonal recombinases could aid in the scaling of mixed-signal gene circuits^44^. For certain analogue inputs, it may also be necessary to implement a graded positive-feedback^11^ or negative-feedback loop^45^ to enable wide input dynamic range activation of the sensor transcription factor.

ADCs are the complement of digital-to-analogue converters (DACs): ADCs convert an analogue input signal into discrete output signals, whereas DACs convert discrete input signals into analogue output signals (Supplementary Fig. 12a–c). For example, DACs that we previously implemented in living cells accepted two digital inputs and produced four different gene expression levels as outputs depending on the specific combination of inputs (Supplementary Fig. 12d)^36^. Here we built ADCs that translate a single analogue input in the form of inducer concentration to multiple discrete outputs, represented by triggering the expression of different genes (Supplementary Fig. 12e).

These mixed-signal circuits constitute a first step towards advanced analogue–digital hybrid computational approaches. For instance, to implement an artificial neural network circuit, multiple analogue inputs could be fed into the promoter controlling recombinase expression, and the weights of the analogue inputs could be tuned via their binding affinity to the promoter. Linking these artificial circuits together could allow the creation of artificial neuronal networks in living cells. The comparators could also be used in a hybrid automaton if they were integrated with state machines, wherein states switch based on analogue thresholds. In addition, the ternary logic circuit (Fig. 3a) could be used to implement fuzzy logic by converting the ‘0' state into the expression of a third gene.

We envision that mixed-signal processing will enable a wide range of industrial^46^, diagnostic and therapeutic applications using engineered cells^47^,^48^. For example, cells could be designed to produce quorum-sensing signals that trigger multiple distinct production pathways as the quorum-sensing molecules accumulate in a bioreactor. The first phase could be focused on biomass accumulation, the second phase dedicated to secreting the desired product, such as a biologic protein drug fused to a secretion tag, and the third committed to secreting product-modifying enzymes, such as a protease to separate the secretion tag from the active drug. Such behaviour could be programmed with an ADC that senses the concentration of an accumulating quorum-sensing molecule as an input and triggers successive circuits with higher concentrations, similar to the system shown in Fig. 3d,e. As a first step towards such industrial applications, we scaled up the operational volume of the ADC circuit by 100 × and found the circuit functioned, albeit with shifted thresholds (Supplementary Fig. 15).

In addition, cells could be designed to detect continuous quantities of multiple biomarkers, integrate these signals to diagnose disease conditions and produce reporter output(s) for non-invasive biosensing applications. For instance, probiotic or commensal^49^ bacteria could be engineered to sense the concentration of multiple biomarkers for inflammatory bowel disease (for example, reactive oxygen species, nitric oxide and blood), discretize the magnitude of each of these analogue signals using ADCs with a range of thresholds, integrate the resulting information with Boolean logic (for example, a multi-input AND gate) to decide whether a disease flare-up is occurring and how severe it is, and produce discrete reporters that can be detected outside of the body. Reporting on disease states and severity with digitized outputs (for example, different fluorescent or colorimetric reporters) could be more robust than analogue outputs (for example, a single fluorescent reporter expressed at different levels), since the latter is more susceptible to noise. Our ADCs could also be used as peak detectors due to the inherent memory feature of recombinase-based switches. For instance, probiotic bacteria could be engineered to remember the maximum concentration of a biomarker that they detected while passing through the intestine. Similar circuits could be used to create environmental sensors that sense and record maximum pollutant levels^50^.

Mixed-signal circuits could also be useful for engineering cell therapies whose therapeutic outputs are regulated by quantitative levels of disease biomarkers. For example, mammalian gene circuits could be designed such that blood glucose levels below the normal region (‘−1' in a ternary logic system) would switch on glucagon secretion, blood glucose levels in the desired region (‘0' in a ternary logic system) would result in no hormone secretion and blood glucose levels above the normal region (‘1' in a ternary logic system) would trigger insulin secretion. The ability to trigger distinct outputs in response to different conditions could enable new ‘homeostatic' therapies. As a first step towards such applications, we tested the temporal response of the ternary logic circuit (Fig. 3a) and found that the cells likely induce recombination well before a change in fluorescent protein gene expression is observed (Supplementary Fig. 16). Such applications would benefit from resettable mixed-signal circuits, which could be implemented using transcriptional regulators, rather than the permanent-memory mixed-signal circuits described here.

In summary, mixed-signal gene circuits merge analogue and digital signal processing to enable both continuous information sensing and robust multi-signal integration, and computing in living cells. Ultimately, we expect that this hybrid analogue–digital computational paradigm will allow synthetic biological systems to begin to approach the nuanced complexities found in natural biological systems^14^,^15^,^18^,^49^,^50^,^51^,^52^,^53^,^54^,^55^,^56^,^57^,^58^,^59^,^60^.

## Methods

### Strains and plasmids

All plasmids were constructed using PCR and Gibson assembly starting from DNA sources as referenced in Supplementary Table 2 or from gBlocks manufactured by IDT. *E. coli* EPI300 (*F*^−^ *mcrA Δ(mrr-hsdRMS-mcrBC) Φ80dlacZΔM15 ΔlacX74 recA1 endA1 araD139 Δ(ara, leu)7697 galU galK λ*^−^ *rpsL (Str*^*R*^*) nupG trfA dhfr*) was used for all experiments. Parts and plasmids used in this study are detailed in Supplementary Fig. 17, Supplementary Tables 1 and 2, and Supplementary Data 1 and 2. Plasmid sequences and plasmid DNA can be obtained at Addgene under Addgene ID numbers 78211–78229.

### Circuit characterization

Plasmids were transformed into chemically competent *E. coli* EPI300, plated on LB medium with appropriate antibiotics and grown overnight at 37 °C. Antibiotic concentrations were carbenicillin (50 μg ml^−1^), kanamycin (30 μg ml^−1^) and chloramphenicol (25 μg ml^−1^). The next day, single colonies were inoculated into Teknova Hi-Def Azure Media with appropriate antibiotics and 0.2% glucose, and incubated shaking aerobically for 16–18 h at 37 °C. Cultures were then diluted 2,500 × into fresh Hi-Def Azure Media with appropriate antibiotics and 0.2% glucose, and shaken for 20 min aerobically at 37 °C. After 20 min, 200 μl of culture was transferred to a 96-well plate, and H_2_O_2_ (Sigma–Aldrich H1009-100ML) was added at appropriate concentration via serial dilution. For the experiment in Fig. 4, aTc (Cayman Chemical 10009542) was added to a final concentration of 75 ng ml^−1^. Plates were incubated aerobically with shaking for 20 h at 30 °C for all experiments except those in Supplementary Fig. 1, in which plates were incubated for 3 h. After incubation, the optical densities of cultures were measured at 600 nm in a plate reader. For experiments in Supplementary Figs 3–5, cells were then assayed on the flow cytometer. For all other experiments (Figs 1, 2, 3, 4; Supplementary Figs 6–11), cells were washed with PBS, diluted 8 × into fresh Hi-Def Azure Media with appropriate antibiotics, 0.4% glycerol and 1 × CopyControl Induction Solution (Epicentre), and incubated shaking aerobically for a further 10 h at 30 °C. After this incubation, the optical densities of cultures were measured at 600 nm in a plate reader. For all flow cytometer experiments, cells were diluted into ice-cold 1 × PBS to an optical density at 600 nm of <0.02 and assayed on a BD LSRFortessa using the high-throughput sampler. At least 30,000 gated events were recorded. GFP expression was measured via the fluorescein isothiocyanate channel, RFP expression was measured via the TexasRed channel and BFP expression was measured via the Pacific Blue channel. FCS files were exported and processed in FlowJo software. Events were gated for live *E. coli* via forward scatter area and side scatter area, and then analysed as in Supplementary Note 1. The y axis on the flow cytometry histograms is normalized to the mode for each sample. At least three biological replicates were conducted for each experiment.

### Data availability

Plasmids described in this study have been deposited into the Addgene repository under Addgene ID numbers 78211–78229. The authors declare that all other data supporting the findings of this study are available within the article and its Supplementary Information files or are available from the corresponding author upon request.

## Additional information

**How to cite this article**: Rubens, J. R. *et al*. Synthetic mixed-signal computation in living cells. *Nat. Commun.* 7:11658 doi: 10.1038/ncomms11658 (2016).

## Supplementary Material

Supplementary Information Supplementary Figures 1-17, Supplementary Tables 1-3, Supplementary Note 1 and Supplementary References

Supplementary Data 1 Part sequences

Supplementary Data 2 Plasmid sequences
