# Supplementary material for: Synthetic mixed-signal computation in living cells
Source: Nat Commun. 2016 Jun 3;7:11658. doi: 10.1038/ncomms11658 (PMC4895730; doi:10.1038/ncomms11658)
Supplement: Supplementary Information — Supplementary Figures 1-17, Supplementary Tables 1-3, Supplementary Note 1 and Supplementary References [file ncomms11658-s1.pdf]

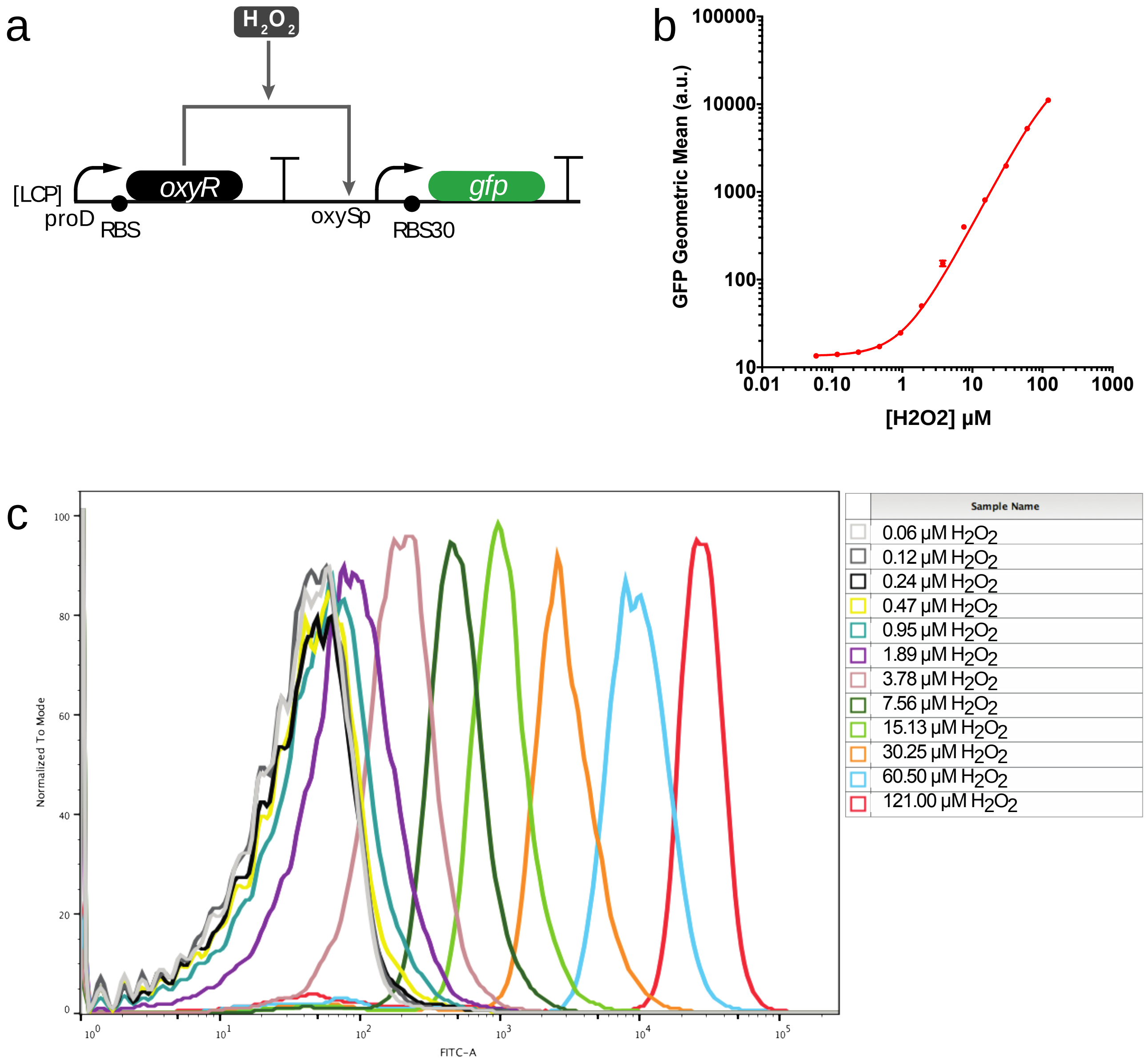

Supplementary Figure 1 | Analog H_2O_2–sensor.

a. OxyR is constitutively expressed from a low-copy plasmid (LCP) and activates transcription of *gfp* from the oxySp promoter on the same LCP in response to H_2O_2.

b. The geometric mean of GFP expression at different concentrations of H_2O_2 was measured three hours after induction. The line is a Hill function fit to the data. The errors (standard error of the mean) are derived from flow cytometry experiments of three biological replicates, each of which involved $n >$ 30,000 gated events.

c. Representative flow cytometry histograms for the analog circuit shown in **Supplementary Fig. 1a** at different H_2O_2 concentrations. GFP is measured with FITC. GFP expression is continuously activated with increasing H_2O_2 over at least two orders of magnitude of the input.

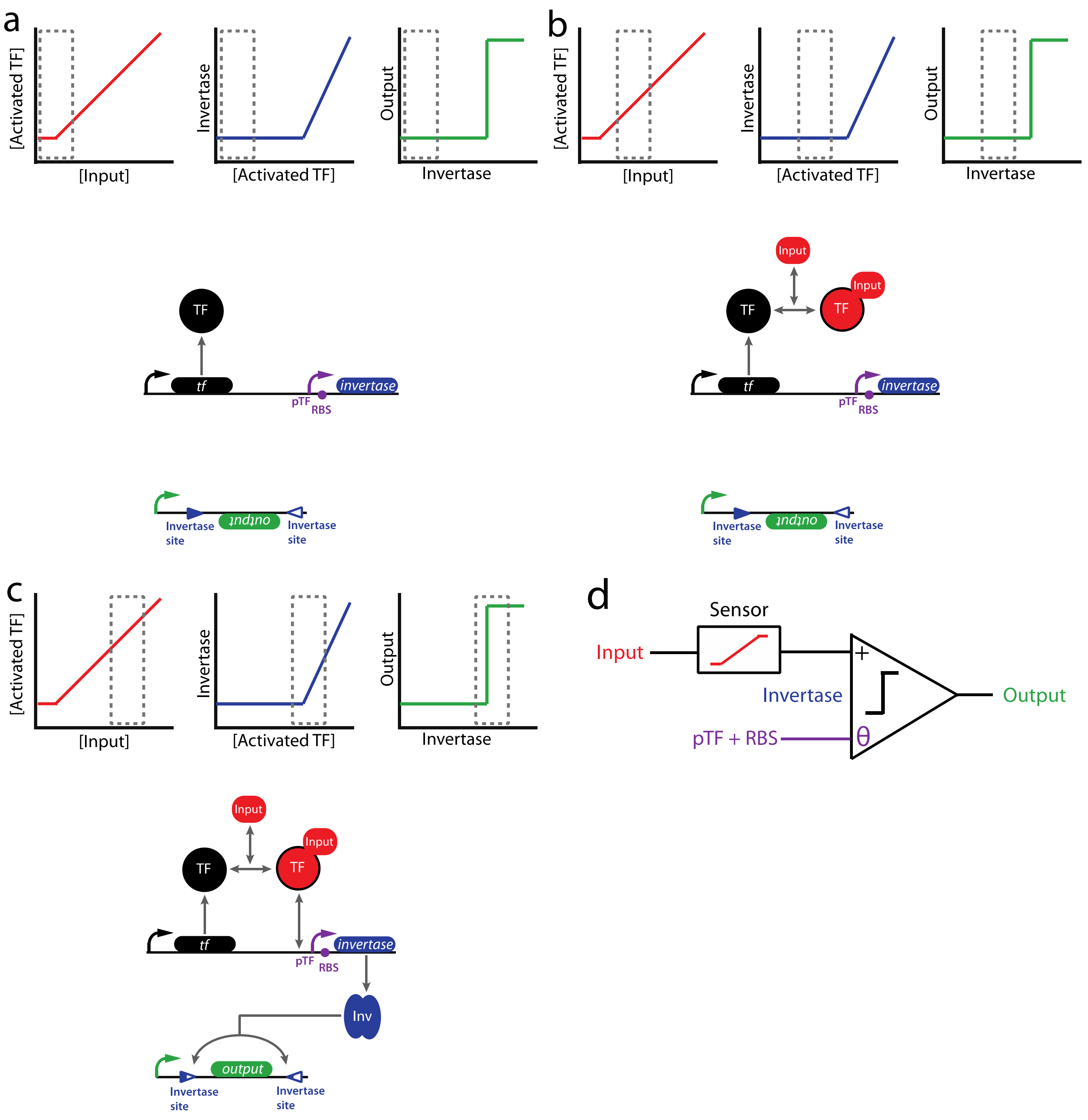

Supplementary Figure 2 | Comparator overview.

a. At low input concentrations, the transcription factor gene (*tf*) is constitutively expressed, but the TF is not activated to a significant level. Consequently, the *invertase* gene is not expressed.

b. At medium input concentrations, the TF is activated (red TF bound to Input), but it is below the concentration needed for significant expression of the *invertase* gene.

c. At high input concentrations, the concentration of activated TF is sufficient to activate expression of the *invertase* from a specific promoter (pTF). The Invertase (Inv) binds to the invertase sites (triangles) and inverts the DNA between the sites. This results in the expression of the *output* gene by the upstream promoter (green arrow), leading to output expression.

d. A genetic comparator abstraction. It is composed of the threshold module (purple), the digitization module (blue) and the output module (green). An input activates a sensor (such as a transcription factor), and this transcription factor activates the expression of an invertase at an input threshold (θ) defined by the affinity of the invertase promoter for the activated transcription factor and by the translation strength of the invertase as defined by its RBS. When the invertase is expressed, the output is switched ON.

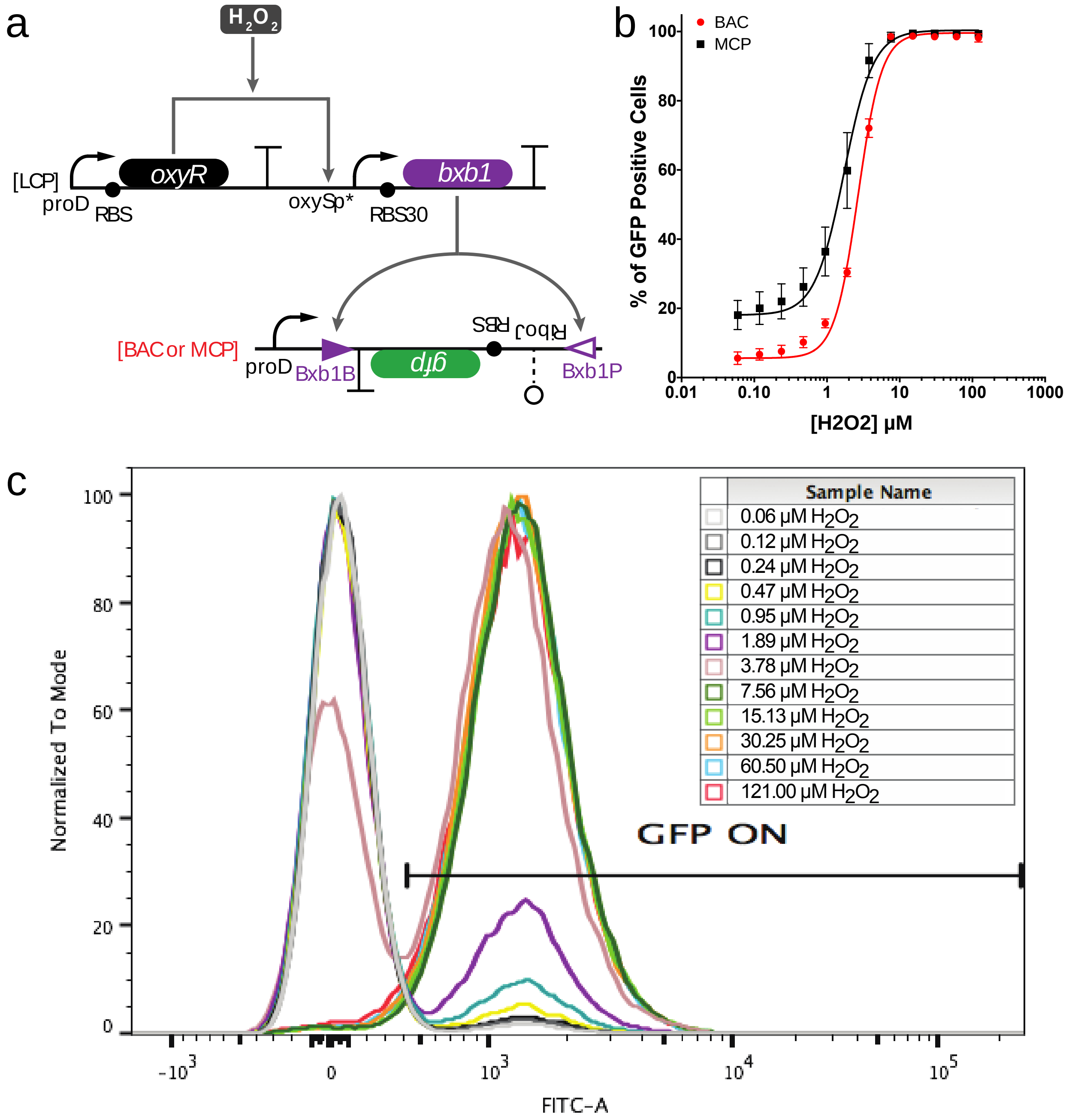
a
H_2O_2
[LCP]
proD
RBS
oxyR
oxySp*
RBS30
bxb1
[BAC or MCP]
proD
Bxb1B
gfp
RBS
Rbo1
Bxb1P
b
BAC
MCP
% of GFP Positive Cells
[H2O2] μM
c
Normalized To Mode
Sample Name
0.06 μM H_2O_2
0.12 μM H_2O_2
0.24 μM H_2O_2
0.47 μM H_2O_2
0.95 μM H_2O_2
1.89 μM H_2O_2
3.78 μM H_2O_2
7.56 μM H_2O_2
15.13 μM H_2O_2
30.25 μM H_2O_2
60.50 μM H_2O_2
121.00 μM H_2O_2
GFP ON
FITC-A

d

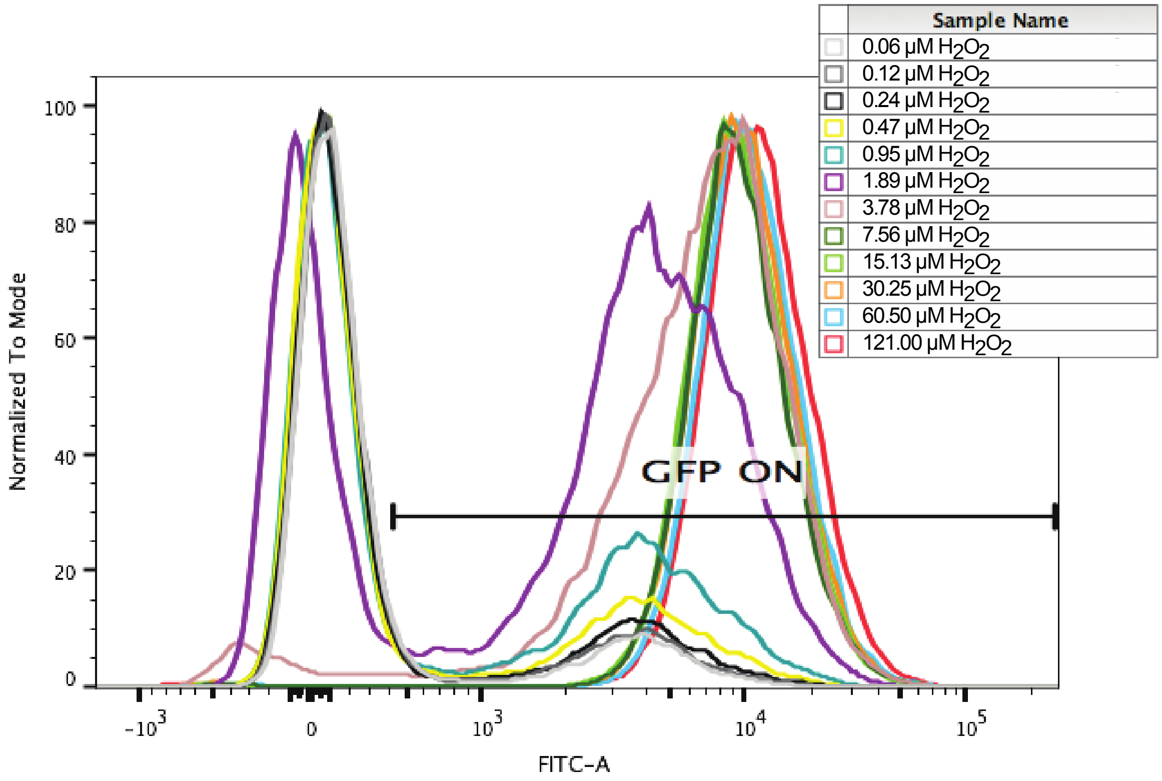
Sample Name
0.06 µM H2O2
0.12 µM H2O2
0.24 µM H2O2
0.47 µM H2O2
0.95 µM H2O2
1.89 µM H2O2
3.78 µM H2O2
7.56 µM H2O2
15.13 µM H2O2
30.25 µM H2O2
60.50 µM H2O2
121.00 µM H2O2
Normalized To Mode
GFP ON
FITC-A

e

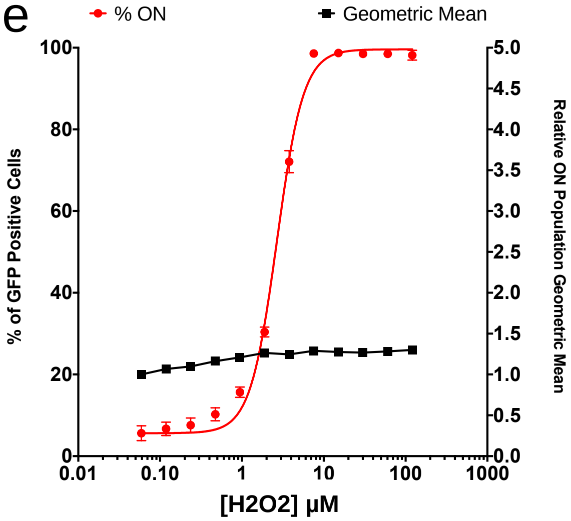
% ON
Geometric Mean
% of GFP Positive Cells
Relative ON Population Geometric Mean
[H2O2] µM

f

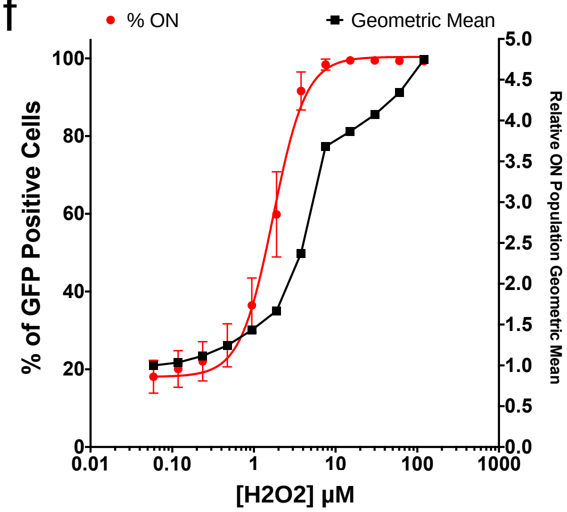
% ON
Geometric Mean
% of GFP Positive Cells
Relative ON Population Geometric Mean
[H2O2] µM

g

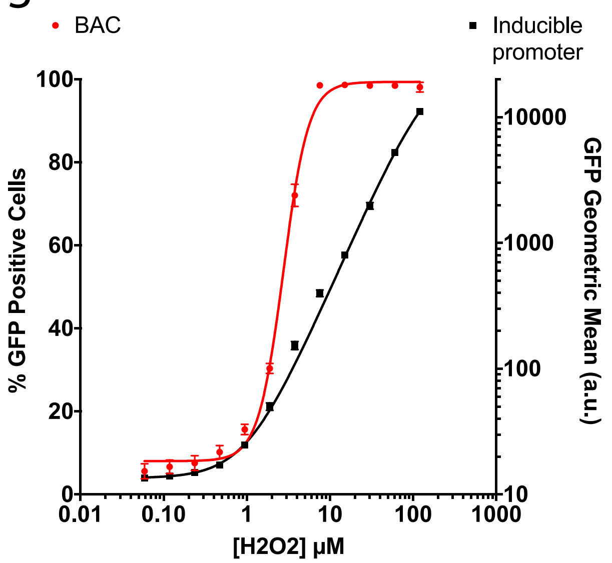

Supplementary Figure 3 | Digitization of an analog input by inverting target DNA on a medium-copy plasmid (MCP) versus a bacterial artificial chromosome (BAC).

a. OxyR is constitutively expressed from a LCP and activates transcription of *bxb1* from the oxySp* promoter on the same LCP in response to H_2O_2. Bxb1 inverts the *gfp* expression construct on a BAC or MCP, turning on *gfp* expression by pairing it with an upstream proD promoter.

b. The percent of GFP positive cells at different H_2O_2 concentrations as measured by flow cytometry. The BAC (red circles) and MCP (black squares) have similar transfer functions. However, the MCP exhibits a higher basal level of cells that are GFP positive. The errors (standard deviation) are derived from flow cytometry experiments of three biological replicates, each of which involved $n > 30{,}000$ gated events.

c. Representative flow cytometry histograms for the BAC circuit shown in **Supplementary Fig. 3a** at different H_2O_2 concentrations. GFP is measured with FITC. The GFP-positive cells maintain a consistent level of GFP fluorescence even with increased H_2O_2, indicating a homogeneous population.

d. Representative flow cytometry histograms for the MCP circuit shown in **Supplementary Fig 3a** at different H_2O_2 concentrations. The GFP-positive cells demonstrate increasing levels of GFP fluorescence with increased H_2O_2, indicating that there are multiple heterogeneous subpopulations.

e. The % of GFP positive cells vs. concentration of H_2O_2 (red circles) for the BAC circuit from **Supplementary Fig. 3a** is fit to a transfer function and plotted on the left y-axis. The geometric mean of the GFP positive cells in **Supplementary Fig. 3c** relative to the minimum geometric mean of the GFP positive cells in the same experiment vs. concentration of H_2O_2 (black squares) is plotted on the right y-axis and adjacent points are directly connected by straight lines (black line). The geometric mean does not considerably increase with H_2O_2, indicating that GFP positive cells in **Supplementary Fig. 3c** constitute one population even at different levels of the input.

f. The % of GFP positive cells vs. concentration of H_2O_2 (red circles) for the MCP circuit from **Supplementary Fig. 3a** is fit to a transfer function and plotted on the left y-axis. The geometric mean of the GFP positive cells in **Supplementary Fig. 3d** relative to the minimum geometric mean of the GFP

positive cells in the same experiment vs. concentration of H_2O_2 (black squares) is plotted on the right y-axis and adjacent points are directly connected by straight lines (black line). The geometric mean increases considerably with H_2O_2, indicating that GFP positive cells in **Supplementary Fig. 3d** take on multiple populations with different H_2O_2 levels.

g. Digitization of the input by the comparator circuit. The percent of GFP positive cells at different H_2O_2 concentrations as measured by flow cytometry for the BAC comparator circuit (red circles) is plotted on the left axis (same data as black squares in **Supplementary Fig. 3b**). For comparison, we have also plotted the geometric mean of GFP expression at different concentrations of H_2O_2 (black squares) on the right axis (same data as red circles in **Supplementary Fig. 1b**). The five-highest tested concentrations of H_2O_2 continuously increase GFP expression from the inducible promoter but do not increase the percent of GFP positive cells from a comparator.

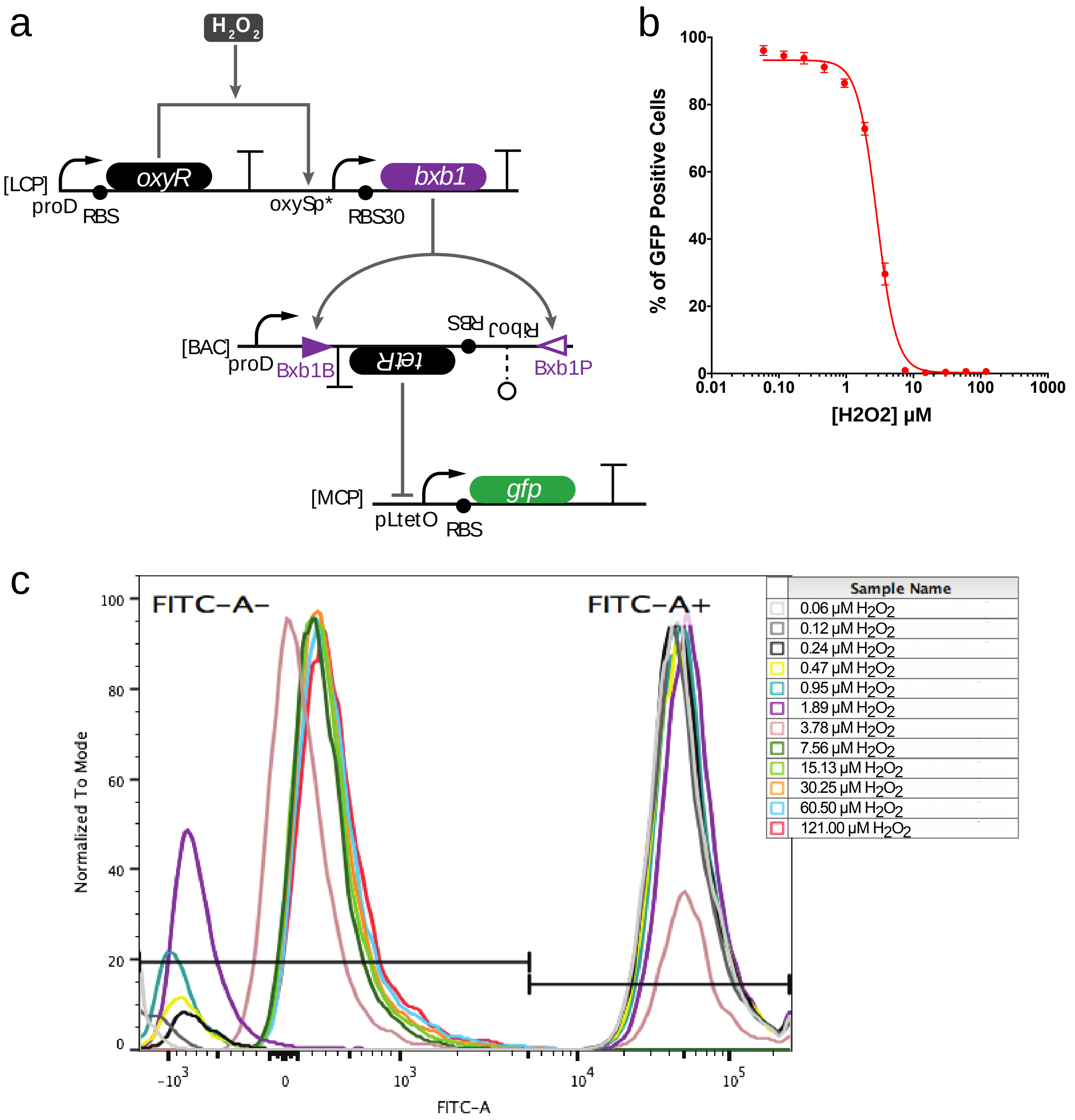

Supplementary Figure 4 | Feedforward cascade involving a recombinase-invertible trans-acting transcriptional regulator on a BAC.

a. OxyR is constitutively expressed from a LCP and activates transcription of *bxb1* from the oxySp* promoter on the same LCP in response to H_2O_2. Bxb1 inverts the *tetR* expression cassette on a BAC, turning on TetR expression by pairing it with the proD promoter. TetR represses *gfp* expression from pLtetO on a MCP.

b. The percent of GFP positive cells at different H_2O_2 concentrations as measured by flow cytometry. The transfer function has a narrow switching range. The errors (standard deviation) are derived from flow cytometry experiments of three biological replicates, each of which involved $n > 30{,}000$ gated events.

c. Representative flow cytometry histograms for the circuit shown at in **Supplementary Fig. 4a** at different H_2O_2 concentrations. GFP is measured with FITC. The GFP-positive cells fall into one population.

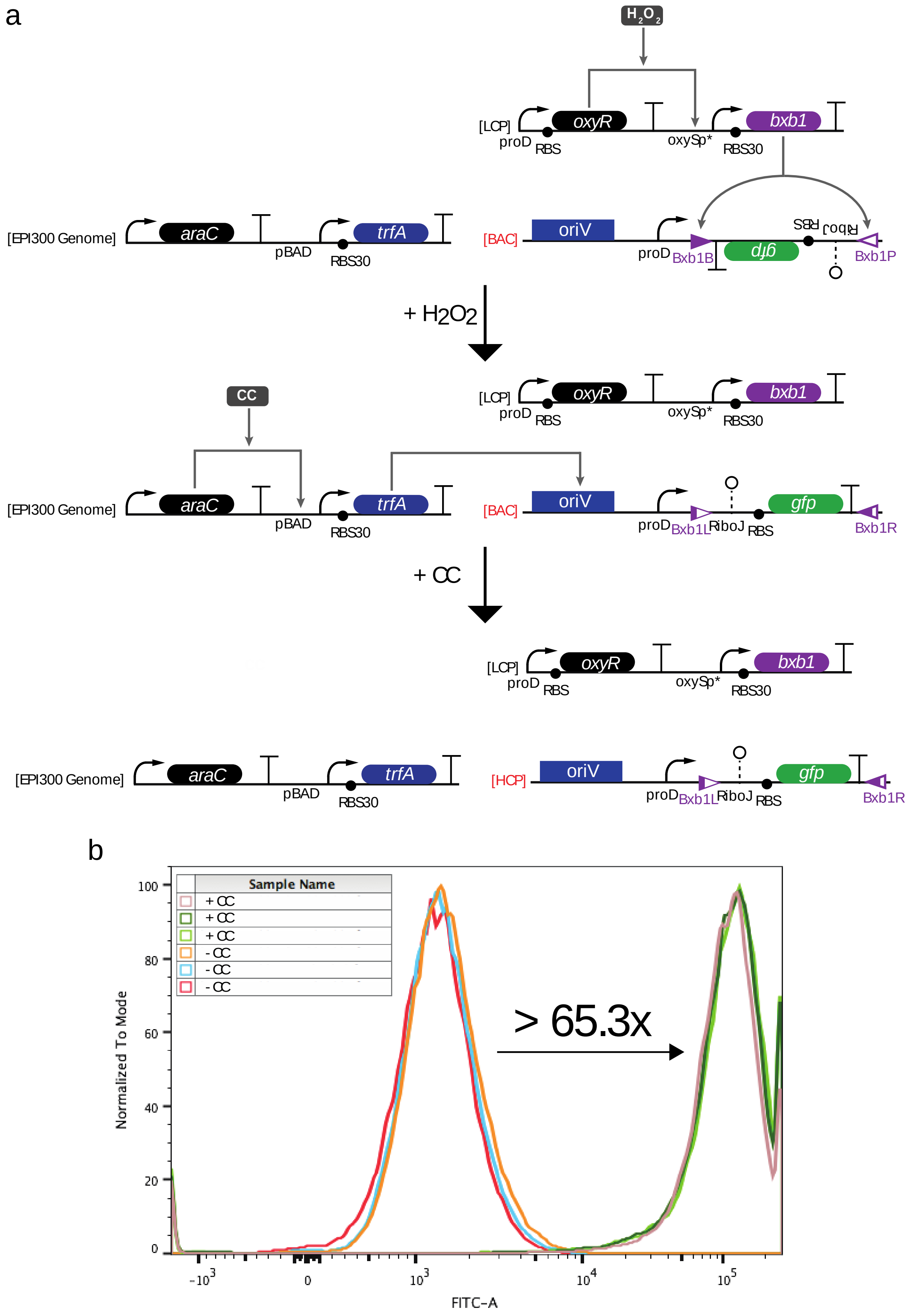

a
H2O2
[LCP]
proD
RBS
oxyR
oxySp*
RBS30
bxb1
[EPI300 Genome]
araC
pBAD
RBS30
trfA
[BAC]
oriV
proD
Bxb1B
gfp
RiboJ
RBS
Bxb1P
+ H2O2
CC
Bxb1L
Bxb1R
+ CC
[HCP]
b
Sample Name
+ CC
- CC
> 65.3x
Normalized To Mode
FITC-A

c

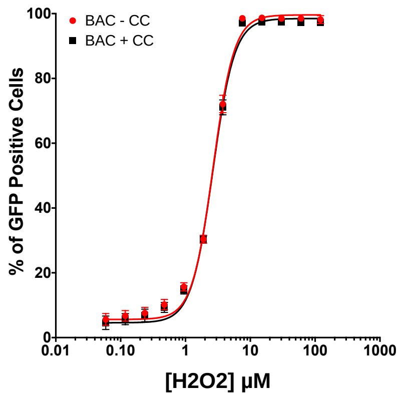
BAC - CC
BAC + CC
% of GFP Positive Cells
[H2O2] µM
0.01 0.10 1 10 100 1000
0 20 40 60 80 100

d

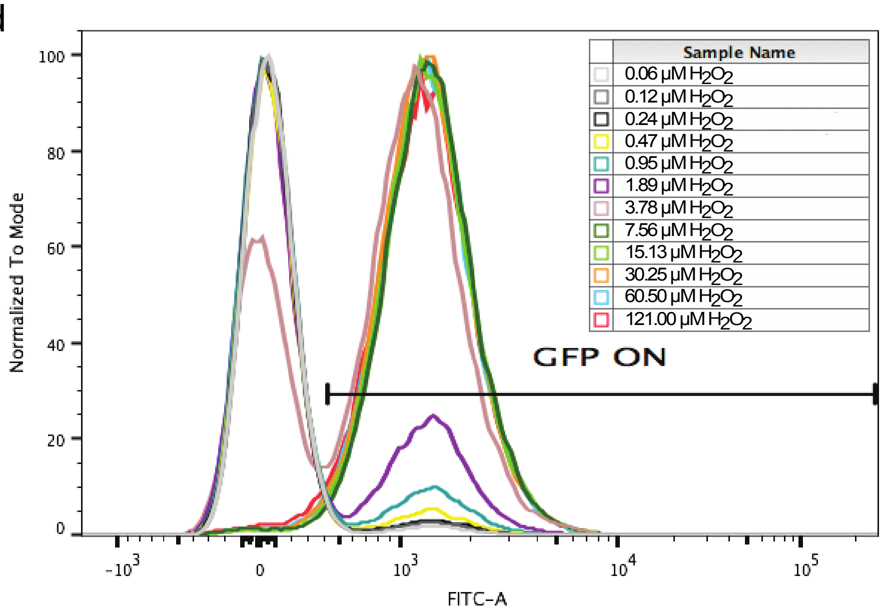
Sample Name
0.06 µM H2O2
0.12 µM H2O2
0.24 µM H2O2
0.47 µM H2O2
0.95 µM H2O2
1.89 µM H2O2
3.78 µM H2O2
7.56 µM H2O2
15.13 µM H2O2
30.25 µM H2O2
60.50 µM H2O2
121.00 µM H2O2
GFP ON
Normalized To Mode
FITC-A

e

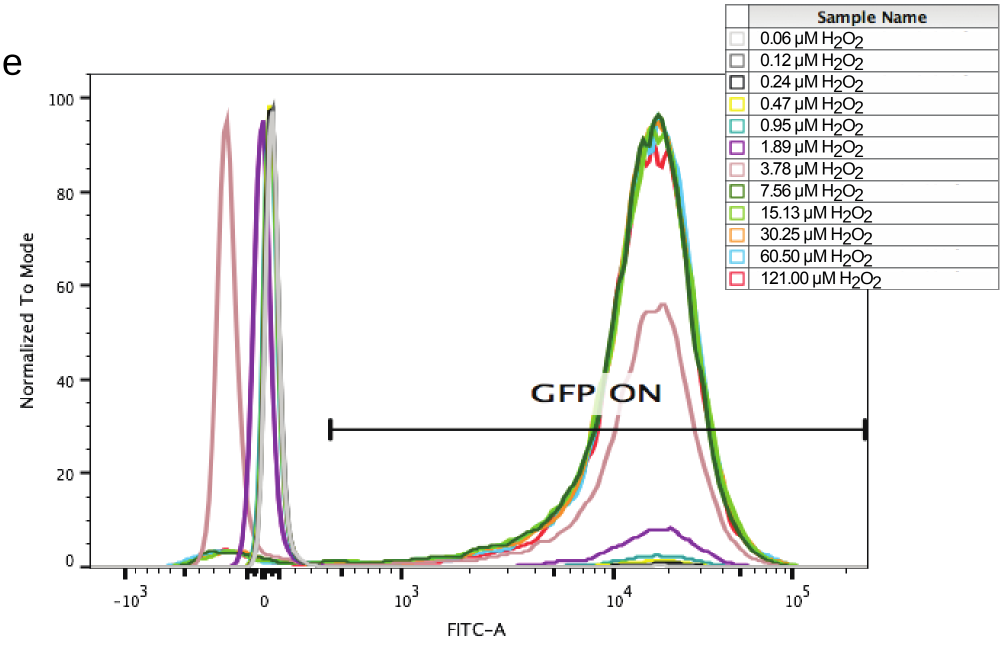
Sample Name
0.06 µM H2O2
0.12 µM H2O2
0.24 µM H2O2
0.47 µM H2O2
0.95 µM H2O2
1.89 µM H2O2
3.78 µM H2O2
7.56 µM H2O2
15.13 µM H2O2
30.25 µM H2O2
60.50 µM H2O2
121.00 µM H2O2
GFP ON
Normalized To Mode
FITC-A

Supplementary Figure 5 | Amplifying BAC output with Copy Control. We used a BAC that also has an origin of replication that can be activated by a plasmid replication factor integrated into the genome of EPI300 *E. coli* under inducible control by Copy Control (CC) reagent[38].

a. Cells were first incubated with different concentrations of H_2O_2 to induce GFP expression. Cells were then washed and diluted into fresh media with CC. CC induces *trfA* expression from the pBAD promoter via activation of AraC, which are both expressed from the EPI300 chromosome. TrfA amplifies the BAC from 1-2 copies per cell to a high copy plasmid (HCP) at ∼100 copies per cell[38].

b. Flow cytometry histograms for GFP expression from the BAC with CC (purple, dark green, light green) and without CC (orange, blue, red) at 121 µM H_2O_2. CC amplifies GFP expression at least 63.5x as measured by the geometric means of the populations.

c. The transfer functions for the BAC with CC (black line, black squares) and without CC (red line, red circles) are nearly identical. The errors (standard deviation) are derived from flow cytometry experiments of three biological replicates, each of which involved $n > 30{,}000$ gated events.

d. Representative flow cytometry histograms for the BAC at different concentrations of H_2O_2 without CC for the data in **Supplementary Fig. 5c**.

e. Representative flow cytometry histograms for the BAC at different concentrations of H_2O_2 with CC for the data in **Supplementary Fig. 5c**.

Note that the experiments in figures **Supplementary Fig. 5b** and **5d** were measured with the same FITC voltage on the flow cytometer, and **Supplementary Fig. 5e** was measured with a different, lower FITC voltage on the flow cytometer because GFP expression from the BAC+CC was greater than the measurable fluorescence at the higher FITC voltage (as can be seen in the +CC data in **Supplementary Fig. 5b**).

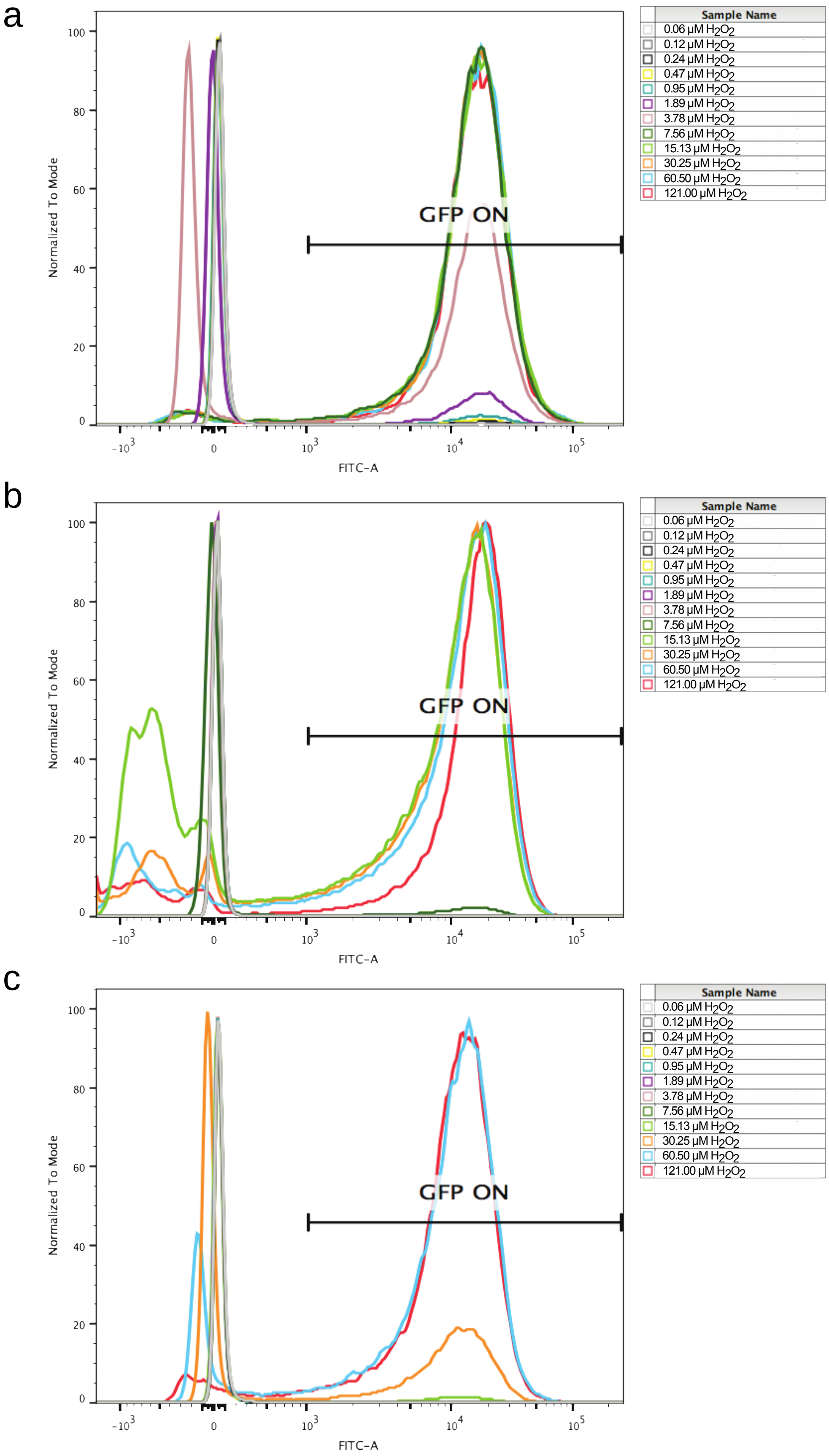
a
b
c
Normalized To Mode
FITC-A
GFP ON
100
80
60
40
20
0
-10^3
0
10^3
10^4
10^5
Sample Name
0.06 μM H_2O_2
0.12 μM H_2O_2
0.24 μM H_2O_2
0.47 μM H_2O_2
0.95 μM H_2O_2
1.89 μM H_2O_2
3.78 μM H_2O_2
7.56 μM H_2O_2
15.13 μM H_2O_2
30.25 μM H_2O_2
60.50 μM H_2O_2
121.00 μM H_2O_2

Supplementary Figure 6 | Flow cytometry histograms for comparators with different activation thresholds (Fig. 1).

a. Representative flow cytometry histograms for GFP expression for the low threshold circuit shown in **Fig. 1a** with oxySp* and RBS30, which correspond to the red diamonds and red line in **Fig. 1b**.

b. Representative flow cytometry histograms for GFP expression for the medium threshold circuit shown in **Fig. 1c** with katGp and RBS31, which correspond to the red triangles and red line in **Fig. 1d**.

c. Representative flow cytometry histograms for GFP expression for the high threshold circuit shown in **Fig. 1e** with katGp and RBS33, which correspond to the red diamonds and red line in **Fig. 1f**.

a

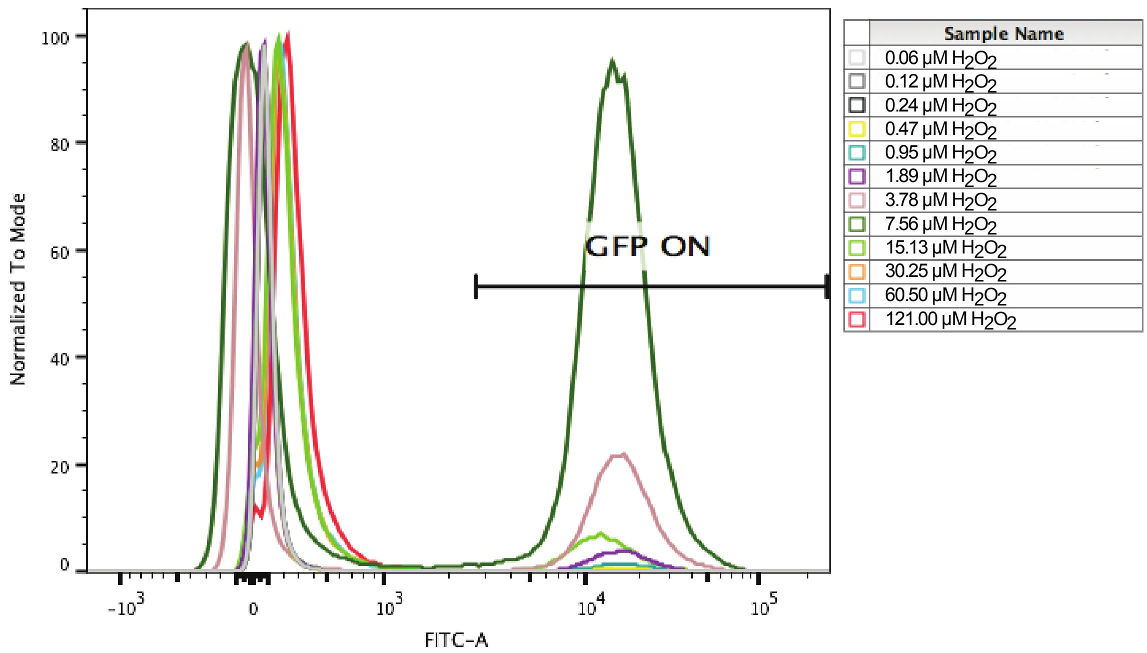

Normalized To Mode
100
80
60
40
20
0
-10^3
0
10^3
10^4
10^5
FITC-A
GFP ON
Sample Name
0.06 μM H2O2
0.12 μM H2O2
0.24 μM H2O2
0.47 μM H2O2
0.95 μM H2O2
1.89 μM H2O2
3.78 μM H2O2
7.56 μM H2O2
15.13 μM H2O2
30.25 μM H2O2
60.50 μM H2O2
121.00 μM H2O2

b

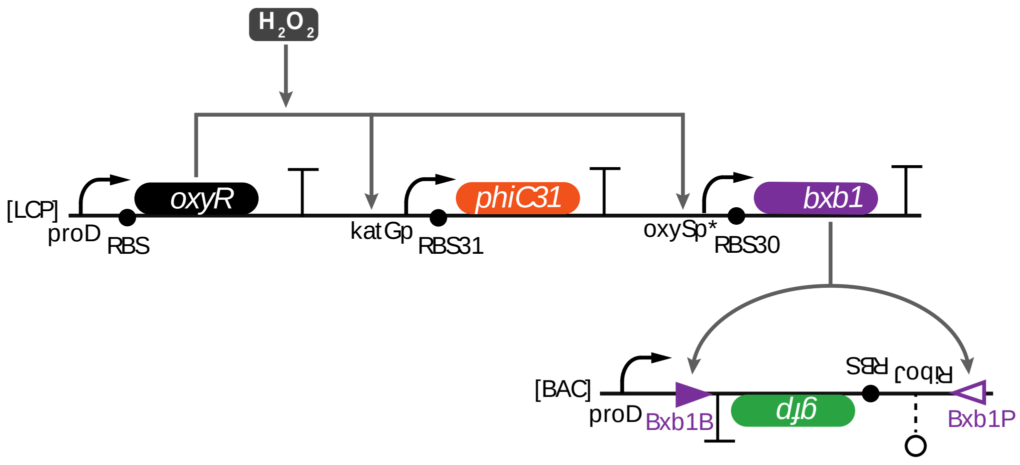

H2O2
[LCP]
proD
RBS
oxyR
katGp
RBS31
phiC31
oxySp*
RBS30
bxb1
[BAC]
proD
Bxb1B
gfp
RBS
RiboJ
Bxb1P

c

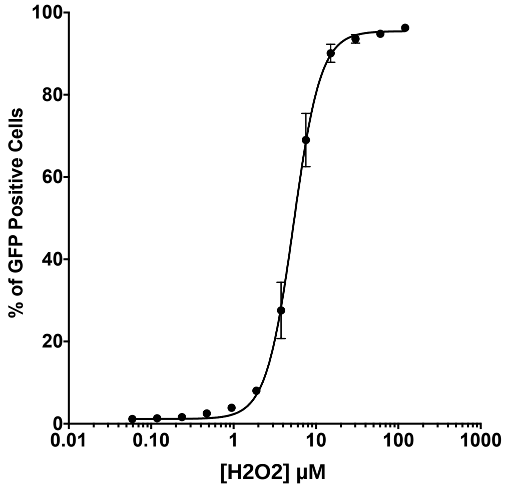

% of GFP Positive Cells
0
20
40
60
80
100
0.01
0.10
1
10
100
1000
[H2O2] μM

d

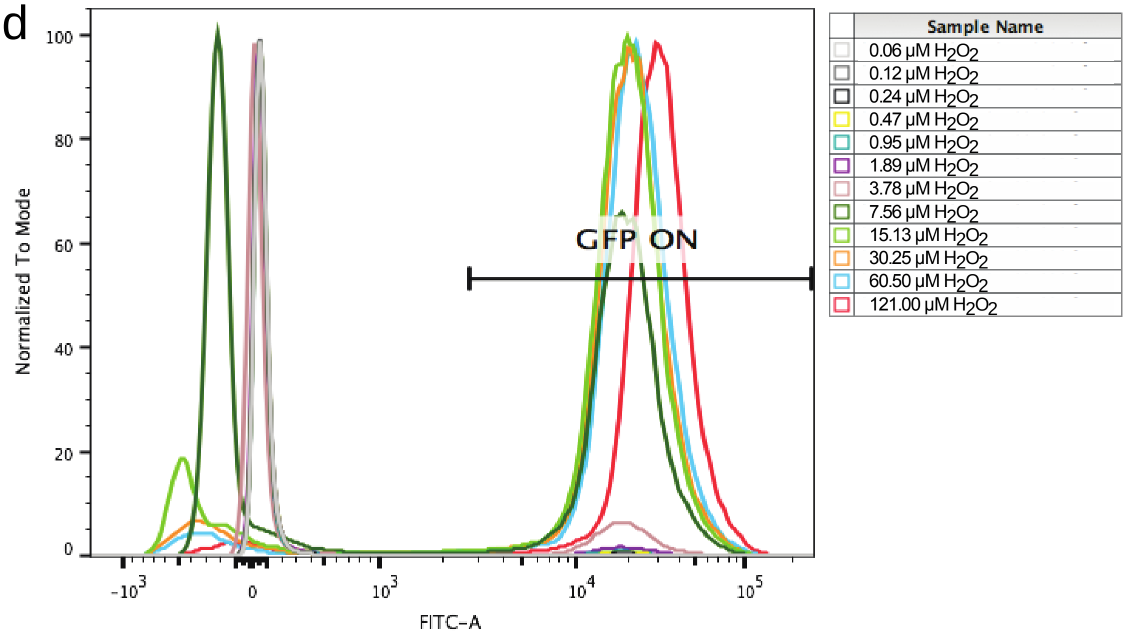

Normalized To Mode
0
20
40
60
80
100
GFP ON
FITC-A
Sample Name
0.06 μM H2O2
0.12 μM H2O2
0.24 μM H2O2
0.47 μM H2O2
0.95 μM H2O2
1.89 μM H2O2
3.78 μM H2O2
7.56 μM H2O2
15.13 μM H2O2
30.25 μM H2O2
60.50 μM H2O2
121.00 μM H2O2

e

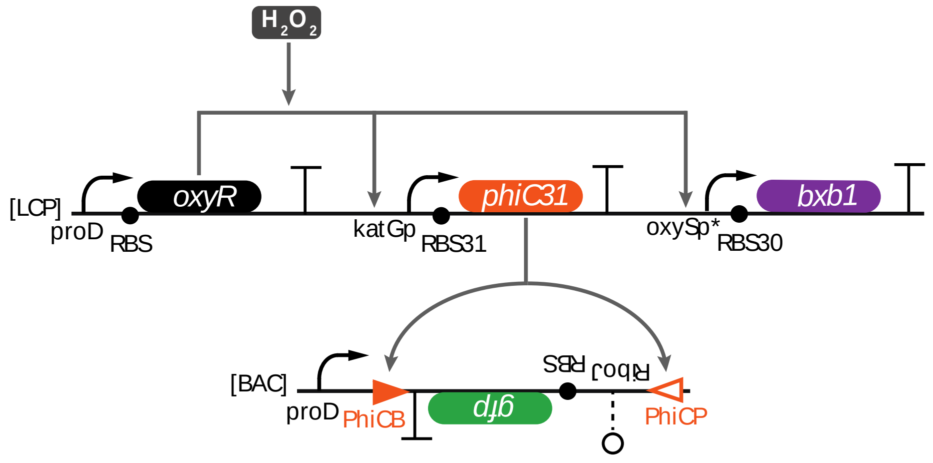

H2O2
[LCP]
proD
RBS
oxyR
katGp
RBS31
phiC31
oxySp*
RBS30
bxb1
[BAC]
proD
PhiCB
gfp
RBS
proJ
PhiCP

f

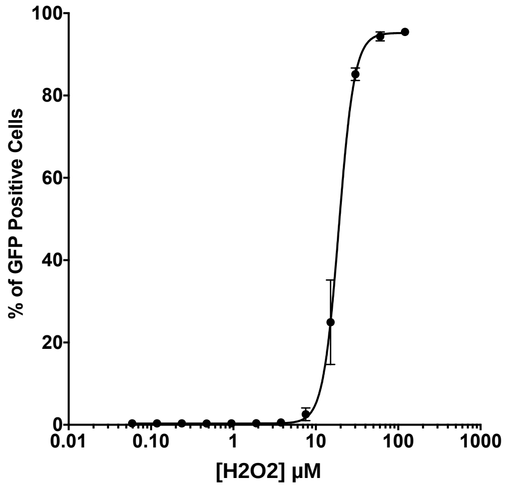

% of GFP Positive Cells
0
20
40
60
80
100
0.01
0.10
1
10
100
1000
[H2O2] μM

g

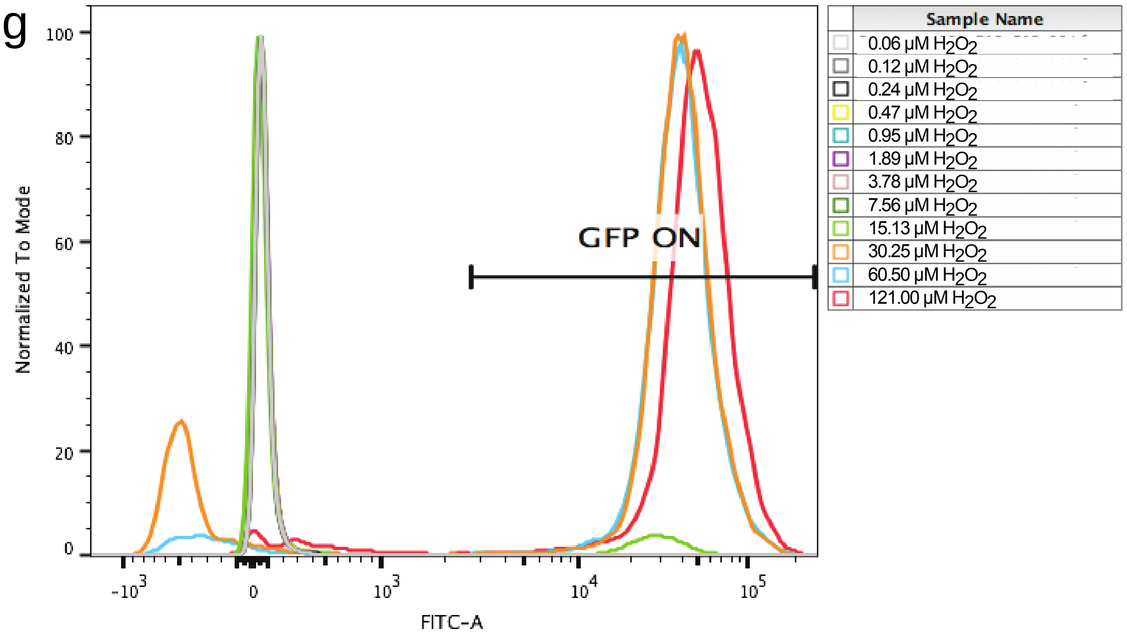

Normalized To Mode
0
20
40
60
80
100
-10^3
0
10^3
10^4
10^5
FITC-A
GFP ON
Sample Name
0.06 μM H2O2
0.12 μM H2O2
0.24 μM H2O2
0.47 μM H2O2
0.95 μM H2O2
1.89 μM H2O2
3.78 μM H2O2
7.56 μM H2O2
15.13 μM H2O2
30.25 μM H2O2
60.50 μM H2O2
121.00 μM H2O2

Supplementary Figure 7 | A bandpass filter assembled from a low-threshold high-pass circuit and a medium-threshold low-pass circuit (Fig. 2a,b).

a. Representative flow cytometry histograms for GFP expression from the bandpass circuit shown in **Fig. 2a,b**.

b. The circuit used to characterize the transfer function of the low-threshold comparator that operates as a high-pass in the bandpass circuit in **Fig. 2a,b**. OxyR is constitutively expressed from a LCP and activates transcription of *bxb1* from the oxySp* promoter and *phiC31* from the katGp promoter on the same LCP in response to H_2O_2. Bxb1 inverts the *gfp* expression cassette on a BAC, turning on GFP expression by pairing it with the proD promoter.

c. The transfer function of the low-threshold comparator that operates as a high-pass in the bandpass circuit in **Fig. 2a,b**. Black line is a sigmoidal fit to the data. This fit was used to generate the high-pass variables in the bandpass function (**Supplementary Information Note 1**). The errors (standard deviation) are derived from flow cytometry experiments of three biological replicates, each of which involved $n >$ 30,000 gated events.

d. Representative flow cytometry histograms for GFP expression for the data shown in **Supplementary Fig. 7c**.

e. The circuit used to characterize the transfer function of the medium-threshold comparator that operates as a low-pass transfer function in the bandpass circuit in **Fig. 2a,b**. Here, we characterized the comparator by turning on GFP expression, rather than turning it off as in **Fig. 2**. OxyR is constitutively expressed from a LCP and activates transcription of *bxb1* from the oxySp* promoter and *phiC31* from the katGp promoter on the same LCP in response to H_2O_2. PhiC31 inverts the *gfp* cassette on a BAC, turning on GFP expression by pairing it with the proD promoter.

f. The transfer function of the medium-threshold comparator that operates as a low-pass in the bandpass circuit in **Fig. 2a,b**. This fit was used to generate the low-pass variables in the bandpass function (**Supplementary Information Note 1**). The errors (standard deviation) are derived from flow cytometry experiments of three biological replicates, each of which involved $n >$ 30,000 gated events.

g. Representative flow cytometry histograms for GFP expression for the data shown in **Supplementary Fig. 7f**.

a

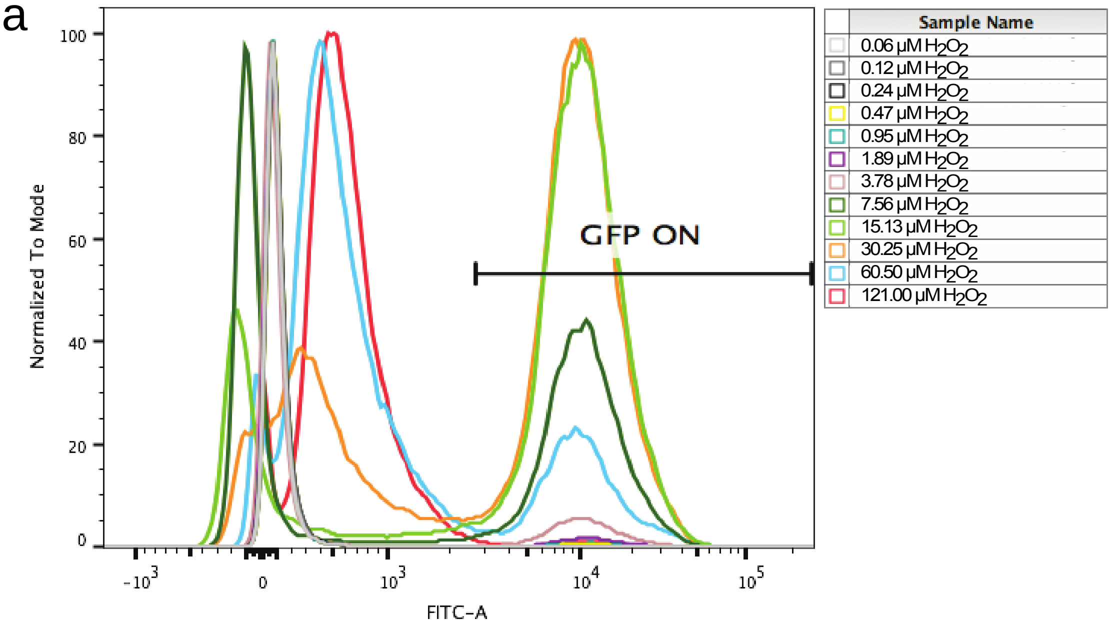

Normalized To Mode
100
80
60
40
20
0
GFP ON
-10^3
0
10^3
10^4
10^5
FITC-A
Sample Name
0.06 μM H2O2
0.12 μM H2O2
0.24 μM H2O2
0.47 μM H2O2
0.95 μM H2O2
1.89 μM H2O2
3.78 μM H2O2
7.56 μM H2O2
15.13 μM H2O2
30.25 μM H2O2
60.50 μM H2O2
121.00 μM H2O2

b

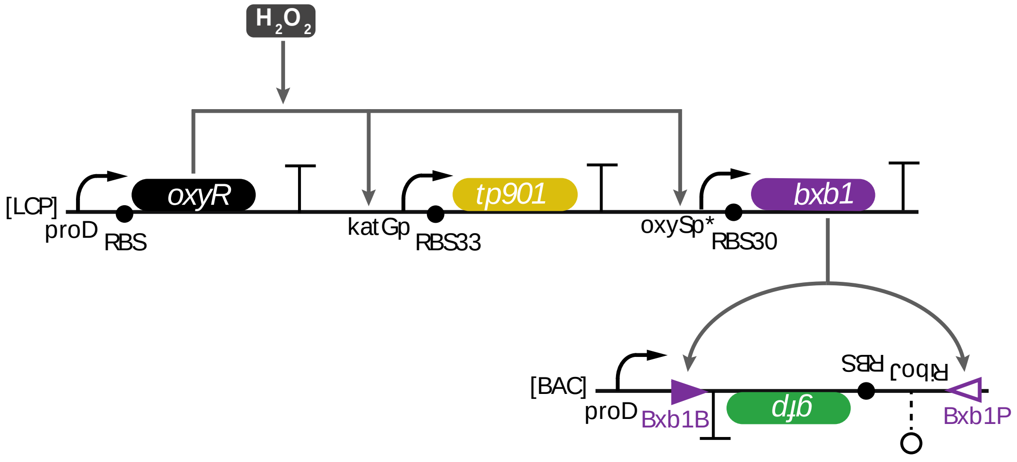
H2O2
[LCP]
proD
RBS
oxyR
katGp
RBS33
tp901
oxySp*
RBS30
bxb1
[BAC]
proD
Bxb1B
Bxb1P

c

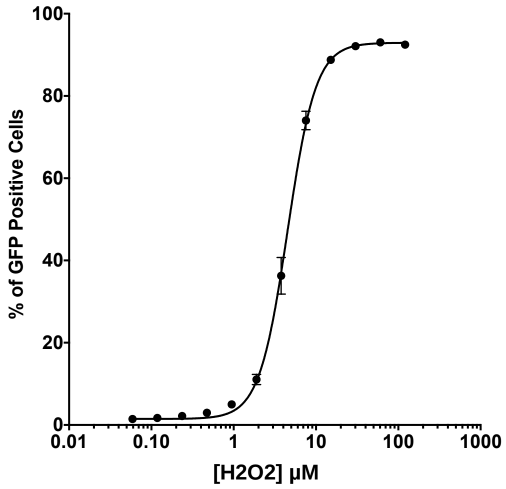
100
80
60
40
20
0
% of GFP Positive Cells
0.01
0.10
1
10
100
1000
[H2O2] μM

d

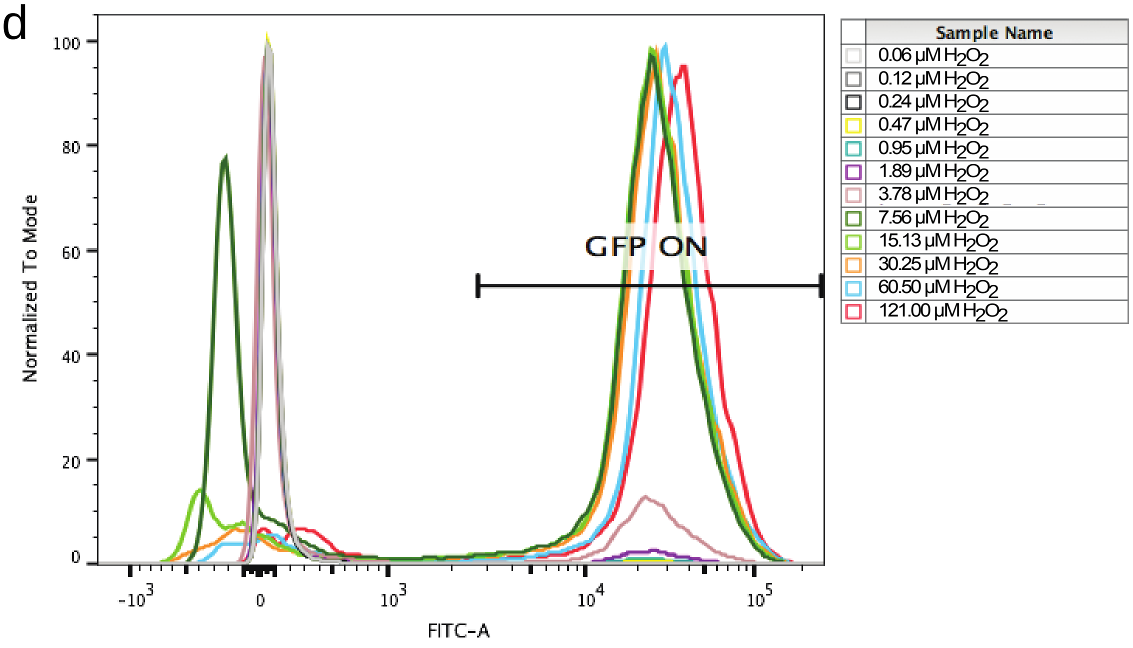
Normalized To Mode
100
80
60
40
20
0
-10^3
0
10^3
10^4
10^5
FITC-A
GFP ON
Sample Name
0.06 μM H2O2
0.12 μM H2O2
0.24 μM H2O2
0.47 μM H2O2
0.95 μM H2O2
1.89 μM H2O2
3.78 μM H2O2
7.56 μM H2O2
15.13 μM H2O2
30.25 μM H2O2
60.50 μM H2O2
121.00 μM H2O2

e

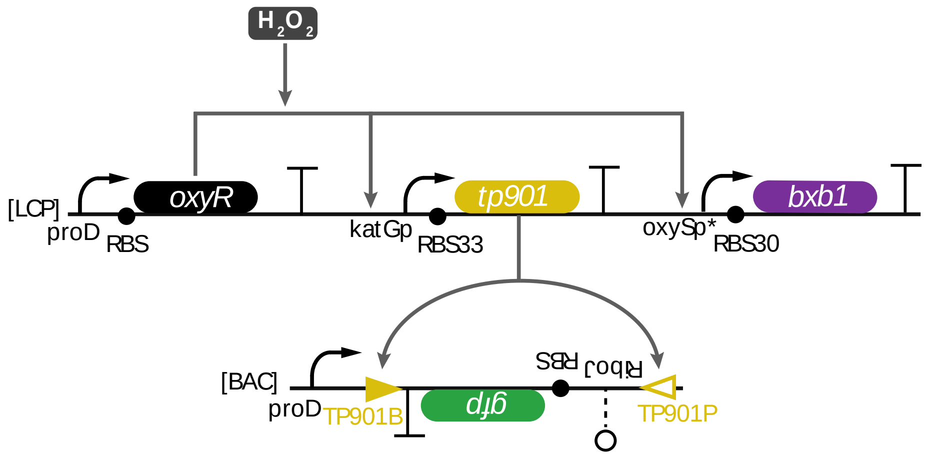

H2O2
[LCP]
proD
RBS
oxyR
katGp
RBS33
tp901
oxySp*
RBS30
bxb1
[BAC]
proD
TP901B
gfp
RBS
RiboJ
TP901P

f

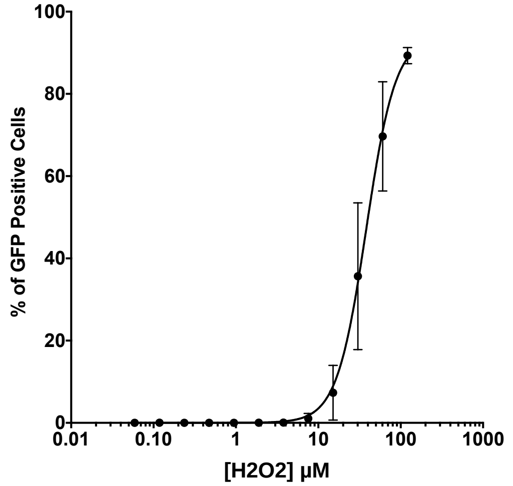

% of GFP Positive Cells
100
80
60
40
20
0
0.01
0.10
1
10
100
1000
[H2O2] μM

g

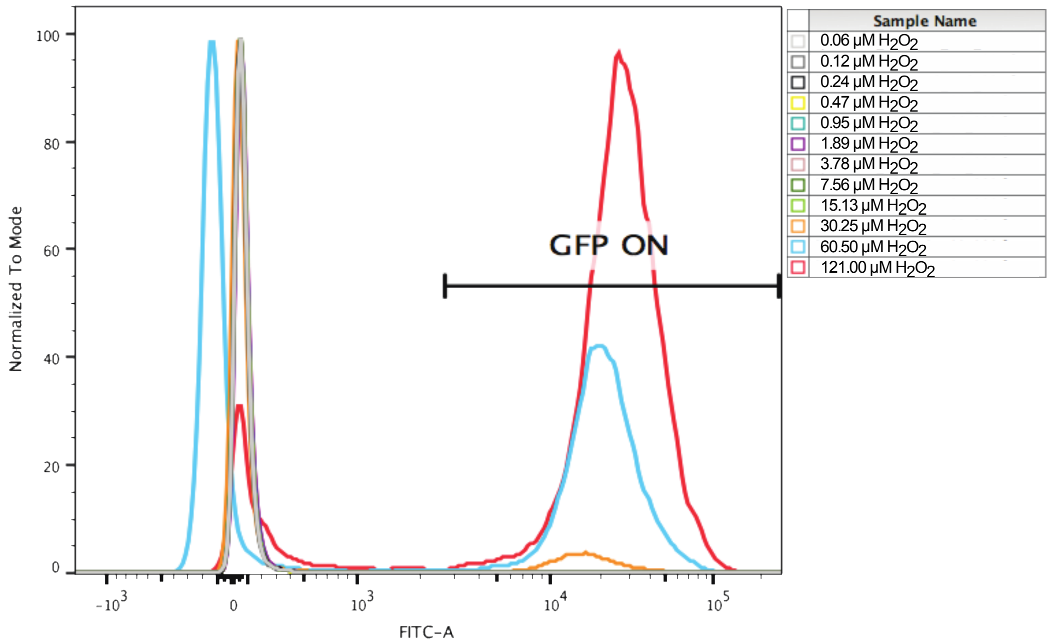

Normalized To Mode
100
80
60
40
20
0
-10^3
0
10^3
10^4
10^5
FITC-A
GFP ON
Sample Name
0.06 μM H2O2
0.12 μM H2O2
0.24 μM H2O2
0.47 μM H2O2
0.95 μM H2O2
1.89 μM H2O2
3.78 μM H2O2
7.56 μM H2O2
15.13 μM H2O2
30.25 μM H2O2
60.50 μM H2O2
121.00 μM H2O2

Supplementary Figure 8 | A bandpass filter assembled from a low-threshold high-pass circuit and a high-threshold low-pass circuit (Fig. 2c,d).

a. Representative flow cytometry histograms for GFP expression from the bandpass circuit shown in **Fig. 2c,d**.

b. The circuit used to characterize the transfer function of the low-threshold comparator that operates as a high-pass in the bandpass circuit in **Fig. 2c,d**. OxyR is constitutively expressed from a LCP and activates transcription of *bxb1* from the oxySp* promoter and *tp901* from the katGp promoter on the same LCP in response to H_2O_2. Bxb1 inverts the *gfp* cassette on a BAC, turning on GFP expression by pairing it with the proD promoter.

c. The transfer function of the low-threshold comparator that operates as a high-pass in the bandpass circuit in **Fig. 2c,d**. This fit was used to generate the high-pass variables in the bandpass function (**Supplementary Information Note 1**). The errors (standard deviation) are derived from flow cytometry experiments of three biological replicates, each of which involved $n > 30,000$ gated events.

d. Representative flow cytometry histograms for GFP expression for the data shown in **Supplementary Fig. 8c**.

e. The circuit used to characterize the transfer function of the high-threshold comparator that operates as a low-pass transfer function in the bandpass circuit in **Fig. 2c,d**. Here, we characterized the comparator by turning on GFP expression, rather than turning it off as in **Fig. 2**. OxyR is constitutively expressed from a LCP and activates transcription of *bxb1* from the oxySp* promoter and *tp901* from the katGp promoter on the same LCP in response to H_2O_2. TP901 inverts the *gfp* cassette on a BAC, turning on GFP expression by pairing it with the proD promoter.

f. The transfer function of the high-threshold comparator that operates as a low-pass in the bandpass circuit in **Fig. 2c,d**. This fit was used to generate the low-pass variables in the bandpass function (**Supplementary Information Note 1**).

g. Representative flow cytometry histograms for GFP expression for the data shown in **Supplementary Fig. 8f**.

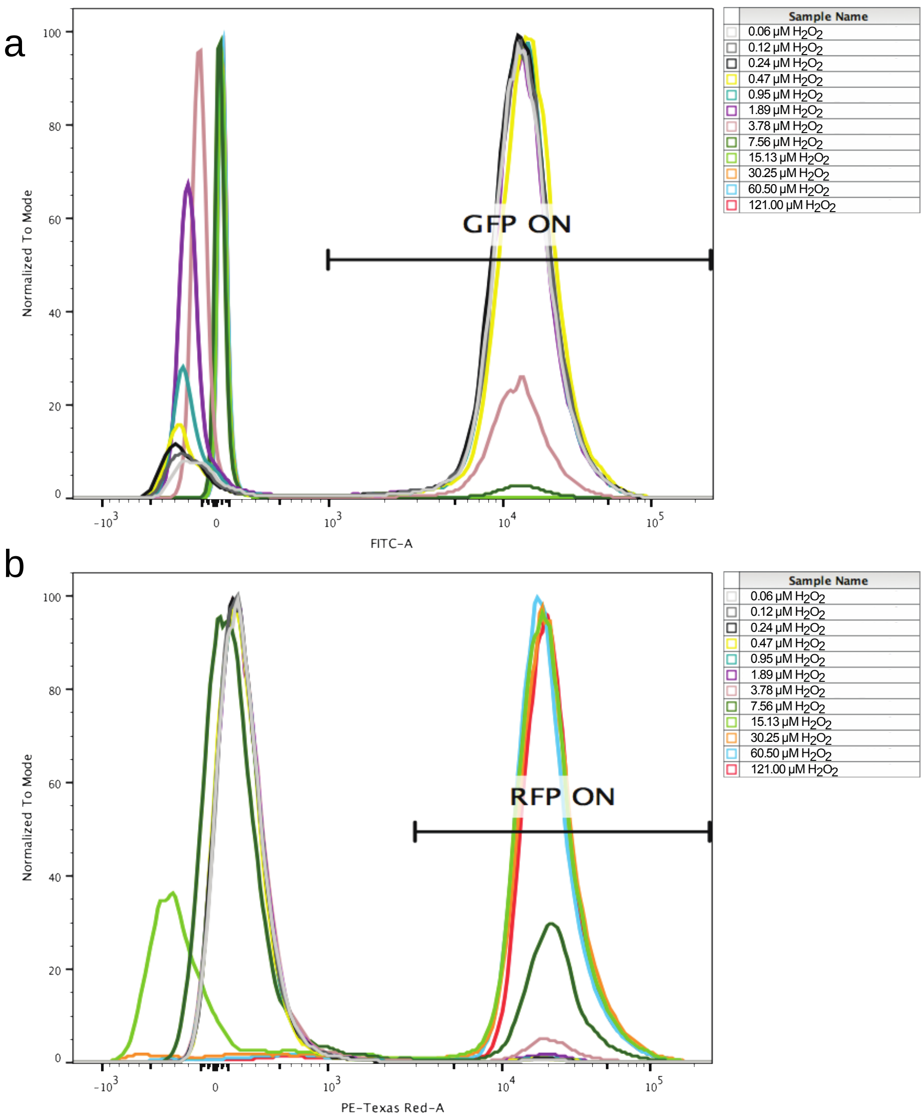
a
b
Sample Name
0.06 µM H2O2
0.12 µM H2O2
0.24 µM H2O2
0.47 µM H2O2
0.95 µM H2O2
1.89 µM H2O2
3.78 µM H2O2
7.56 µM H2O2
15.13 µM H2O2
30.25 µM H2O2
60.50 µM H2O2
121.00 µM H2O2
GFP ON
RFP ON
Normalized To Mode
FITC-A
PE-Texas Red-A

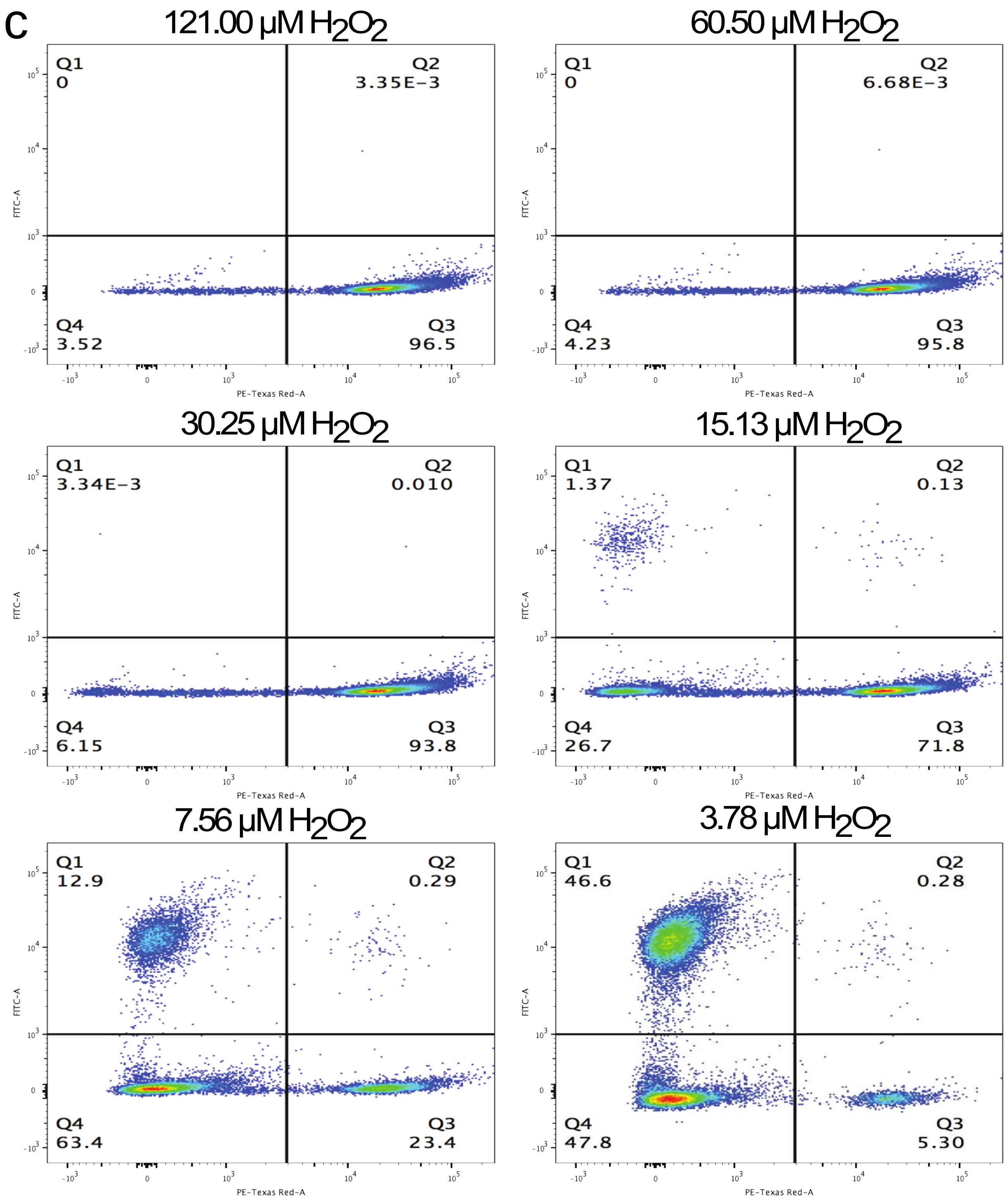
C
121.00 µM H2O2
60.50 µM H2O2
30.25 µM H2O2
15.13 µM H2O2
7.56 µM H2O2
3.78 µM H2O2
Q1 0
Q2 3.35E-3
Q4 3.52
Q3 96.5
Q1 0
Q2 6.68E-3
Q4 4.23
Q3 95.8
Q1 3.34E-3
Q2 0.010
Q4 6.15
Q3 93.8
Q1 1.37
Q2 0.13
Q4 26.7
Q3 71.8
Q1 12.9
Q2 0.29
Q4 63.4
Q3 23.4
Q1 46.6
Q2 0.28
Q4 47.8
Q3 5.30
FITC-A
PE-Texas Red-A

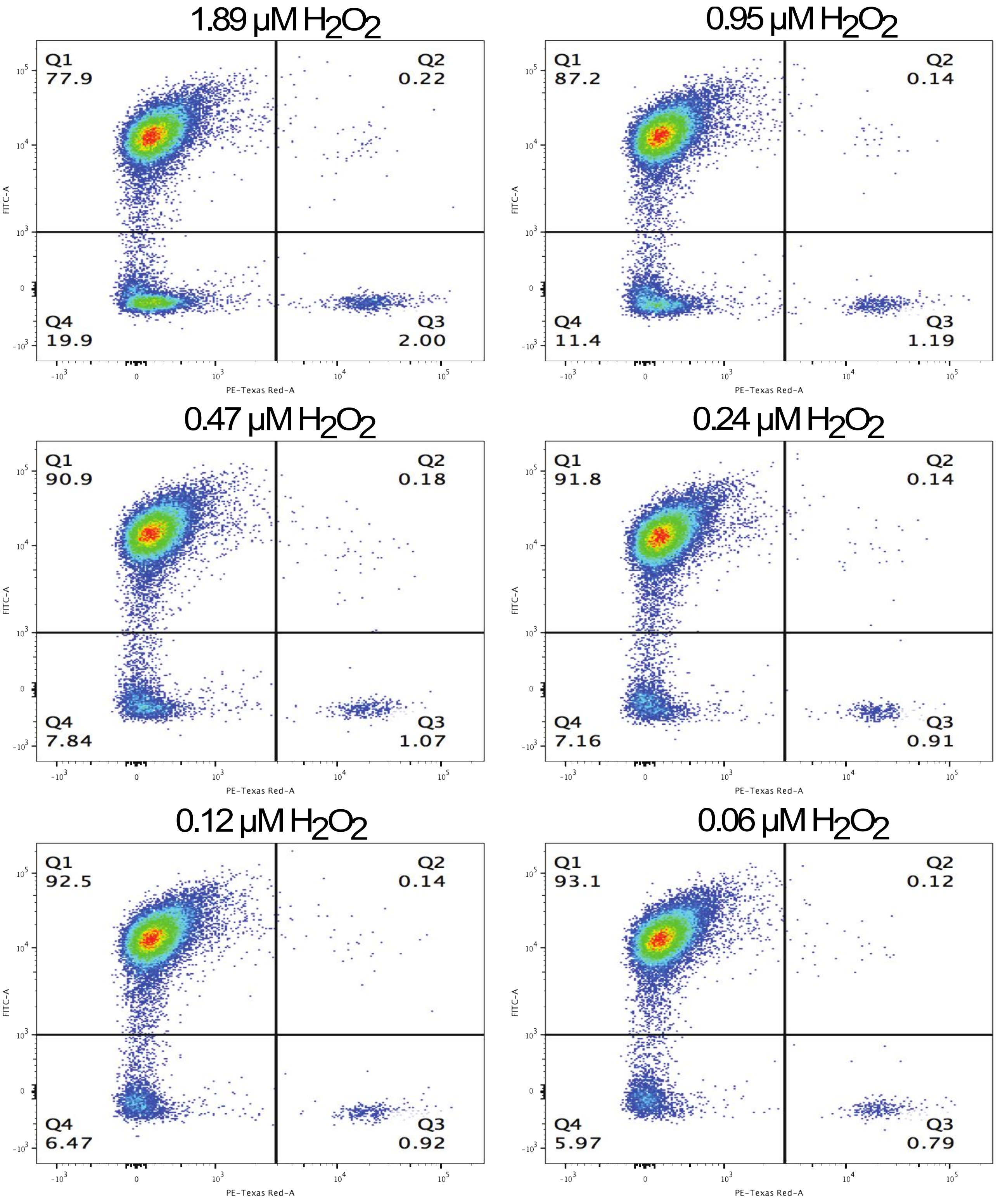

Supplementary Figure 9 | Ternary logic (Fig. 3a,b).

a. Representative flow cytometry histograms for GFP expression for the ternary logic circuit shown in **Fig. 3a**, and the data in **Fig. 3b**.

b. Representative flow cytometry histograms for RFP expression for the ternary logic circuit shown in **Fig. 3a**, and the data in **Fig. 3b**.

c. Representative flow cytometry plots for GFP and RFP expression for the ternary logic circuit shown in **Fig. 3a**, and the data in **Fig. 3b**.

a
b
c

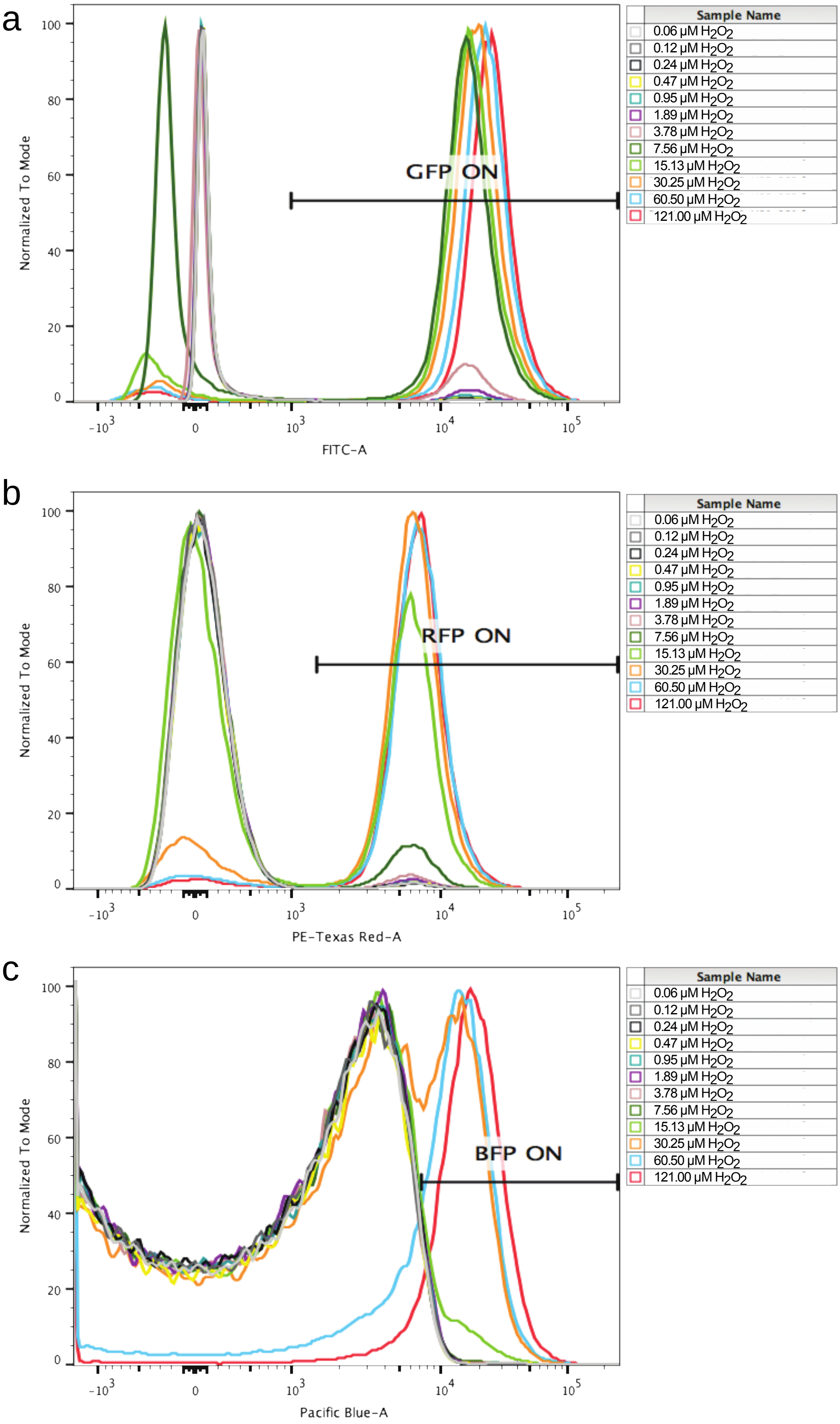

Normalized To Mode
100
80
60
40
20
0
-10^3
0
10^3
10^4
10^5
FITC-A
GFP ON
PE-Texas Red-A
RFP ON
Pacific Blue-A
BFP ON
Sample Name
0.06 μM H_2O_2
0.12 μM H_2O_2
0.24 μM H_2O_2
0.47 μM H_2O_2
0.95 μM H_2O_2
1.89 μM H_2O_2
3.78 μM H_2O_2
7.56 μM H_2O_2
15.13 μM H_2O_2
30.25 μM H_2O_2
60.50 μM H_2O_2
121.00 μM H_2O_2

Supplementary Figure 10 | 2-bit Analog-to-digital Converter. (Fig. 3d,e).

a. Representative flow cytometry histograms for GFP expression from the analog-to-digital converter circuit shown in **Fig. 3d**, and the data in **Fig. 3e**.

b. Representative flow cytometry histograms for RFP expression from the analog-to-digital converter circuit shown in **Fig. 3d**, and the data in **Fig. 3e**.

c. Representative flow cytometry histograms for BFP expression from the analog-to-digital converter circuit shown in **Fig. 3d**, and the data in **Fig. 3e**.

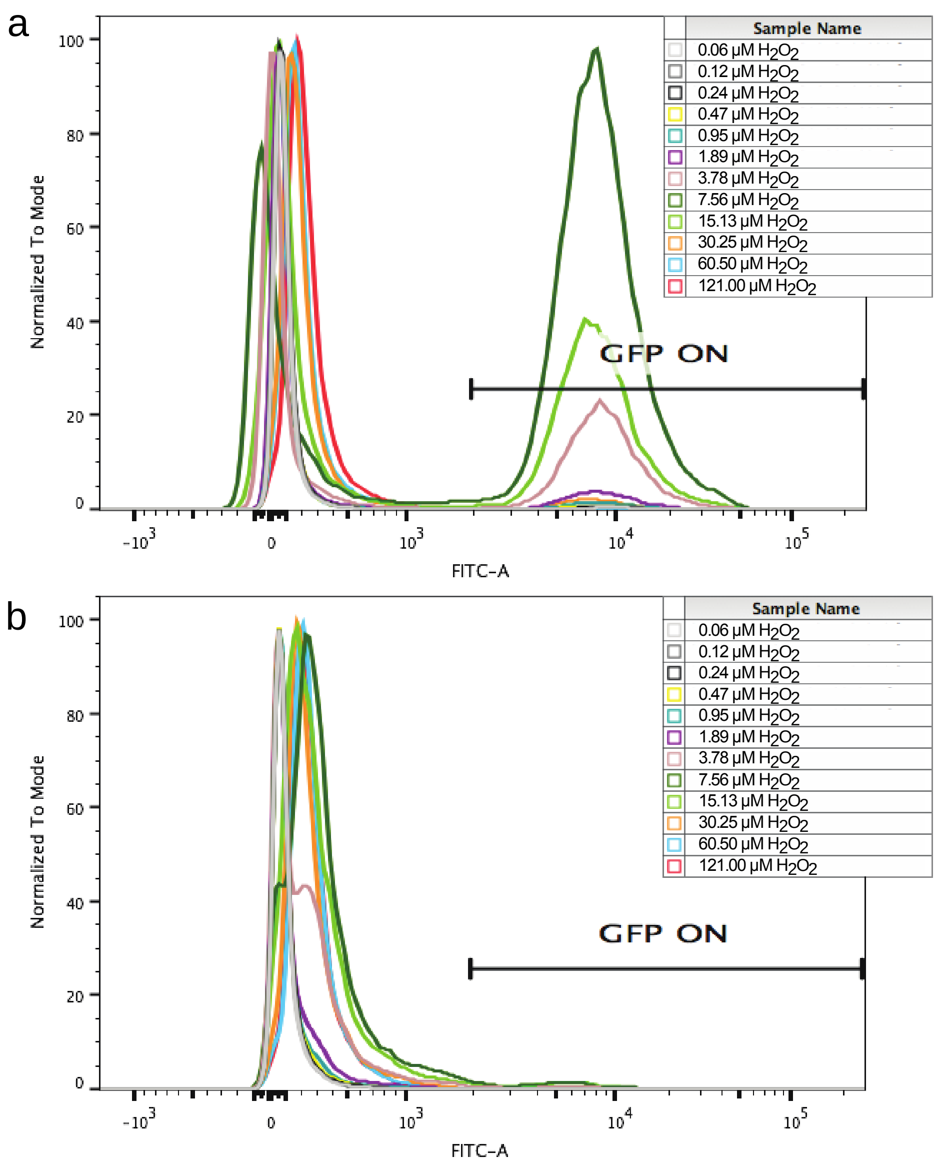

Supplementary Figure 11 | Mixed-signal processing and concentration-dependent logic (Fig. 4).

a. Representative flow cytometry histograms for GFP expression from the mixed-signal processing circuit shown in **Fig. 4a**, and the data in **Fig. 4b**, without aTc.

b. Representative flow cytometry histograms for GFP expression from the mixed- signal processing circuit shown in **Fig. 4a**, and the data in **Fig. 4b**, with aTc.

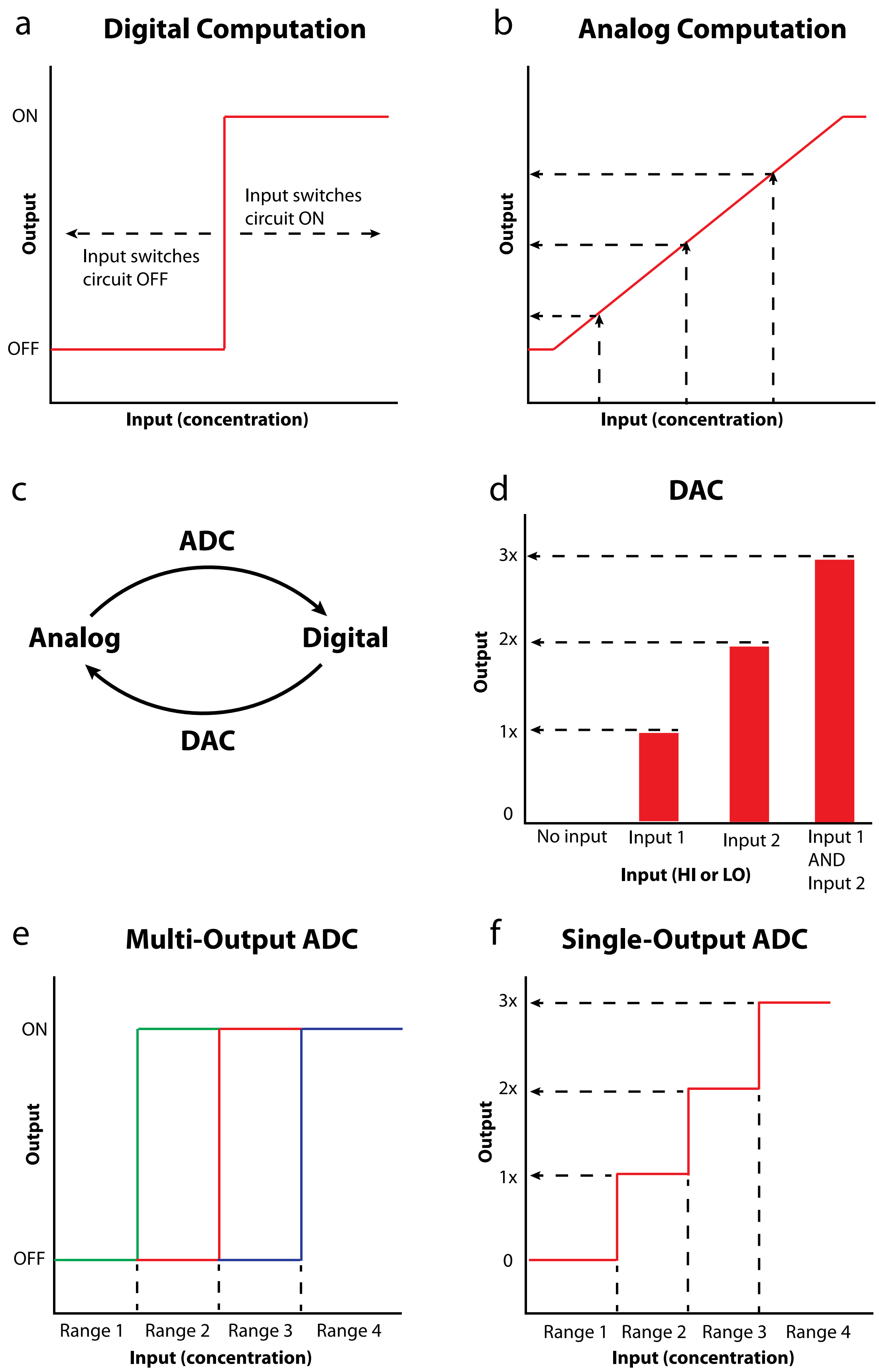

a
Digital Computation
ON
OFF
Output
Input switches circuit ON
Input switches circuit OFF
Input (concentration)
b
Analog Computation
Output
Input (concentration)
c
ADC
Analog
Digital
DAC
d
DAC
3x
2x
1x
0
Output
No input
Input 1
Input 2
Input 1 AND Input 2
Input (HI or LO)
e
Multi-Output ADC
ON
OFF
Output
Range 1
Range 2
Range 3
Range 4
Input (concentration)
f
Single-Output ADC
3x
2x
1x
0
Output
Range 1
Range 2
Range 3
Range 4
Input (concentration)

Supplementary Figure 12 | Digital-to-analog converters and analog-to-digital converters are complementary systems that translate digital signals to analog signals, and vice versa.

a. In the digital computation paradigm, signals are defined as OFF or ON and computing is based on Boolean logic.

b. In the analog computation paradigm, circuits convert continuous, analog inputs to continuous outputs according to mathematical relationships.

c. Analog information is converted to digital information with analog-to-digital converters (ADC). Digital information is converted to analog information with digital-to-analog converters (DAC).

d. A digital-to-analog converter that accepts various digital combinations of inputs and outputs quantized levels of a single output.

e. An analog-to-digital converter that accepts the continuous, analog concentration of an input and classifies discrete ranges of the input to different output molecules.

f. An analog-to-digital converter that accepts the continuous, analog concentration of an input and classifies discrete ranges of the input to discrete levels of a single output.

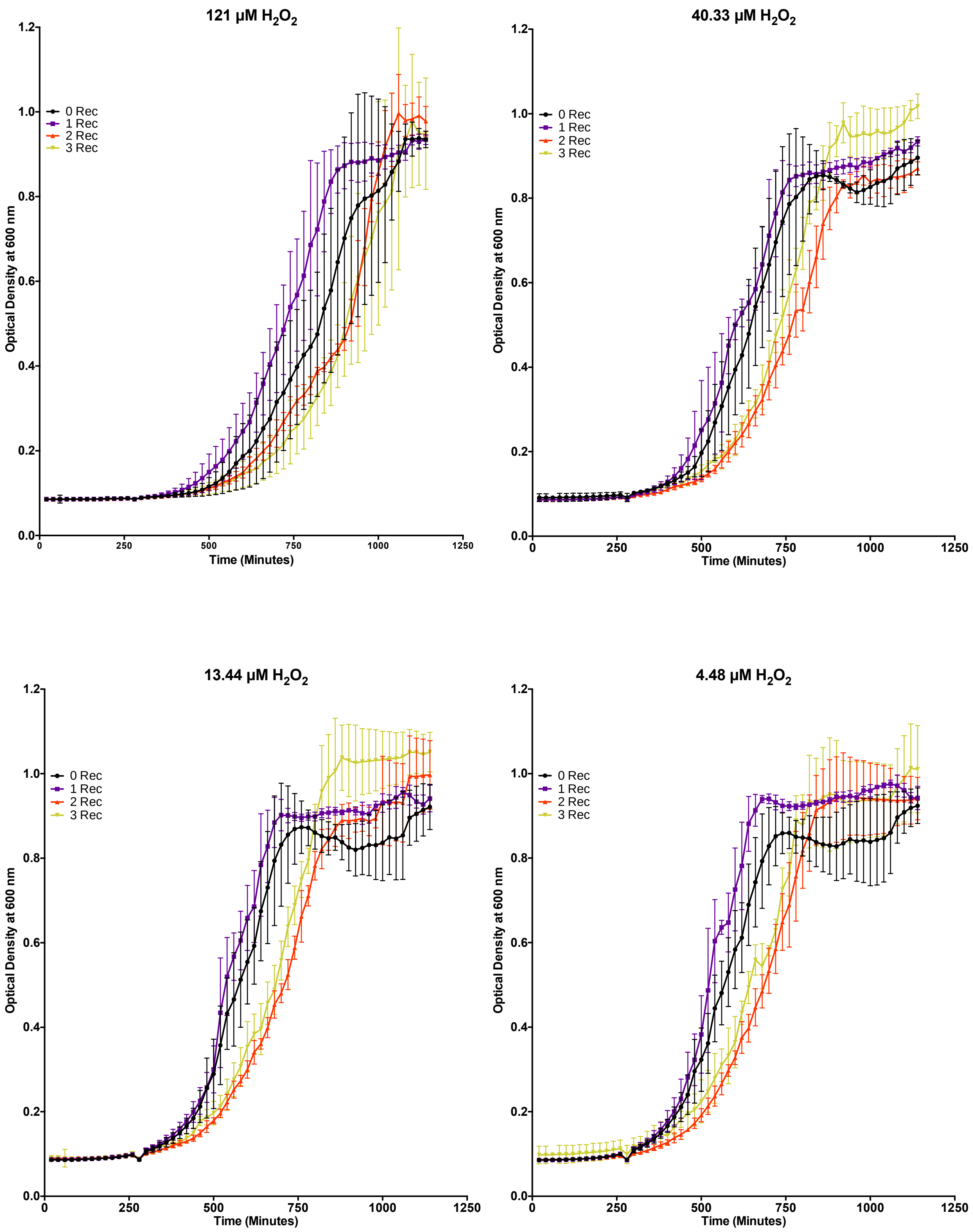
121 μM H2O2
40.33 μM H2O2
13.44 μM H2O2
4.48 μM H2O2
Optical Density at 600 nm
Time (Minutes)
0 Rec
1 Rec
2 Rec
3 Rec
0.0
0.2
0.4
0.6
0.8
1.0
1.2
0
250
500
750
1000
1250

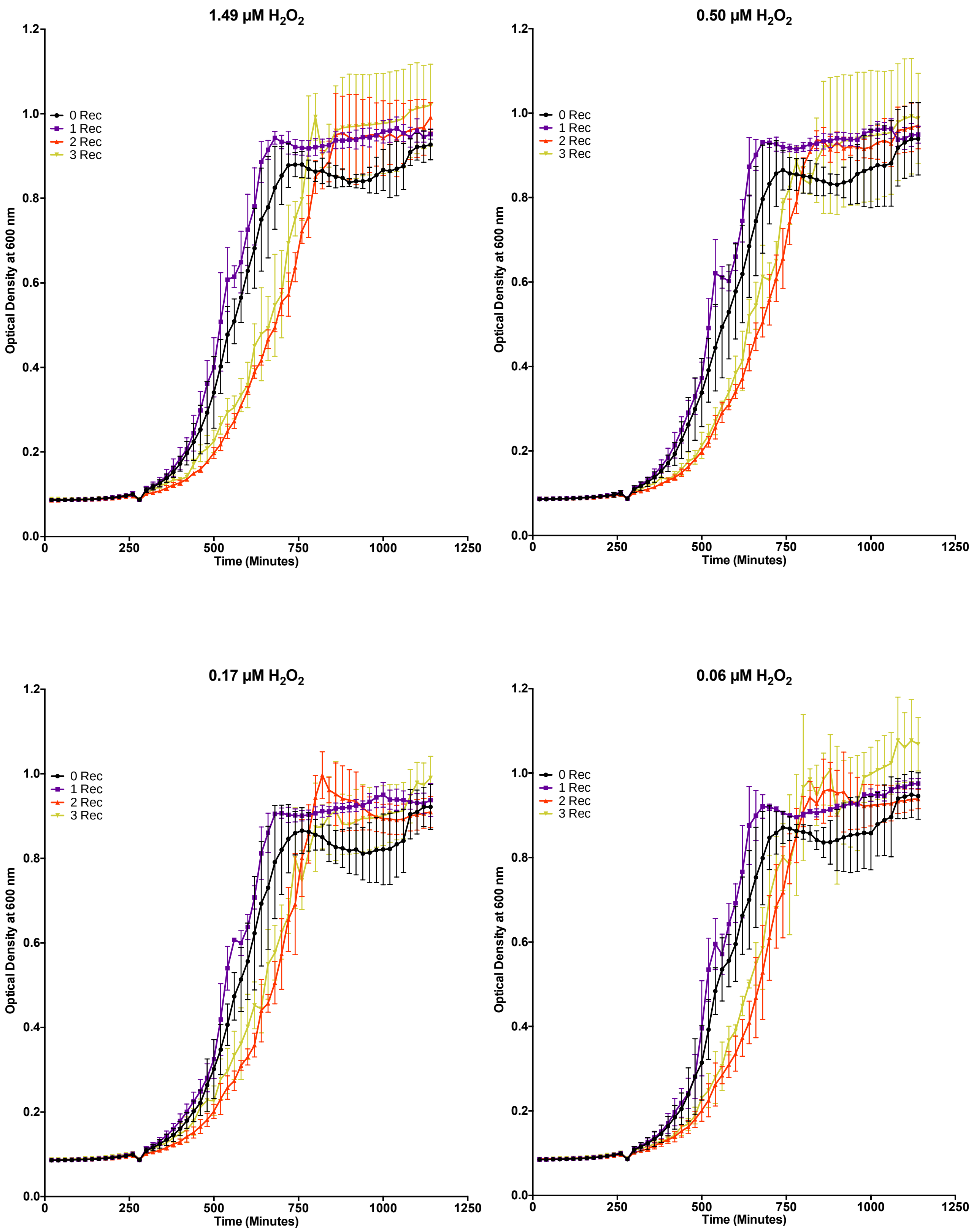

1.49 µM H2O2
0.50 µM H2O2
0.17 µM H2O2
0.06 µM H2O2
0 Rec
1 Rec
2 Rec
3 Rec
Optical Density at 600 nm
Time (Minutes)

Supplementary Figure 13 | Growth curves for cells containing 0, 1, 2, or 3 recombinases at different concentrations of H_2O_2. Cells were induced with H_2O_2 and incubated in a plate reader shaking at 30 degrees for 20 hours. Optical density measurements at 600 nm were taken every 20 minutes. The mean and standard deviation are derived from three biological replicates, and the lines are direct connections between adjacent measurements. The cells did not contain reporter plasmids. The cells with 1 recombinase (Rec) contain the low-pass circuit encoded on 1 plasmid (pZS2oxySp*-RBS30-Bxbi-proD-oxyR). The cells with 2 recombinases contain the low-pass and medium-pass circuit on 2 plasmids (pZS2oxySp*-RBS30-Bxbi-proD-oxyR and pZS1katGp-RBS31-PhiC31-proD-oxyR). The cells with 3 recombinases contain the low-pass, medium-pass, and high-pass circuit on 2 plasmids (pZS1oxySp*-RBS30-bxbi-katGp-RBS31-PhiC31-proD-oxyR + pZS2katGp-RBS33-TP901-proD-oxyR).

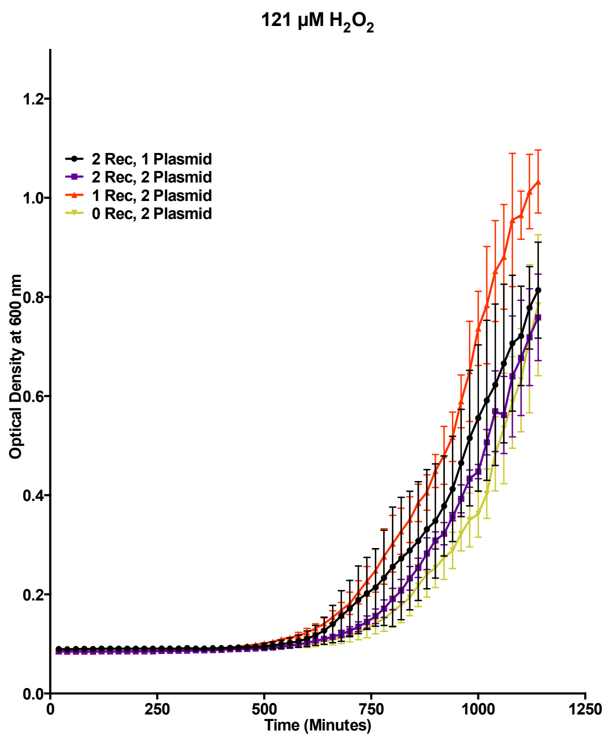
121 μM H2O2
2 Rec, 1 Plasmid
2 Rec, 2 Plasmid
1 Rec, 2 Plasmid
0 Rec, 2 Plasmid
Optical Density at 600 nm
Time (Minutes)

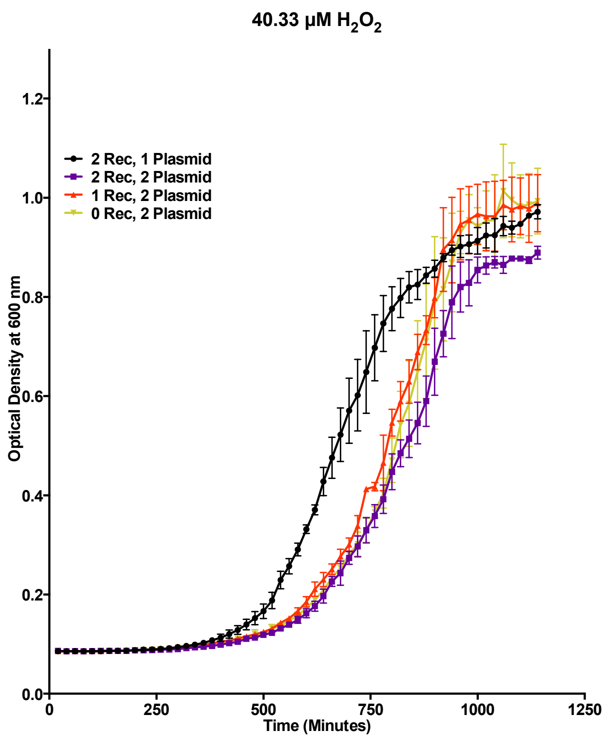
40.33 μM H2O2
2 Rec, 1 Plasmid
2 Rec, 2 Plasmid
1 Rec, 2 Plasmid
0 Rec, 2 Plasmid
Optical Density at 600 nm
Time (Minutes)

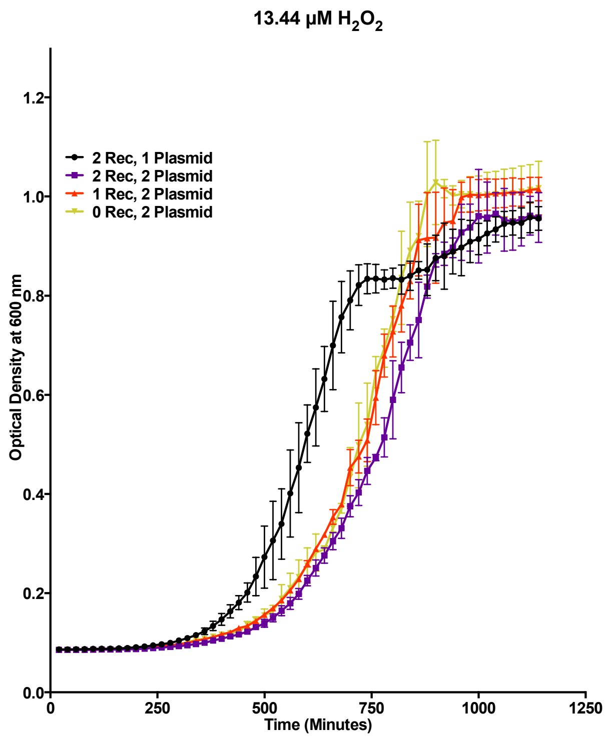
13.44 μM H2O2
2 Rec, 1 Plasmid
2 Rec, 2 Plasmid
1 Rec, 2 Plasmid
0 Rec, 2 Plasmid
Optical Density at 600 nm
Time (Minutes)

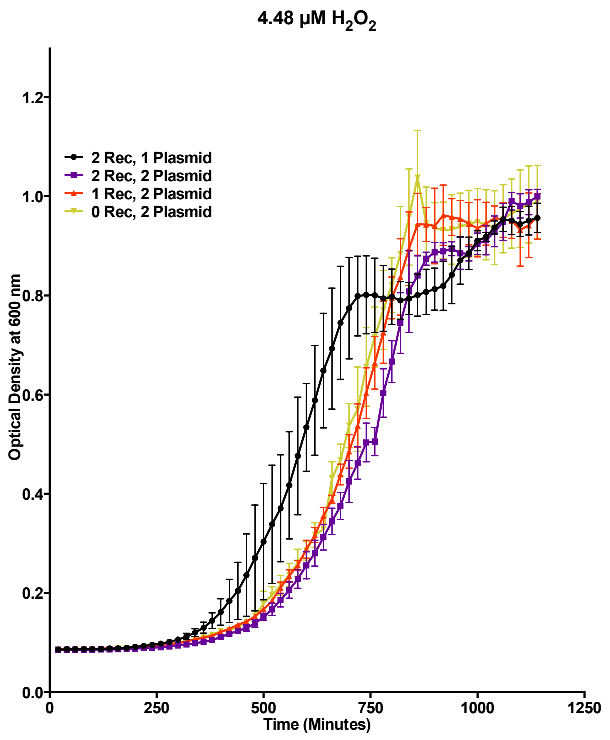
4.48 μM H2O2
2 Rec, 1 Plasmid
2 Rec, 2 Plasmid
1 Rec, 2 Plasmid
0 Rec, 2 Plasmid
Optical Density at 600 nm
Time (Minutes)

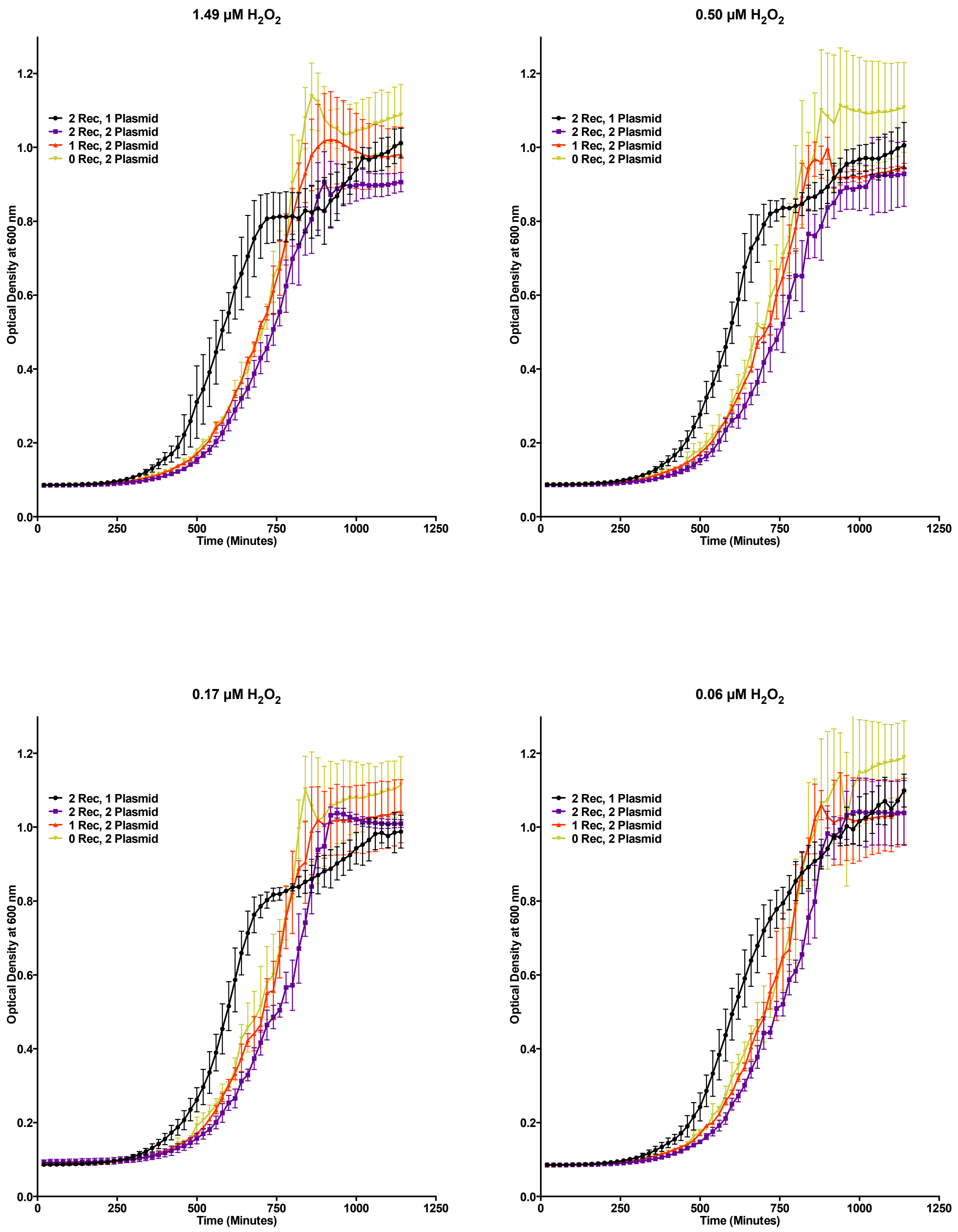
1.49 μM H2O2
0.50 μM H2O2
0.17 μM H2O2
0.06 μM H2O2
2 Rec, 1 Plasmid
2 Rec, 2 Plasmid
1 Rec, 2 Plasmid
0 Rec, 2 Plasmid
Optical Density at 600 nm
Time (Minutes)
0.0
0.2
0.4
0.6
0.8
1.0
1.2
0
250
500
750
1000
1250

Supplementary Figure 14 | Growth curves for cells containing 0, 1, or 2 recombinases on 2 plasmids or 2 recombinases on 1 plasmid at different concentrations of H_2O_2. Cells were induced with H_2O_2 and incubated in a plate reader shaking at 30 degrees for 20 hours. Optical density measurements at 600 nm were taken every 20 minutes. The mean and standard deviation are derived from three biological replicates, and the lines are direct connections between adjacent measurements. The cells did not contain reporter plasmids. The cells with 2 recombinases (Rec) on 1 plasmid contain a plasmid encoding the low-threshold and medium-threshold circuits (plasmid pZS1oxySp*-RBS30-bxbi-katGp-RBS31-PhiC31-proD-oxyR). The cells with 2 recombinases on 2 plasmids contain the low-pass and medium-pass circuit (pZS2oxySp*-RBS30-Bxbi-proD-oxyR and pZS1katGp-RBS31-PhiC31-proD-oxyR). The cells with 1 recombinase and 2 plasmids contain the low-pass circuit (pZS2oxySp*-RBS30-Bxbi-proD-oxyR) and a second plasmid that does not encode a recombinase (pSC101 origin, carbenicillin resistance). The cells with 0 recombinase and 2 plasmids contain 2 plasmids that do not express recombinases (pSC101 origins, carbenicillin or kanamycin resistance).

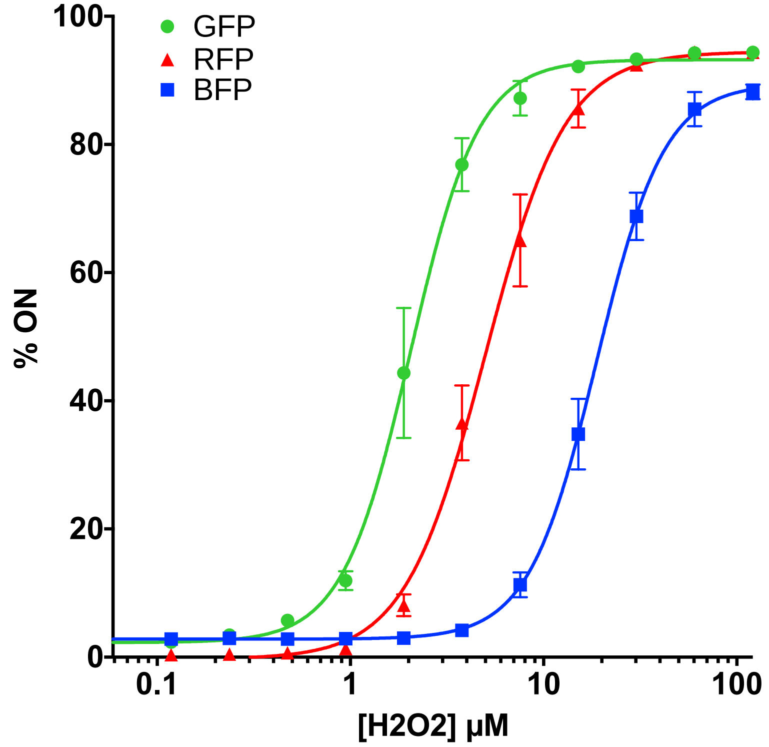

Supplementary Figure 15 | Scale-up of the 2-bit ADC circuit. Cells containing the 2-bit ADC circuit (**Fig. 3d**) were grown within flasks with different concentrations of H_2O_2 at a volume of 20 mL, which is a 100x greater volume than which was used to generate the data in **Fig. 3e**. The thresholds were shifted slightly to lower concentrations of H_2O_2 in higher volumes compared to **Fig. 3e** but still show good separation. The data is the mean and standard deviation of the percent of fluorophore-positive cells from flow cytometry experiments with three biological replicates.

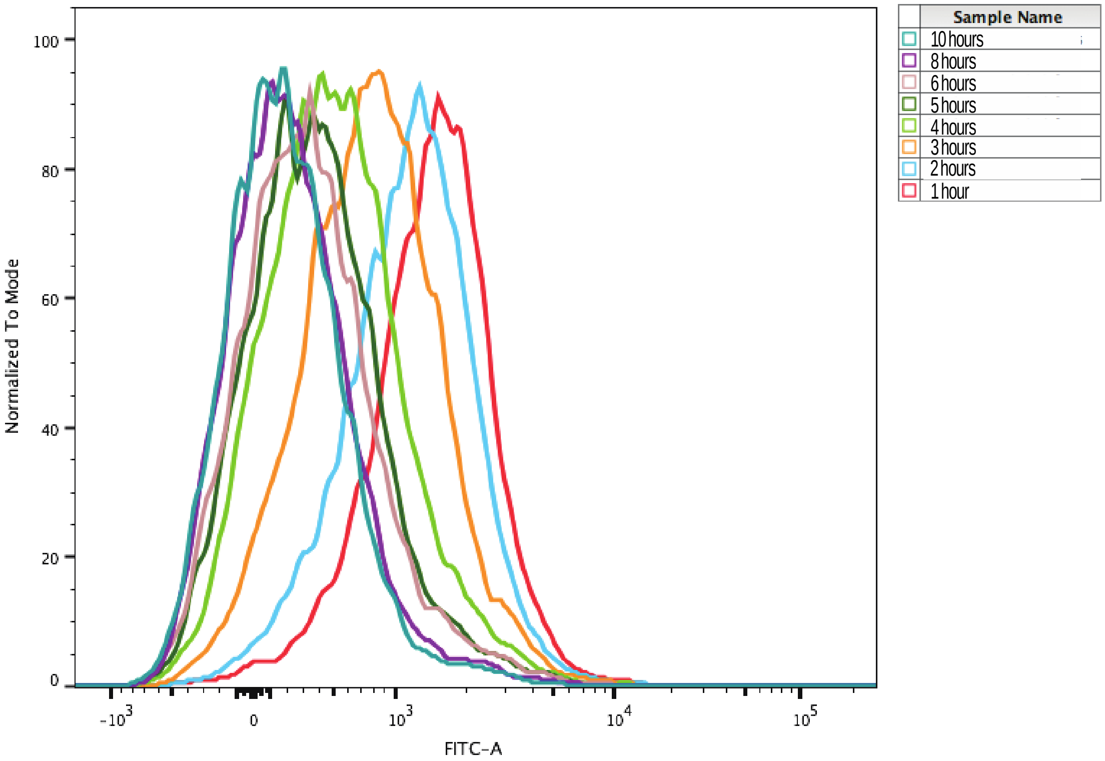
Sample Name
10 hours
8 hours
6 hours
5 hours
4 hours
3 hours
2 hours
1 hour
Normalized To Mode
0
20
40
60
80
100
-10^3
0
10^3
10^4
10^5
FITC-A

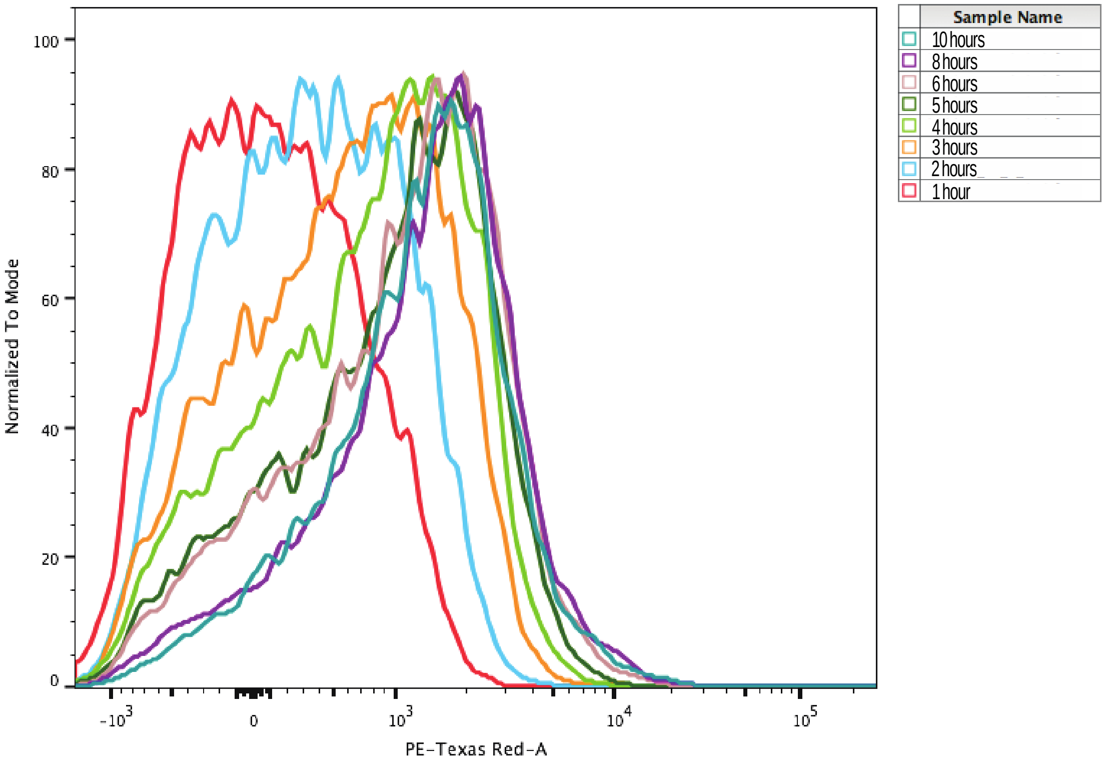
Sample Name
10 hours
8 hours
6 hours
5 hours
4 hours
3 hours
2 hours
1 hour
Normalized To Mode
0
20
40
60
80
100
-10^3
0
10^3
10^4
10^5
PE-Texas Red-A

Supplementary Figure 16 | Time-course experiment for the ternary logic circuit. Representative flow cytometry histograms from three biological replicates for GFP expression (top) and RFP expression (bottom) from the ternary logic circuit shown in **Fig. 3a** at a fixed concentration of H_2O_2 (20.2 μM), which is expected to result in a RFP ON and GFP OFF state. The cells were induced in 50 ml of media in shaking flasks. At each indicated time point, samples were taken from batch culture and run on the flow cytometer. Because the cells were not induced with Copy Control, the separation between ON and OFF states is smaller than measured in Supplementary Figure 9. The cells likely induce recombination within the first 2 hours, as shown by the population-level changes in RFP and GFP expression, though it takes more time for the cells to reach steady-state gene expression by accumulating RFP and diluting GFP through cell division.

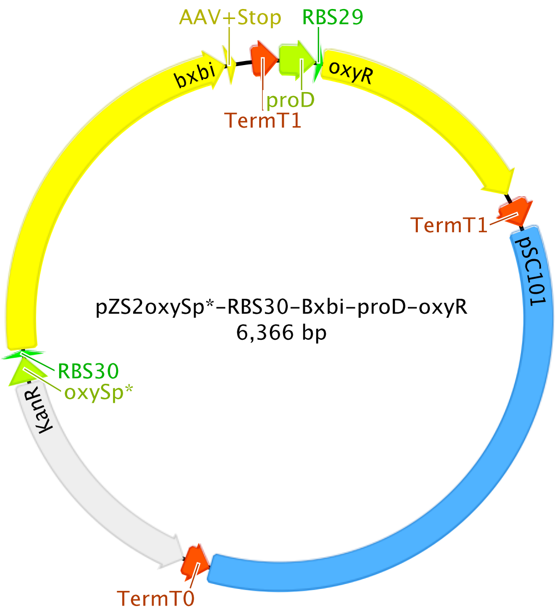

AAV+Stop
RBS29
bxbi
oxyR
proD
TermT1
TermT1
pSC101
pZS2oxySp*-RBS30-Bxbi-proD-oxyR
6,366 bp
RBS30
oxySp*
KanR
TermT0

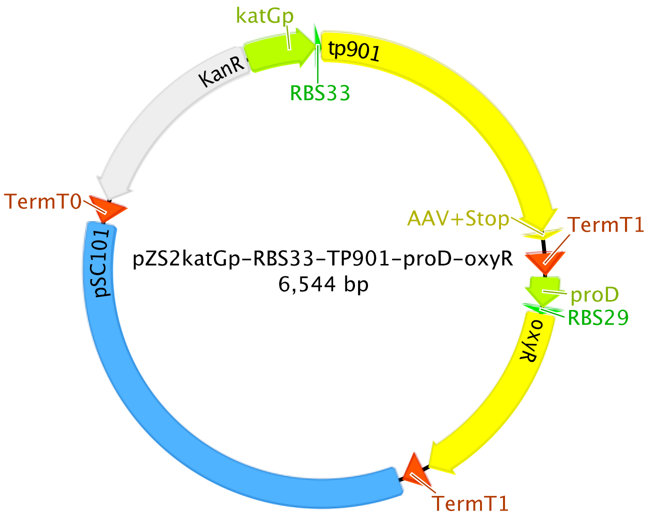

katGp
KanR
tp901
RBS33
TermT0
AAV+Stop
TermT1
pSC101
pZS2katGp-RBS33-TP901-proD-oxyR
6,544 bp
proD
RBS29
oxyR
TermT1

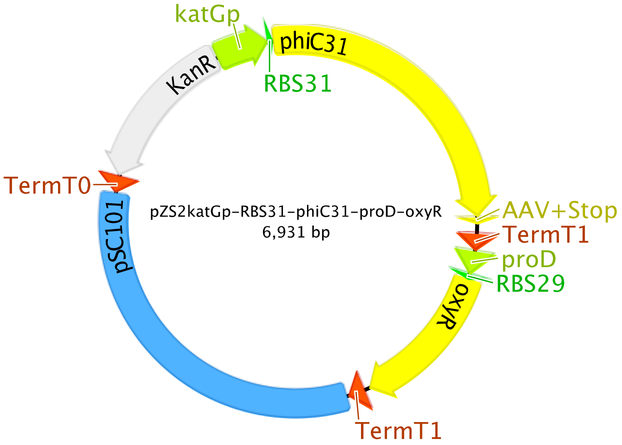

katGp
phiC31
KanR
RBS31
TermT0
AAV+Stop
TermT1
pSC101
pZS2katGp-RBS31-phiC31-proD-oxyR
6,931 bp
proD
RBS29
oxyR
TermT1

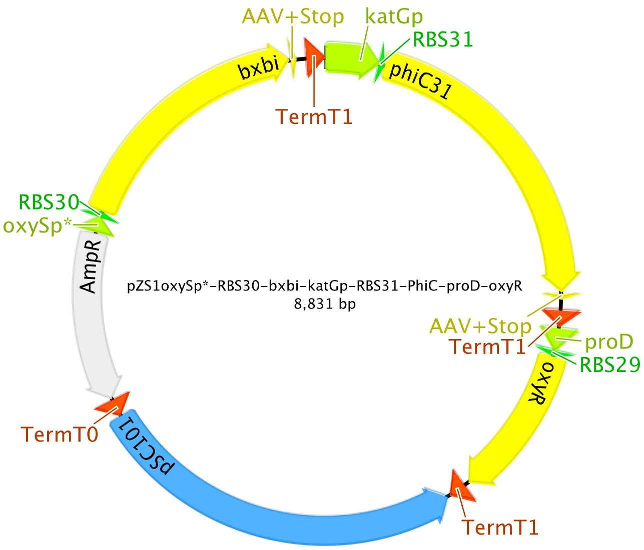

AAV+Stop
katGp
RBS31
bxbi
phiC31
TermT1
RBS30
oxySp*
AmpR
pZS1oxySp*-RBS30-bxbi-katGp-RBS31-PhiC-proD-oxyR
8,831 bp
AAV+Stop
TermT1
proD
RBS29
oxyR
TermT0
pSC101
TermT1

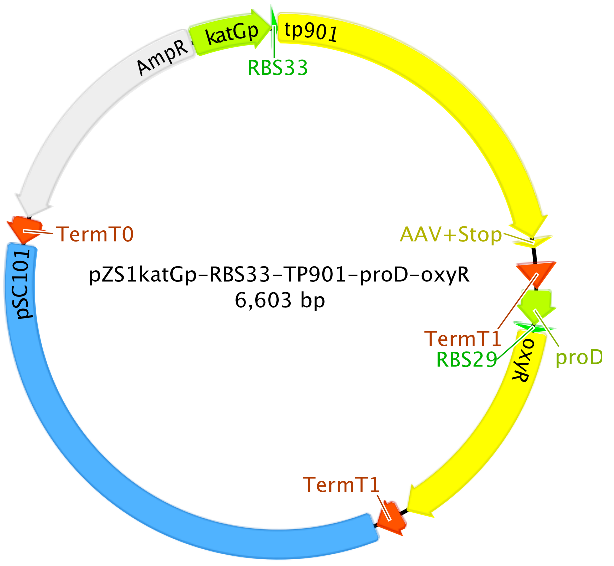
pZS1katGp-RBS33-TP901-proD-oxyR
6,603 bp
AmpR
katGp
tp901
RBS33
TermT0
pSC101
AAV+Stop
TermT1
RBS29
oxyR
proD
TermT1

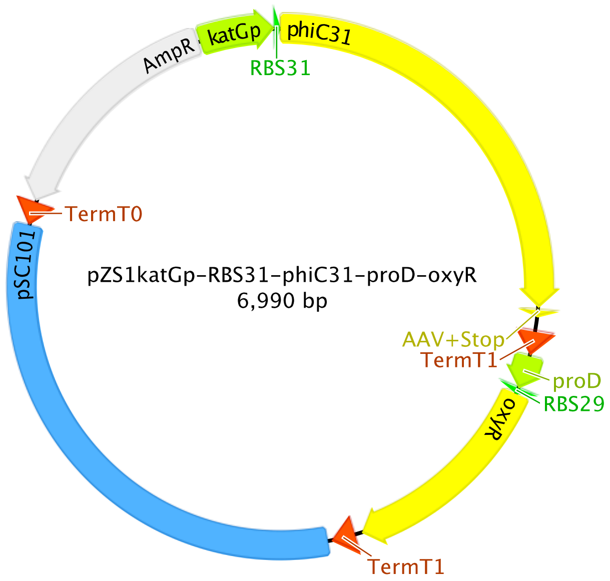
pZS1katGp-RBS31-phiC31-proD-oxyR
6,990 bp
AmpR
katGp
phiC31
RBS31
TermT0
pSC101
AAV+Stop
TermT1
proD
RBS29
oxyR
TermT1

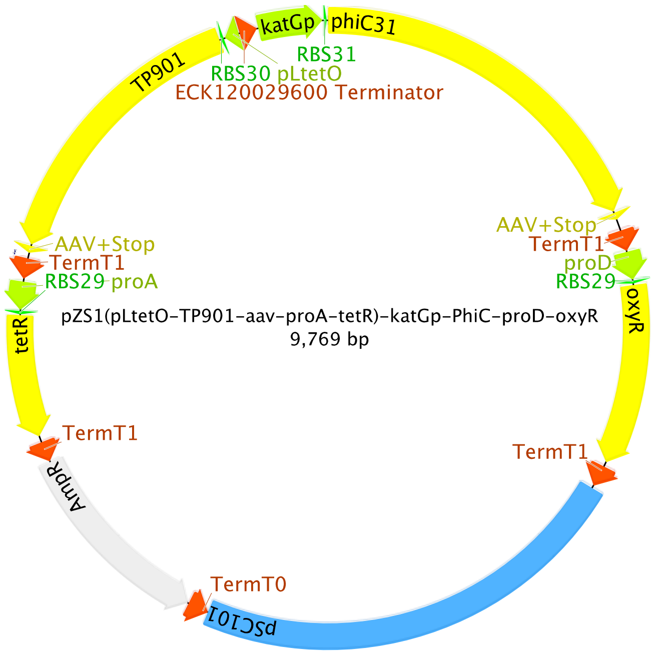
pZS1(pLtetO-TP901-aav-proA-tetR)-katGp-PhiC-proD-oxyR
9,769 bp
katGp
phiC31
TP901
RBS31
RBS30
pLtetO
ECK120029600 Terminator
AAV+Stop
TermT1
RBS29
proA
tetR
AAV+Stop
TermT1
proD
RBS29
oxyR
TermT1
AmpR
TermT1
TermT0
pSC101

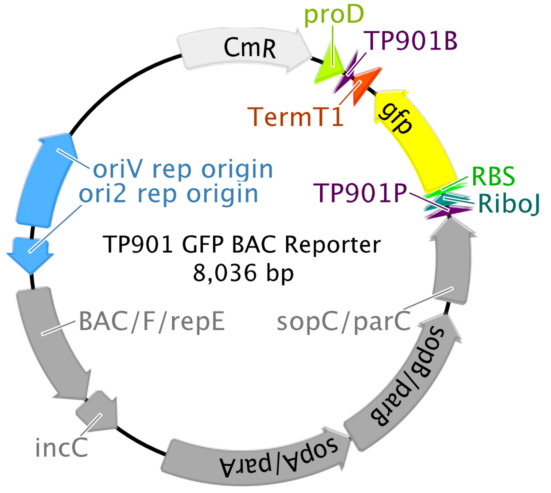
TP901 GFP BAC Reporter
8,036 bp
proD
CmR
TP901B
TermT1
gfp
oriV rep origin
ori2 rep origin
RBS
TP901P
RiboJ
BAC/F/repE
sopC/parC
sopB/parB
incC
sopA/parA

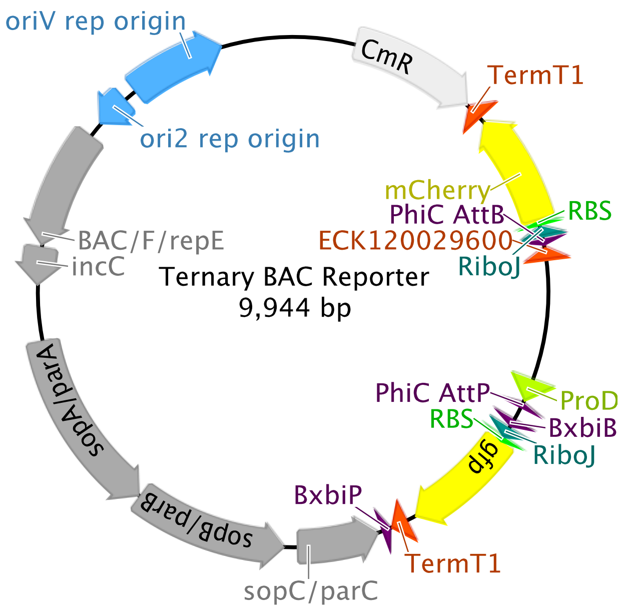
Ternary BAC Reporter
9,944 bp
oriV rep origin
CmR
TermT1
ori2 rep origin
mCherry
PhiC AttB
RBS
ECK120029600
RiboJ
BAC/F/repE
incC
sopA/parA
PhiC AttP
ProD
RBS
BxbiB
RiboJ
gfp
BxbiP
TermT1
sopB/parB
sopC/parC

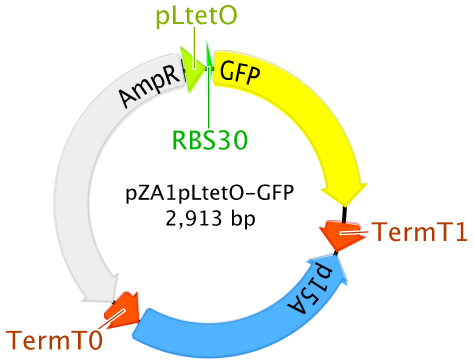
pZA1pLtetO-GFP
2,913 bp
pLtetO
AmpR
GFP
RBS30
TermT1
TermT0
P15A

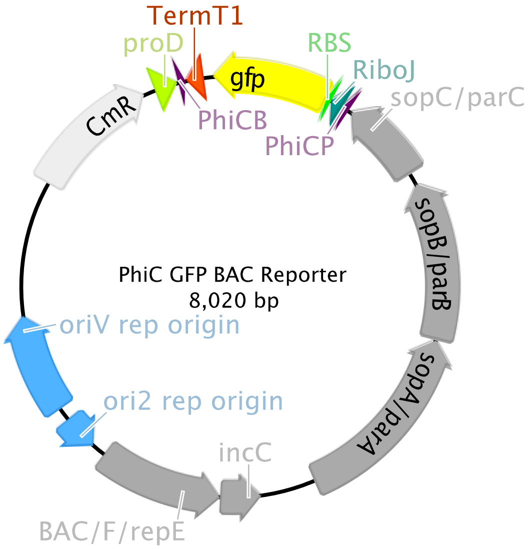
PhiC GFP BAC Reporter
8,020 bp
TermT1
proD
RBS
RiboJ
gfp
sopC/parC
CmR
PhiCB
PhiCP
sopB/parB
oriV rep origin
ori2 rep origin
sopA/parA
incC
BAC/F/repE

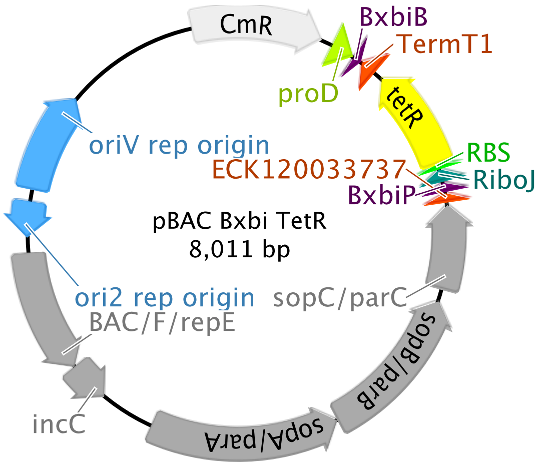
pBAC Bxbi TetR
8,011 bp
CmR
BxbiB
TermT1
proD
tetR
oriV rep origin
RBS
ECK120033737
RiboJ
BxbiP
ori2 rep origin
sopC/parC
BAC/F/repE
sopB/parB
incC
sopA/parA

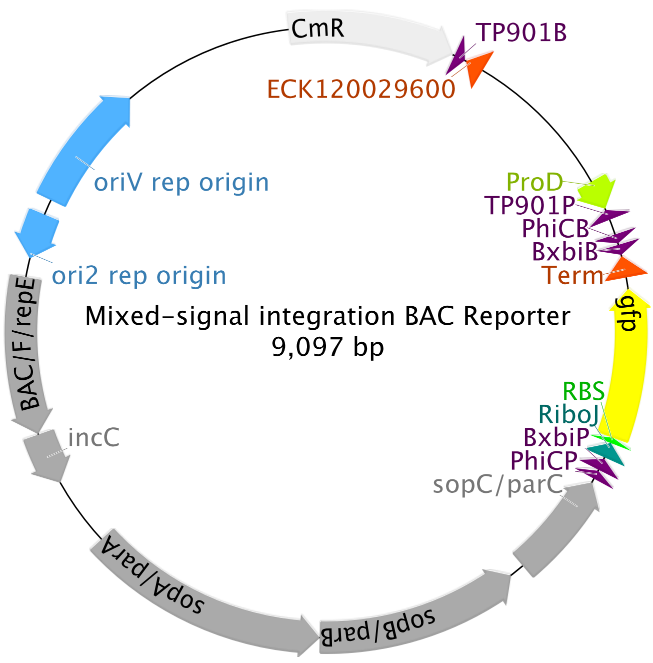

Mixed-signal integration BAC Reporter
9,097 bp
CmR
TP901B
ECK120029600
oriV rep origin
ori2 rep origin
ProD
TP901P
PhiCB
BxbiB
Term
gfp
BAC/F/repE
RBS
RiboJ
BxbiP
PhiCP
incC
sopC/parC
sopA/parA
sopB/parB

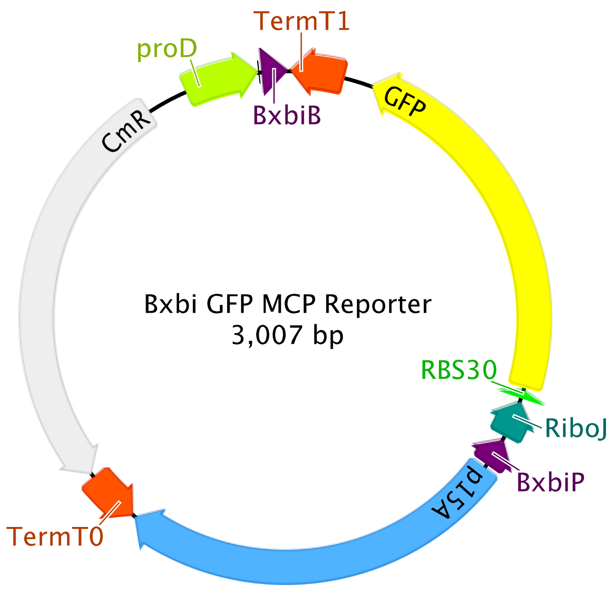

Bxbi GFP MCP Reporter
3,007 bp
TermT1
proD
BxbiB
CmR
GFP
RBS30
RiboJ
BxbiP
p15A
TermT0

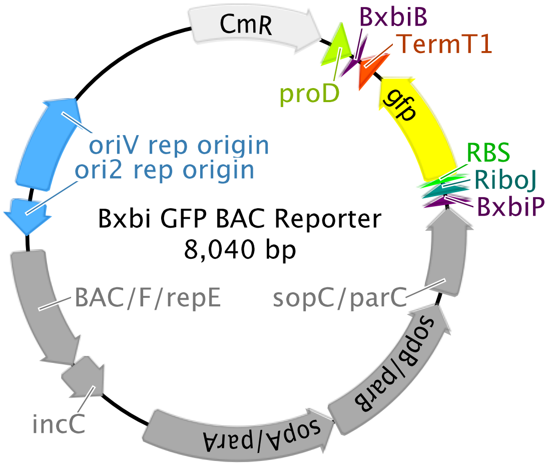

Bxbi GFP BAC Reporter
8,040 bp
CmR
BxbiB
TermT1
proD
gfp
oriV rep origin
ori2 rep origin
RBS
RiboJ
BxbiP
BAC/F/repE
sopC/parC
incC
sopA/parA
sopB/parB

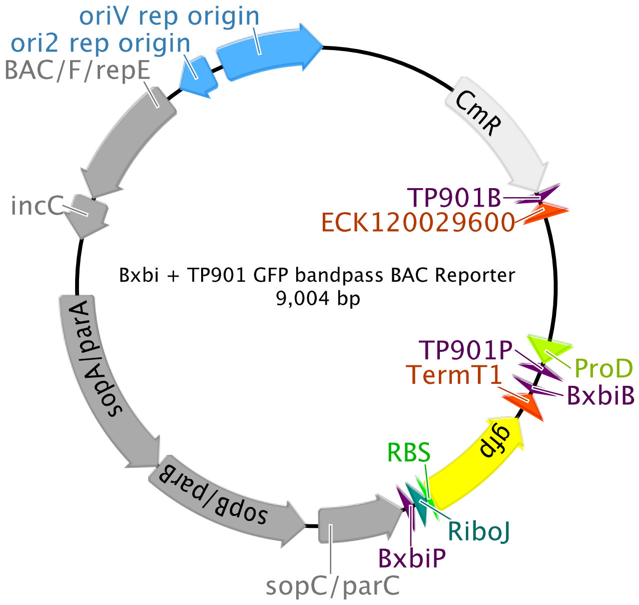

Bxbi + TP901 GFP bandpass BAC Reporter
9,004 bp
oriV rep origin
ori2 rep origin
BAC/F/repE
CmR
incC
TP901B
ECK120029600
sopA/parA
TP901P
TermT1
ProD
BxbiB
gfp
RBS
sopB/parB
RiboJ
BxbiP
sopC/parC

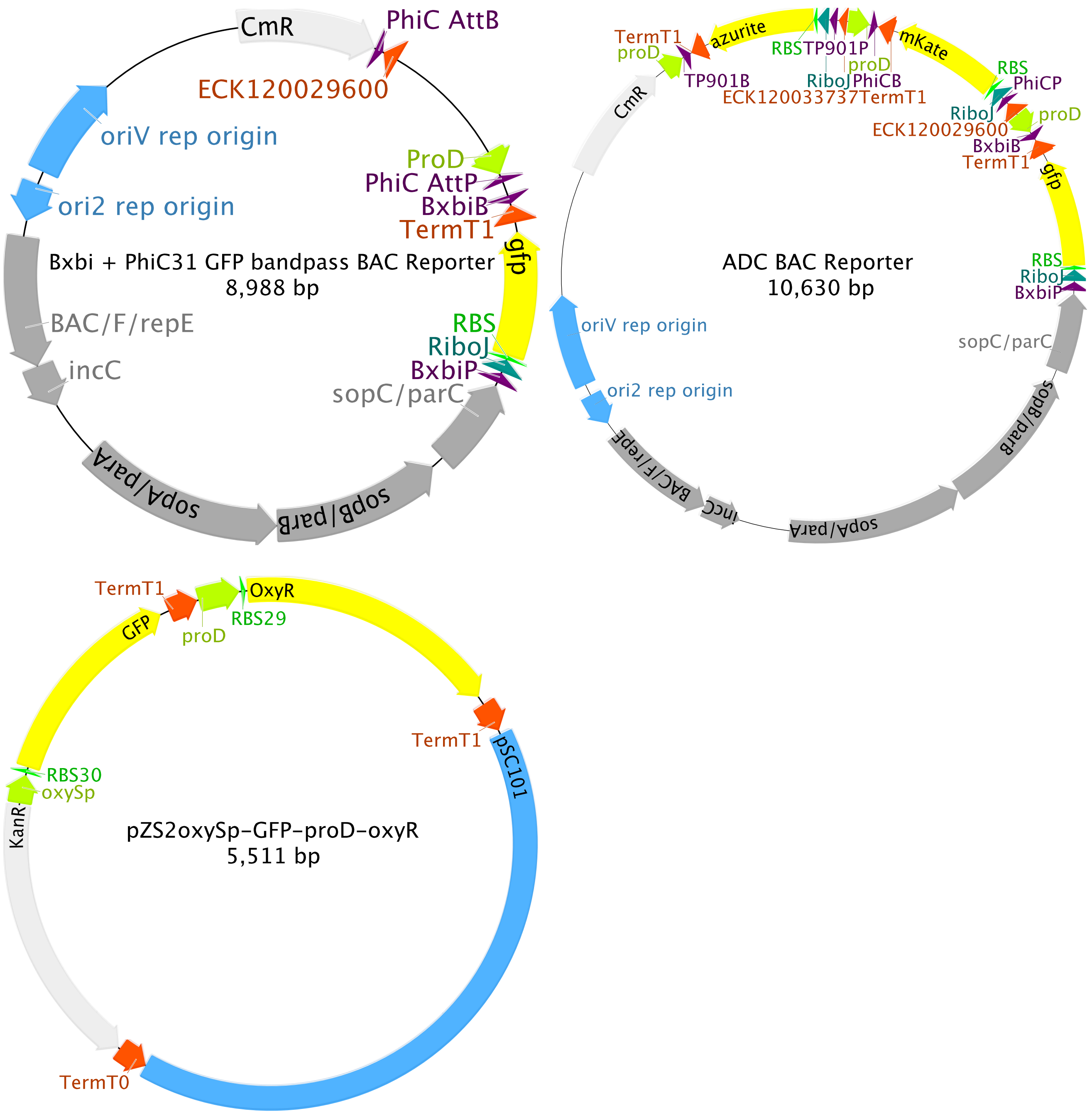

Supplementary Figure 17 | Plasmid maps.

Supplementary Table 1 | List of plasmids used in experiments

**Figure**	**Plasmids**
1a	pZS2oxySp*-RBS30-Bxbi-proD-oxyR + Bxbi GFP BAC Reporter
1c	pZS2katGp-RBS31-PhiC31-proD-oxyR + PhiC31 GFP BAC Reporter
1e	pZS2katGp-RBS33-TP901-proD-oxyR + TP901 GFP BAC Reporter
2a	pZS2oxySp*-RBS30-Bxbi-proD-oxyR + pZS1katGp-RBS31-PhiC31-proD-oxyR + Bxbi+PhiC31 GFP Bandpass BAC Reporter
2c	pZS2oxySp*-RBS30-Bxbi-proD-oxyR + pZS1katGp-RBS33-TP901-proD-oxyR + Bxbi+TP901 GFP Bandpass BAC Reporter
3a	pZS2oxySp*-RBS30-Bxbi-proD-oxyR + pZS1katGp-RBS31-PhiC31-proD-oxyR + Ternary BAC Reporter
3d	pZS1oxySp*-RBS30-bxbi-katGp-RBS31-PhiC31-proD-oxyR + pZS2katGp-RBS33-TP901-proD-oxyR + ADC BAC Reporter
4a	pZS2oxySp*-RBS30-Bxbi-proD-oxyR + pZS1(pLtetO-TP901-aav-proA-tetR)-katGp-PhiC31-proD-oxyR + Mixed-signal integration BAC Reporter
Supplementary Figure 1a	pZS2oxySp-GFP-proD-oxyR
Supplementary Figure 3a	pZS2oxySp*-RBS30-Bxbi-proD-oxyR + Bxbi GFP MCP Reporter
Supplementary Figure 4a	pZS2oxySp*-RBS30-Bxbi-proD-oxyR + pBAC Bxbi TetR + pZA1pLtetO-GFP
Supplementary Figure 7b	pZS2oxySp*-RBS30-Bxbi-proD-oxyR + pZS1katGp-RBS31-PhiC31-proD-oxyR + Bxbi GFP BAC Reporter
Supplementary Figure 7e	pZS2oxySp*-RBS30-Bxbi-proD-oxyR + pZS1katGp-RBS31-PhiC31-proD-oxyR + PhiC31 GFP BAC Reporter
Supplementary Figure 8b	pZS2oxySp*-RBS30-Bxbi-proD-oxyR + pZS1katGp-RBS33-TP901-proD-oxyR + Bxbi GFP BAC Reporter
Supplementary Figure 8e	pZS2oxySp*-RBS30-Bxbi-proD-oxyR + pZS1katGp-RBS33-TP901-proD-oxyR + TP901 GFP BAC Reporter

Supplementary Table 2 | List of synthetic parts

Part Name	Description and Source
oxySp	Promoter for *E. coli oxySp* RNA[1]
katGp	Promoter for *E. coli katG*[1]
ahpCp	Promoter for *E. coli ahpC*[1]
proD	Strong constitutive promoter[2]
proA	Weak constitutive promoter[2]
pLtetO	tetR-regulated lambda phage promoter[3]
RBS30	Ribosome binding site. BBa_B0030[4]
RBS29	Ribosome binding site. BBa_B0029[4]
RBS33	Ribosome binding site. BBa_B0033[4]
RBS31	Ribosome binding site. BBa_B0031[4]
"RBS" with no number	RBS with maximized strength using computational method[8]
RiboJ	Ribozyme-insulator[9]
oxyR	*oxyR* protein-coding sequence[1]
mCherry	mCherry fluorescent protein coding sequence. BBa_J06504[4]
mKate	mKate fluorescent protein coding sequence[5]
azurite	Azurite fluorescent protein coding sequence[6]
gfp	Gfpmut3 fluorescent protein coding sequence. BBa_K863120[4]
Bxb1	Bxb1 serine integrase protein coding sequence[10]
phiC31	PhiC31 serine integrase protein coding sequence[10]
tp901	TP901 serine integrase protein coding sequence[11]
Bxb1B/P	Bxb1 AttB and Bxbi AttP DNA recombination sites[10]
PhiCB/P	PhiC31 AttB and Bxbi AttP DNA recombination sites[10]
TP901B/P	TP901 AttB and Bxbi AttP DNA recombination sites[11]
ECK120029600	Synthetic transcriptional terminator[7]
ECK120033737	Synthetic transcriptional terminator[7]
AAV	AAV degradation tag[4]
TermT1	Transcriptional Terminator T1[3]
TermT0	Transcriptional Terminator T0[3]
p15A	Medium-copy number plasmid origin of replication[3]

pSC101	Low-copy number plasmid origin of replication[3]
ampR	Ampicillin-resistance cassette[3]
kanR	Kanamycin-resistance cassette[3]
cmR	Spectinomycin-resistance cassette[3]
oriV	Trfa-activated plasmid origin of replication[12]
BAC/F/RepE incW parA/B/C	Bacterial artificial chromosome replication factors and origin[12]

Supplementary Table 3 | Fitting parameters used in this study

**Data**	$\boldsymbol{ON_{Max}}$	$\boldsymbol{n}$	$\boldsymbol{K_{on}}$	$\boldsymbol{ON_{Min}}$
Figure 1b, red	93.90	2.603	2.650	4.587
Figure 1d, red	90.22	4.245	11.73	3.208
Figure 1f, red	92.83	3.138	30.61	0.7300
Figure 2b, highpass parameters	94.29	2.623	5.328	1.173
Figure 2b, lowpass parameters	94.88	4.550	19.01	0.3330
Figure 2d, highpass parameters	91.49	2.519	4.519	1.457
Figure 2d, lowpass parameters	94.01	2.434	38.14	0.01933
Figure 3b, green	91.62	-2.512	3.782	0.00889
Figure 3b, red	94.89	3.144	11.04	1.330

Figure 3d, green	90.89	2.684	5.545	4.780
Figure 3d, red	94.24	3.098	15.29	1.720
Figure 3d, blue	91.88	2.900	41.65	2.727
Figure 4b	Same as Figure 2b	Same as Figure 2b	Same as Figure 2b	Same as Figure 2b
Supplementary figure 1b	28628	1.515	163.2	13.50
Supplementary figure 3b, black	82.31	2.155	1.719	18.07
Supplementary figure 3b, red	93.99	2.669	2.676	5.613
Supplementary figure 4b	92.96	-3.008	2.861	0.2667
Supplementary figure 15, green	90.94	2.461	2.051	2.287
Supplementary figure 15, red	94.75	2.085	4.983	-0.3013
Supplementary figure 15, blue	86.67	2.468	18.80	2.814

Supplementary Note 1: Data Processing and Calculations

Calculating the sigmoidal fit, input threshold, and relative input range

The data from the BAC circuit in **Supplementary Fig. 3** is shown as an example.

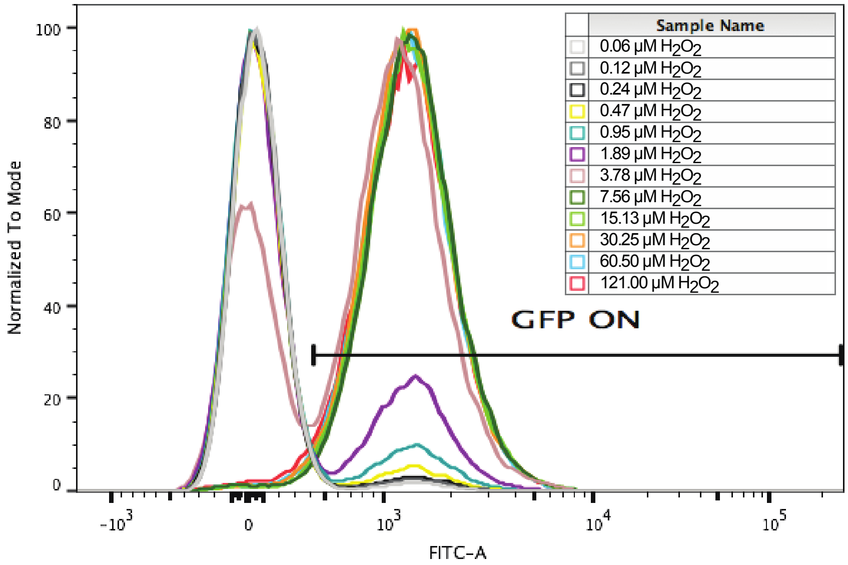

1. Calculate % of cells at each concentration of H_2O_2 that fall within the "GFP ON" gate. The "GFP ON" gate is drawn for each experiment at the FITC fluorescence level where the fluorescence distribution of uninduced cells intersects with the fluorescence distribution of induced cells, or in between the uninduced and induced distributions when the fluorescence distributions are well-resolved and do not overlap**.** Take the average %ON of biological replicates to calculate the mean and standard deviation (plotted).

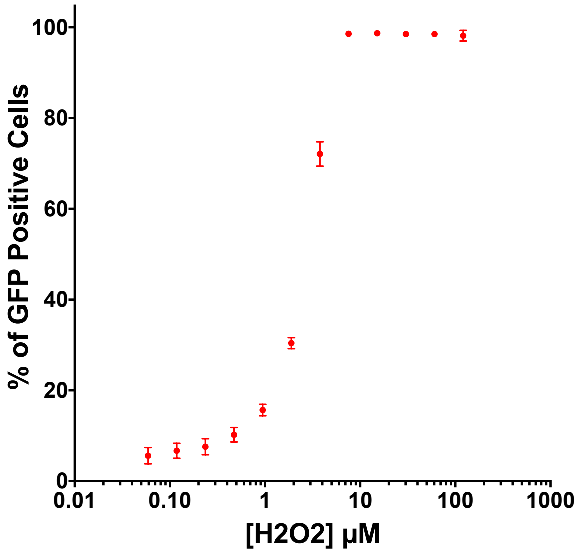

2. To derive the transfer function, fit the mean %ON vs. H_2O_2 concentration data to a Hill-like sigmoidal function (solid line below):

$$\%ON = ON_{Max}\frac{[H_2O_2]^n}{[H_2O_2]^n + (K_{on})^n} + ON_{Min}$$

Where $[H_2O_2]$ is the independent variable, ON_{Min} is the empirically observed minimum percent ON, and ON_{Max}, K_{on}, and n are fit to the data.

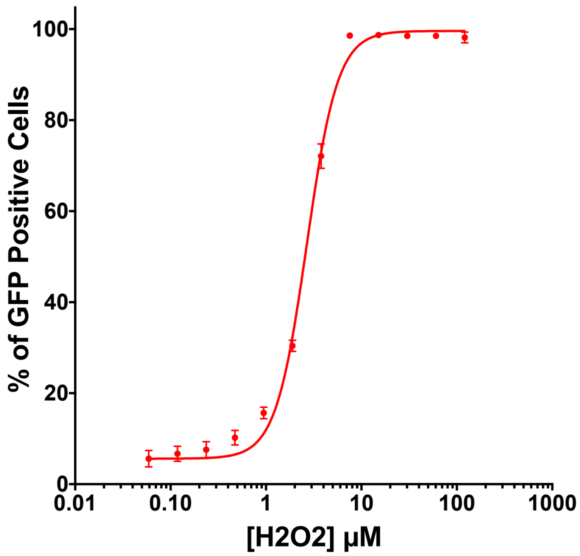

3. The input dynamic range (transition band, shaded red below) is defined as the input H_2O_2 concentration span that yields 10% ON to 90% ON, as interpolated from the transfer function:

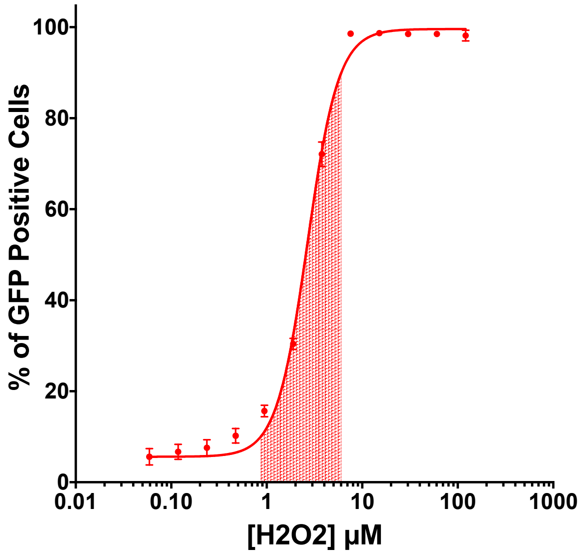

4. Calculate the relative input range from the 10% ON and 90% ON input values:

$$Relative\ Input\ Range\ (RIR) = \frac{[H_2O_2]_{90\%}}{[H_2O_2]_{10\%}}$$

Calculating the fit to a bandpass filter circuit

The fit to a bandpass filter circuit (black line in **Fig. 2b, 2d, 4b**) was derived by subtracting the transfer function of the low-pass comparator (**Supplementary Fig. 7f or 8f**) from the transfer function of the high-pass comparator (**Supplementary Fig. 7c or 8c**):

$$\%ON = ON_{Max,hp}\frac{[H_2O_2]^{n,hp}}{[H_2O_2]^{n,hp} + (K_{on,hp})^{n,hp}} + ON_{Min,hp} - ON_{Max,lp}\frac{[H_2O_2]^{n,lp}}{[H_2O_2]^{n,lp} + (K_{on,hp})^{n,lp}} - ON_{Min,lp}$$

Where *hp* subscript denotes a variable from the "high pass" circuit and *lp* subscript denotes a variable from the "low pass" circuit.

Calculating the relative resolution of a genetic analog-to-digital converter circuit

We defined the relative resolution (*RQ*) as:

$$RQ = \frac{ADC\ RIR}{2^{bits} - 2}$$

Where the *ADC RIR* is:

$$\frac{[H_2O_2]_{50\%,high}}{[H_2O_2]_{50\%,low}}$$

$[H_2O_2]_{50\%,high}$ is the concentration of H_2O_2 necessary for 50% of cells to turn ON for the highest threshold comparator in the ADC, and $[H_2O_2]_{50\%,low}$ is the concentration of H_2O_2 necessary for 50% of cells to turn ON for the lowest threshold comparator in the ADC.

The number of bits is the total number of bits encoded by the ADC (in the case of **Fig. 3d-f**, it is 2 bits). We subtract 2 in the denominator because 2 of the states are encoded outside of the ADC RIR (i.e., below the $[H_2O_2]_{50\%,low}$concentration and above the $[H_2O_2]_{50\%,high}$ concentration, states 000 and 111).

References

1. The EcoCyc Database, (available at http://ecocyc.org/)

2. Davis, J., Rubin, A. & Sauer, R. Design, construction and characterization of a set of insulated bacterial promoters. *Nucleic Acids Research* 39, 1131-1141 (2010).

3. Lutz, R. Independent and tight regulation of transcriptional units in Escherichia coli via the LacR/O, the TetR/O and AraC/I1-I2 regulatory elements. *Nucleic Acids Research* 25, 1203-1210 (1997).

4. Registry of Standard Biological Parts, (available at http://partsregistry.org/).

5. Shcherbo, D. et al. Far-red fluorescent tags for protein imaging in living tissues. *Biochem. J.* 418, 567 (2009).

6. Mena, M., Treynor, T., Mayo, S. & Daugherty, P. Blue fluorescent proteins with enhanced brightness and photostability from a structurally targeted library. *Nat Biotechnol* 24, 1569-1571 (2006).

7. Chen, Y. et al. Characterization of 582 natural and synthetic terminators and quantification of their design constraints. *Nat Meth* 10, 659-664 (2013).

8. Salis, H. M., Mirsky, E. A. & Voigt, C. A. Automated design of synthetic ribosome binding sites to control protein expression. *Nat. Biotechnol.* **27,** 946–950 (2009).

9. Lou, C., Stanton, B., Chen, Y.-J., Munsky, B. & Voigt, C. A. Ribozyme-based insulator parts buffer synthetic circuits from genetic context. *Nat. Biotechnol.* **30,** 1137–1142 (2012).

10. Siuti, P., Yazbek, J. & Lu, T. K. Synthetic circuits integrating logic and memory in living cells. *Nat. Biotechnol.* **31,** 448–452 (2013).

11. Bonnet, J., Yin, P., Ortiz, M. E., Subsoontorn, P. & Endy, D. Amplifying genetic logic gates. *Science (80-.).* **340,** 599–603 (2013).

12. Wild, J., Hradecna, Z. & Szybalski, W. Conditionally Amplifiable BACs : Switching From Single-Copy to High-Copy Vectors and Genomic Clones. *Genome Res.* **12,** 1434–1444 (2002).
